# A Cullin 5-based complex serves as an essential modulator of ORF9b stability in SARS-CoV-2 replication

**DOI:** 10.1038/s41392-024-01874-5

**Published:** 2024-06-28

**Authors:** Yuzheng Zhou, Zongpeng Chen, Sijie Liu, Sixu Liu, Yujie Liao, Ashuai Du, Zijun Dong, Yongxing Zhang, Xuan Chen, Siyi Tao, Xin Wu, Aroona Razzaq, Gang Xu, De-an Tan, Shanni Li, Youwen Deng, Jian Peng, Shuyan Dai, Xu Deng, Xianwen Zhang, Taijiao Jiang, Zheng Zhang, Gong Cheng, Jincun Zhao, Zanxian Xia

**Affiliations:** 1 Department of Cell Biology, School of Life Sciences, Central South University, 410013 Changsha, China; 2 Institute for Hepatology, National Clinical Research Center for Infectious Disease, Shenzhen Third People’s Hospital, The Second Affiliated Hospital, School of Medicine, Southern University of Science and Technology, 518112 Shenzhen, China; 3 Department of Basic Medicine, School of Medicine, Hunan Normal University, 410081 Changsha, China; 4 Department of spine surgery, The Third Xiangya Hospital, Central South University, 410013 Changsha, China; 5 School of Basic Medical Sciences, Anhui Medical University, 230032 Hefei, China; 6 Hunan Key Laboratory of Neurorestoratology, 921 Hospital of Joint Logistics Support Force People’s Liberation Army of China (The Second Affiliated Hospital of Hunan Normal University), 410003 Changsha, Hunan China; 7 Department of Geriatric Surgery, Xiangya Hospital, Central South University, 410008 Changsha, China; 8 Xiangya School of Pharmaceutical Sciences, Central South University, 410013 Changsha, China; 9 Institute of Infectious Diseases, Shenzhen Bay Laboratory, 518132 Shenzhen, China; 10 Guangzhou Laboratory, 510005 Guangzhou, China; 11 Tsinghua University-Peking University Joint Center for Life Sciences, School of Medicine, Tsinghua University, 100084 Beijing, China; 12 State Key Laboratory of Respiratory Disease, National Clinical Research Center for Respiratory Disease, Guangzhou Institute of Respiratory Health, The First Affiliated Hospital of Guangzhou Medical University, 510120 Guangzhou, China; 13 Hunan Key Laboratory of Animal Models for Human Diseases, Hunan Key Laboratory of Medical Genetics & Center for Medical Genetics, School of Life Sciences, Central South University, 410008 Changsha, China

**Keywords:** Molecular biology, Infection, Microbiology

## Abstract

The ORF9b protein, derived from the nucleocapsid’s open-reading frame in both SARS-CoV and SARS-CoV-2, serves as an accessory protein crucial for viral immune evasion by inhibiting the innate immune response. Despite its significance, the precise regulatory mechanisms underlying its function remain elusive. In the present study, we unveil that the ORF9b protein of SARS-CoV-2, including emerging mutant strains like Delta and Omicron, can undergo ubiquitination at the K67 site and subsequent degradation via the proteasome pathway, despite certain mutations present among these strains. Moreover, our investigation further uncovers the pivotal role of the translocase of the outer mitochondrial membrane 70 (TOM70) as a substrate receptor, bridging ORF9b with heat shock protein 90 alpha (HSP90α) and Cullin 5 (CUL5) to form a complex. Within this complex, CUL5 triggers the ubiquitination and degradation of ORF9b, acting as a host antiviral factor, while HSP90α functions to stabilize it. Notably, treatment with HSP90 inhibitors such as GA or 17-AAG accelerates the degradation of ORF9b, leading to a pronounced inhibition of SARS-CoV-2 replication. Single-cell sequencing data revealed an up-regulation of HSP90α in lung epithelial cells from COVID-19 patients, suggesting a potential mechanism by which SARS-CoV-2 may exploit HSP90α to evade the host immunity. Our study identifies the CUL5-TOM70-HSP90α complex as a critical regulator of ORF9b protein stability, shedding light on the intricate host–virus immune response dynamics and offering promising avenues for drug development against SARS-CoV-2 in clinical settings.

## Introduction

After nearly 4 years of the pandemic, the severe acute respiratory syndrome coronavirus 2 (SARS-CoV-2) is displaying increased infectiousness and a propensity for immune evasion. The virus comprises a single-stranded positive-sense RNA capable of encoding 4 structural proteins, 16 non-structural proteins, and various accessory proteins.^1^ The nucleocapsid (N) protein, the most abundant protein in coronaviruses,^2^ is a highly immunogenic phosphoprotein with a very conservative gene sequence,^3^ frequently used as a diagnostic marker.^4,5^ Notably, an accessory protein, ORF9b, is translated from an alternative open-reading frame within the *N* gene. Reports indicate rapid accumulation of ORF9b in COVID-19 patients within 24 h of initial infection,^6,7^ concurrent with observed attenuation of IFNβ production during this period, suggesting that ORF9b protein plays an important role in antagonizing host immunity.^8^ More studies have demonstrated that ORF9b protein can impede the innate immune response by interrupting the ubiquitination of NEMO, binding to the mitochondrial import receptor subunit TOM70, or interacting with other components of the innate immune pathway. These interactions contribute to the virus’s immune evasion.^8–12^ Furthermore, a recent report showed that ORF9b can undergo degradation through the ubiquitin-proteasome system (UPS).^13^ However, the intricate regulatory mechanisms and clinical implications of this process remain to be fully elucidated.

Ubiquitination, a prevalent posttranslational modification of proteins, involves an enzymatic cascade where ubiquitin molecules are covalently bound to substrate proteins by E1 ubiquitin-activating enzymes, E2 ubiquitin-binding enzymes, and E3 ubiquitin ligases.^14^ This process is employed by numerous viruses to counteract the host’s innate immune response, either by inducing degradation^15–18^ or inhibiting the activation^8,19–21^ of key proteins. Conversely, hosts utilize ubiquitination to combat viruses by triggering the immune response, inducing degradation, or modifying the functions of viral proteins.^22–24^ Thus, ubiquitination serves as a double-edged sword in virus–host interactions, with both parties exploiting it for their purposes. In SARS-CoV-2 infection, an increase in ubiquitination is observed.^25^ Among the three enzymes, E3s play a critical role in determining the specificity of substrate ubiquitination. Some E3 ligases have been identified as anti-coronavirus host factors by inducing viral protein degradation.^26–29^ Notably, the Cullin–RING (really interesting new gene) ligases (CRLs) form the largest E3 family, which is evolutionarily conserved and plays crucial roles in many biological processes, including virus infection.^30–33^ In humans, the Cullin family comprises eight members: CUL1 (Cullin 1), CUL2, CUL3, CUL4A, CUL4B, CUL5, CUL7, and CUL9.^34^ Within the Cullin-RING ligase 5 (CRL5) complex, the molecular scaffold CUL5 binds RBX2 (RING-box protein) at the N-terminal to recruit E2 enzyme and adaptor proteins Elongin B/C at the C-terminal, which then links a substrate receptor protein SOCS for substrate recognition.^35^ Interestingly, heat shock protein 90 (HSP90), a member of the HSP family, also functions as a substrate receptor in CRL5, independent of Elongin B/C.^36,37^

HSP90 is a widely distributed chaperone protein in eukaryotic cells, playing a crucial role in preventing protein misfolding, aggregation, or degradation. Human HSP90 comprises five isoforms, with HSP90α and HSP90β being the most prominent cytoplasmic isoforms that form homodimers.^38,39^ Elevated expression of HSP90 is often observed in certain malignancies, where it contributes to protecting tumor growth in adverse environments. Thus, inhibitors or derivatives that target HSP90 enzyme activity can effectively block numerous oncogenic signaling pathways, making them pivotal players in cancer therapy.^40,41^ Furthermore, recent studies have uncovered the antiviral effects of HSP90 inhibitors against coronaviruses, including SARS-CoV, MERS-CoV and SARS-CoV-2.^42–45^ In the case of MERS-CoV, these inhibitors trigger the degradation of N protein as part of their antiviral mechanism,^44^ while in SARS-CoV-2, N, M, and a few other viral proteins have been identified as the downstream targets of HSP90 inhibitors.^45^

Viruses heavily rely on host factors to facilitate entry, replication, and evasion of the innate immune response. Understanding the mechanisms behind virus–host interactions is crucial for comprehending viral infections, pathogenic processes, and the development of antiviral therapies. While interaction networks between viral proteins and host proteins,^46–48^ as well as viral RNA and host proteins,^49–51^ have been extensively investigated, much remains unknown regarding the specific mechanisms and their impacts on both the host and the virus. Our study has unveiled the previously unknown impact of a newly identified complex composed of CUL5, TOM70, and HSP90α, termed CUL5-TOM70-HSP90α, on the stability of the SARS-CoV-2 ORF9b protein. TOM70, which is known to interact with ORF9b and HSP90, acts as a substrate receptor, while CUL5 functions as a novel host anti-SARS-CoV-2 factor by promoting the degradation of ORF9b. Additionally, we have found that HSP90 inhibitors geldanamycin (GA) or Tanespimycin (17-AAG) can enhance the ubiquitinated degradation of ORF9b and attenuate the replication capacity of SARS-CoV-2. Our data unveils the ability of SARS-CoV-2 to stabilize its own ORF9b protein through HSP90α, which is prominently expressed in various cells of patients with respiratory and gastrointestinal basic diseases,^52,53^ potentially explaining the heightened susceptibility of these patients to severe symptoms upon infection. Moreover, our findings shed light on how HSP90 inhibitors counteract SARS-CoV-2 replication, offering a promising strategy and target for clinical adjuvant therapy against COVID-19. In essence, the regulation of ORF9b stability by the CUL5-TOM70-HSP90α complex underscores the battle between the host and SARS-CoV-2.

## Results

### K48-linked polyubiquitin chain at K67 site mediates the ubiquitinated degradation of SARS-CoV-2 ORF9b

Although the *N* genes of both SARS-CoV and SARS-CoV-2 contain an intrinsic open-reading frame that translates an accessory protein ORF9b (Supplementary Fig. 1a), ORF9b is a distinct protein by difference in codon usage, amino acid sequence, and structure compared to the N protein (Supplementary Fig. 1b–f).^54^ ORF9b is believed to be a viral factor against host immunity.^55^ Our data further confirmed that ORF9b of both SARS-CoV and SARS-CoV-2 can significantly inhibit the production of IFNβ (Supplementary Fig. 2a). Furthermore, both ORF9b proteins can inhibit the expression of interferon-stimulated genes such as *ISG56* and *MX1*, as well as the production of antiviral cytokines such as CCL5 and CXCL10 (Supplementary Fig. 2b). We also found that the ability of ORF9b to antagonize host intrinsic immunity is related to its expression levels (Supplementary Fig. 2c, d).

**Fig. 1:**
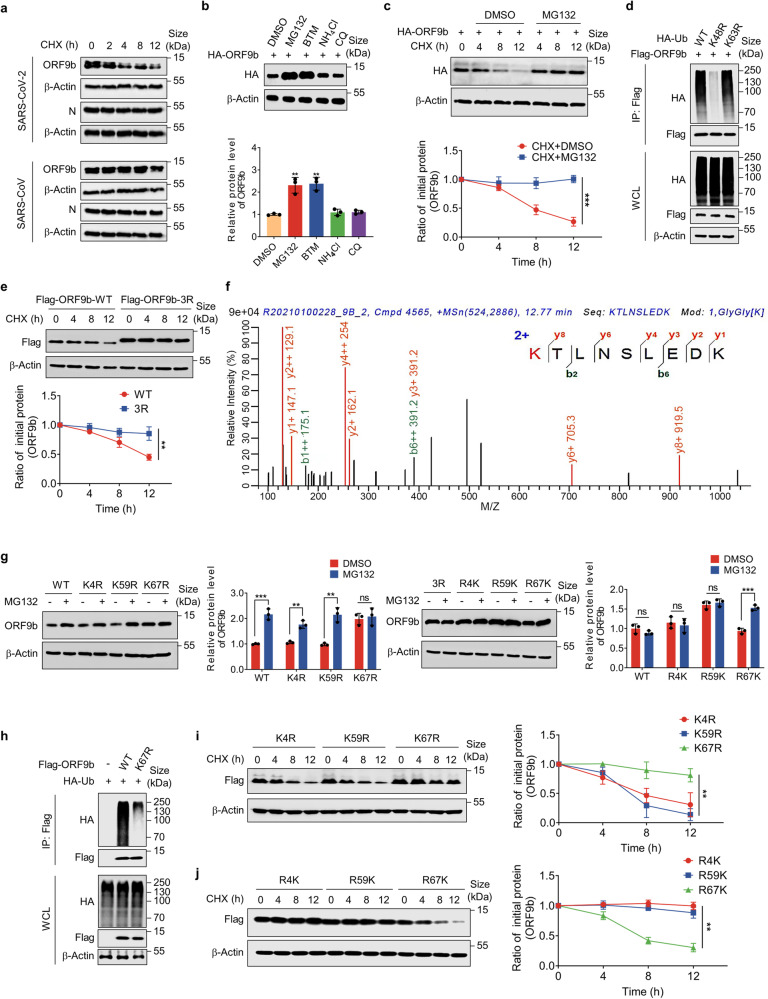
K48-linked polyubiquitin chain at the K67 site mediates the ubiquitinated degradation of SARS-CoV-2 ORF9b. **a** Half-life analyses of N and ORF9b proteins of SARS-CoV and SARS-CoV-2 in HEK293T cells. The cells were transfected with plasmids expressing the aforementioned viral proteins for 30 h and then split into 12-well plates. Following treatment with CHX, cells were collected as indicated time points for Western blot analysis. **b** The protein levels of ORF9b in HEK293T cells with treatment of DMSO, MG132, BTM, CQ, and NH_4_Cl. **c** Half-life analyses of SARS-CoV-2 ORF9b in the primary human airway epithelial (HAE) cells with treatment of CHX + MG132 or CHX + DMSO. **d** In vivo ORF9b ubiquitination assay in HEK293T cells. The HA-Ub WT, K48R, and K63R mutant plasmids were individually co-transfected with the Flag-ORF9b plasmid into HEK293T cells, followed by culturing and co-immunoprecipitation. Western blot was performed to detect ubiquitinated chains. **e** Half-life analyses of SARS-CoV-2 ORF9b-WT and SARS-CoV-2 ORF9b-3R mutant in HEK293T cells. **f** Flag-ORF9b was expressed in HEK293T and purified by anti-Flag beads, and then analyzed by mass spectrometry. One peptide containing lysine residues was identified. K67 was shown in red. **g** The protein levels of SARS-CoV-2 ORF9b-WT and indicated mutants expressed in HEK293T cells were detected after treatment with MG132 or DMSO. **h** In vivo ubiquitination assay of SARS-CoV-2 ORF9b-WT and SARS-CoV-2 ORF9b-K67R mutant in HEK293T cells. **i** and **j** Half-life analyses of indicated SARS-CoV-2 ORF9b mutants in HEK293T cells. Quantification was shown as mean±s.d. *n* = 3 independent experiments. Student’s *t*-test (unpaired, two-tailed) was used to compare two independent groups, and a two-way ANOVA test was performed for comparisons of multiple groups. **P < 0.01; ***P < 0.001; n.s. not significant

To further explore the function and protein properties of ORF9b, we pulled down the overexpressed Flag-ORF9b protein from HEK293T cells and performed mass spectrometry (MS) to identify the host-interacting proteins (Supplementary Fig. 3a). KEGG enrichment analysis on the identified proteins showed that the interacting proteins of SARS-CoV-2 ORF9b were significantly enriched in the proteasome pathway, while SARS-CoV ORF9b did not have this property (Supplementary Fig. 3b, c). We then created a heatmap based on the number of interacting protein peptides enriched in the proteasome pathway to visualize the differences between SARS-CoV and SARS-CoV-2 (Supplementary Fig. 3d and Supplementary Table 1). This implies that ORF9b of SARS-CoV-2 is likely an unstable protein degraded by the proteasome compared to that of SARS-CoV. Indeed, during our manuscript preparation, Gao et al. reported that SARS-CoV-2 ORF9b is degraded via the UPS, although the E3 ubiquitin ligase was not identified.^13^ To further investigate this, we performed a comparative analysis of the half-life of N and ORF9b proteins. We treated HEK293T cells overexpressing the N and ORF9b proteins from both SARS-CoV and SARS-CoV-2 with the protein synthesis inhibitor cycloheximide (CHX) and collected the cells at different time points (0, 2, 4, 8, and 12 h). The results showed that SARS-CoV-2 ORF9b protein levels decreased with increasing time, whereas SARS-CoV ORF9b and N, as well as SARS-CoV-2 N protein levels, did not change significantly (Fig. 1a). These findings support that the SARS-CoV-2 ORF9b is an unstable protein with a relatively short half-life in host cells compared to the other three proteins. The results of the treatments with the proteasome inhibitors MG132 and bortezomib (BTM) and lysosome inhibitors NH_4_Cl and chloroquine (CQ) further strengthen the evidence that SARS-CoV-2 ORF9b is degraded through the ubiquitin-proteasome system (Fig. 1b). SARS-CoV-2 can infect various host cells in the respiratory system, intestinal system, and the liver. Consistent with this observation,^56^ the ORF9b protein exhibited a relatively short half-life in a wide range of cells, including the primary human airway epithelial (HAE) cells, HEK293T, Vero, human lung-associated cells (BEAS-2B, A549, and Calu3), human colon cancer cells (HCT116), and human liver cancer cells (Caco2 and Huh7) (Fig. 1c and Supplementary Fig. 4a). To avoid phenotypic interference caused by different promoters, we assessed the stability of ORF9b under the control of the CMV, CAG, and TRE promoters. Our results indicated that although there may be slight variations in the half-life of ORF9b with different promoters, all promoter variants demonstrate a notable reduction in ORF9b protein levels over time following CHX treatment (Supplementary Fig. 4b).

While various mutant strains of SARS-CoV-2, including Gamma, Delta and Omicron (Supplementary Fig. 4c), exhibit mutations in the ORF9b protein,^55,57^ the half-life experiments indicated that the UPS can still intracellularly degrade ORF9b proteins in these strains (Supplementary Fig. 4d). Further, in vivo ubiquitination experiments revealed that ORF9b could form polyubiquitination chains dominated by lysine 48 (K48)-type (Fig. 1d). These results provided further evidence that ORF9b protein can be ubiquitinated in host cells and subsequently degraded by the proteasome.

Although the ORF9b proteins from SARS-CoV and SARS-CoV-2 share over 73% identity, our results have shown that the SARS-CoV ORF9b protein is comparatively stable in contrast to SARS-CoV-2 ORF9b. Ubiquitination generally occurs on the lysine residues of a target protein. By analyzing the amino acid sequences of the two coronavirus ORF9b proteins, we discovered three specific lysine residues (K4, K59, and K67) in SARS-CoV-2 ORF9b protein that are absent in SARS-CoV ORF9b protein (Supplementary Fig. 3e). After mutating all three lysine sites to arginine (ORF9b-3R), we found that ORF9b-3R was considerably more stable than ORF9b-WT (wild type) and no longer degraded within 12 h after CHX treatment (Fig. 1e). This indicates that the ubiquitination sites of SARS-CoV-2 ORF9b protein exist in these three lysine residues. We further collated the ORF9b-related peptides identified by mass spectrometry and found that K67 might be the key site for the ubiquitination of ORF9b (Fig. 1f).

To further verify this, we mutated the aforementioned three lysine residues to arginine on ORF9b-WT, naming them K4R, K59R, and K67R respectively. We also performed revertant mutations on ORF9b-3R, naming them R4K, R59K, and R67K. We treated cells overexpressing the corresponding mutant ORF9b with MG132 and found that the protein level of K67R was not significantly changed after treatment with MG132, while the protein levels of WT, K4R, and K59R were significantly increased. In contrast to 3R, R4K, and R59K, the protein level of R67K was increased with MG132 treatment (Fig. 1g). In vivo ubiquitination assays showed that K67R formed significantly fewer polyubiquitinated chains in cells compared to WT (Fig. 1h). The half-life of K4R and K59R decreased over time, while K67R protein levels remained stable when treated with CHX. Meanwhile, the protein levels of R4K and R59K remained unchanged, while R67K protein levels decreased continuously (Fig. 1i, j). These results demonstrate that the lysine residue at K67 is the site for the ubiquitination of ORF9b protein.

### CUL5 induces the degradation of ORF9b

**Fig. 2:**
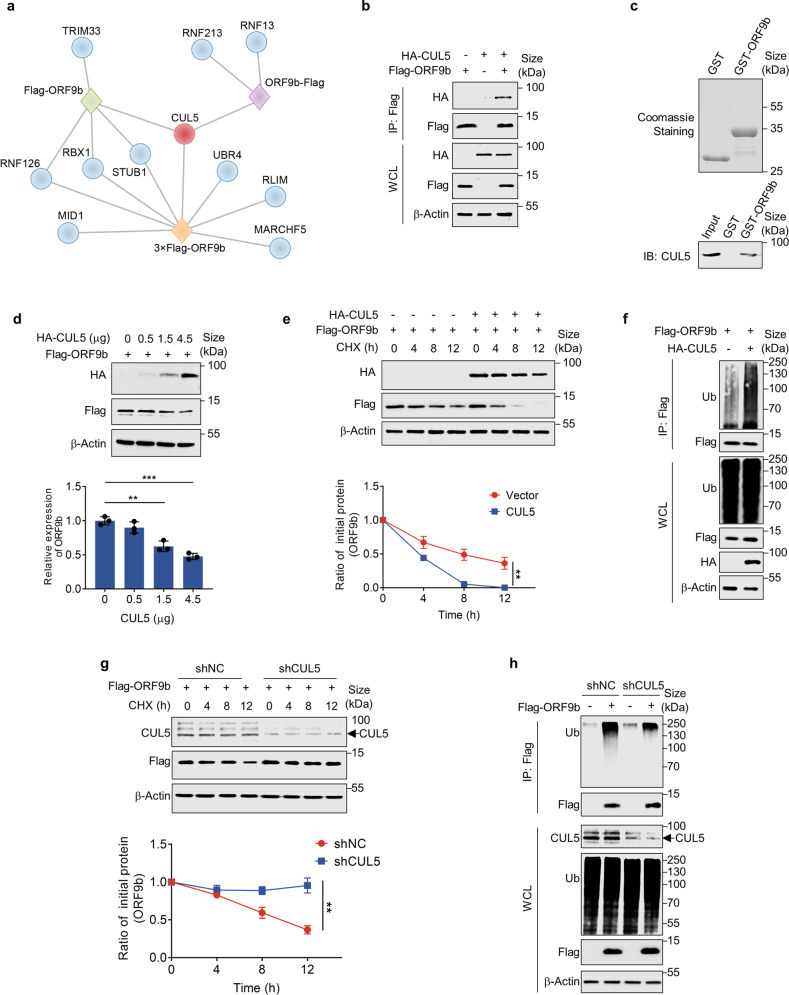
CUL5 induces the degradation of ORF9b. **a** The interacting proteins co-precipitated with different tagged ORF9b were identified by mass spectrometry. A protein–protein interaction network was constructed based on the identified E3 ligases. **b** Co-Immunoprecipitation was performed to test the interaction between CUL5 and ORF9b in HEK293T cells. The HEK293T cells were co-transfected with plasmids containing Flag-ORF9b and HA-CUL5. WCLs and precipitated proteins were analyzed by immunoblotting with indicated antibodies. **c** GST and GST-ORF9b were purified from *E. coli* and analyzed by Coomassie staining. **d** The HEK293T cells overexpressing the ORF9b protein were transfected with a gradually increasing amount of plasmids containing CUL5. Cells were collected for Western blot. **e** Half-life analyses of SARS-CoV-2 ORF9b were performed when CUL5 was overexpressed or not. **f** In vivo ORF9b ubiquitination assay when overexpressing CUL5 or not. **g** Half-life analyses of SARS-CoV-2 ORF9b when knocking down CUL5 or not. **h** In vivo ORF9b ubiquitination assay when CUL5 was knocked down or not in HEK293T cells. Quantification was shown as mean ± s.d. *n* = 3 independent experiments. *P < 0.05; **P < 0.01; ***P < 0.001. Student’s *t*-test (unpaired, two-tailed) was used to compare two independent groups, and a two-way ANOVA test was performed for comparisons of multiple groups. *P < 0.05; **P < 0.01; ***P < 0.001

After demonstrating that SARS-CoV-2 ORF9b can be degraded by the UPS, we aimed to identify the key E3 ligase that specifically regulates its ubiquitination and degradation. To minimize any potential influence of tagging on the relatively small-sized ORF9b protein and enhance the purification efficiency, we generated three distinct tagged-ORF9b proteins: Flag-ORF9b, ORF9b-Flag, and 3×Flag-ORF9b. Subsequently, we isolated the ORF9b protein under cell culture conditions with MG132 treatment. Through mass spectrometry, we identified potential host-interacting proteins (Supplementary Fig. 5a and Supplementary Table 2). KEGG enrichment analysis showed that the co-precipitated proteins of all three tagged ORF9b proteins were enriched in the proteasome pathway (Supplementary Fig. 5b–d). By analyzing the number of unique peptides corresponding to identified E3 ligases or the E3 complex subunits, we constructed a protein–protein interaction network and heatmap. Although the host-interacting proteins varied among the different Flag tag groups, possibly due to the tag influence or experimental variations, CUL5 was enriched in each group, suggesting it is the most likely candidate to interact with ORF9b and regulate its ubiquitinated degradation (Fig. 2a).

CUL5 serves as a scaffold, binding to adaptor proteins Elongin B/C, which act as the main substrate receptor (with the SOCS family predominantly), at the N-terminus and to a RING protein RBX1 or RBX2 at the C-terminus. Together, these proteins form the E3 ubiquitin ligase complex known as Cullin-RING ligase 5 (CRL5), which recruits E2 and facilitates the transfer of ubiquitin from E2 to the substrate.^58,59^ Therefore, we explored the impact of CUL5 on ORF9b ubiquitination and stability. Firstly, we demonstrated that viral ORF9b protein could interact with CUL5 by co-immunoprecipitation and GST pull-down (Fig. 2b, c). Gradually increasing the expression level of CUL5 led to a proportional decrease in the ORF9b protein level (Fig. 2d), and the half-life of ORF9b protein was notably shorter than that of the empty vector group (Fig. 2e). Meanwhile, overexpression of CUL5 significantly enhanced the formation of polyubiquitinated chains of ORF9b protein in cells (Fig. 2f). Conversely, targeted knockdown of CUL5 substantially prolonged the half-life of ORF9b in cells, with the protein level remaining stable for up to 12 h after CHX treatment (Fig. 2g and Supplementary Fig. 5e). Furthermore, in vivo ubiquitination assays demonstrated that the knockdown of CUL5 resulted in attenuated levels of ORF9b protein ubiquitination (Fig. 2h). These results provided deeper insight into the critical role of CUL5 in mediating the ubiquitination and stability of ORF9b protein.

### CUL5 modulates the immunosuppressive effects of ORF9b and functions as a host antiviral factor

**Fig. 3:**
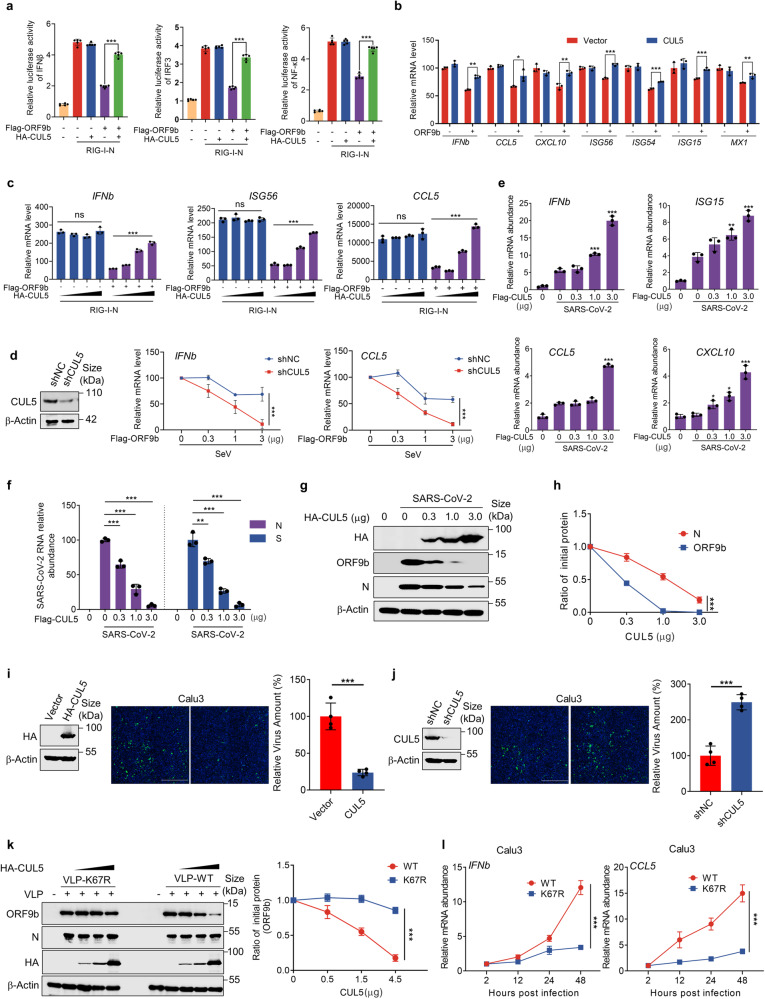
CUL5 modulates the immunosuppressive effects of ORF9b and functions as a host antiviral factor. **a** Dual luciferase reporter gene assay was performed to test the relative activity of IFNβ, IRF3 and NF-κB when co-expressing indicated proteins in HEK293T cells. The indicated firefly luciferase reporter plasmid was transfected into HEK293T cells seeded in 12-well plates. Twenty-four hours after transfection, the innate immune pathway was activated by RIG-I-N transfection for 24 h. The Samples were collected to measure luciferase activity. *n* = 5 per group. **b** HEK293T cells were transfected with indicated plasmids and infected with SeV (100 HAU/ml) for 12 h before collection. Indicated genes were determined by qRT-PCR. **c** HEK293T cells carrying indicated plasmids were transfected with *RIG-I-N* plasmid for 12 h. *IFNb*, *ISG56*, and *CCL5* were analyzed by qRT-PCR. **d** HEK293T cells stably expressing the shCUL5 or not were transfected with indicated plasmids. Cells were infected with SeV for 12 h. Expression of CUL5 was detected by Western blot (left). *INFb* and *CCL5* were analyzed by qRT-PCR (right). **e**–**h** Huh7 cells with the gradient overexpression of CUL5 were infected with SARS-CoV-2. Indicated antiviral genes (**e**), viral relative RNA abundance (**f**) and protein levels (**g**) were determined at 24 h after SARS-CoV-2 infection by qRT-PCR and Western blot. Quantification of ORF9b and N protein level normalized to β-actin (**h**). **i** Calu3 cells with overexpression of CUL5 or control were infected with SARS-CoV-2 for 24 h. The relative virus amount of SARS-CoV-2 in cells was detected by immunofluorescence assay with anti-N antibody. Scale bars, 2 mm. *n* = 4 per group. **j** The Calu3 cells stably expressing the shCUL5 or shNC were infected with SARS-CoV-2 for 24 h. The relative virus amount of SARS-CoV-2 in cells was detected by immunofluorescence assay with anti-N antibody. Scale bars, 2 mm. *n* = 4 per group. **k** Hela-hACE2 cells with the gradient overexpression of CUL5 were infected with SARS-CoV-2 K67R-VLP or WT-VLP for 24 h. Cells were collected and indicated protein levels were detected by Western blot. **l** Calu3 cells were infected with SARS-CoV-2 K67R-VLP or WT-VLP and collected as indicated time. The mRNA levels of *INFb* and *CCL5* were determined by qRT-PCR. Quantification was shown as mean ± s.d. Student’s *t*-test (unpaired, two-tailed) was used to compare two independent groups and a two-way ANOVA test was performed for comparisons of multiple groups. *P < 0.05; **P < 0.01; ***P < 0.001; n.s. not significant

The evidence presented suggests that CUL5 could act as a novel antiviral factor against SARS-CoV-2 by promoting the degradation of ORF9b, thereby inhibiting its function. To validate this hypothesis, we initially performed a dual luciferase reporter gene analysis. The results revealed that while CUL5 alone had no impact on the production of IFNβ, in cells expressing ORF9b, the overexpression of CUL5 effectively attenuated the inhibitory effect of ORF9b on IFNβ and NF-κB signaling pathways (Fig. 3a and Supplementary Fig. 6a). This finding was further substantiated by the analysis of mRNA levels of antiviral cytokines such as *IFNb*, *CCL5*, *CXCL10*, and several other ISGs (Fig. 3b). Also, the expression levels of antiviral cytokines *IFNb*, *ISG56*, and *CCL5* exhibited a dose-dependent increase with elevated CUL5 expression in the presence of ORF9b (Fig. 3c and Supplementary Fig. 6b). Conversely, knockdown of CUL5 in HEK293T cell line resulted in a more pronounced decrease in mRNA levels of *IFNb* and *CCL5* with increasing ORF9b expression compared to WT HEK293T cell (Fig. 3d). Collectively, these findings support the notion that CUL5 can modulate the immunosuppressive effect of ORF9b.

Moreover, when the cells with the gradient overexpression of CUL5 were infected with SARS-CoV-2, there was a notable increase in the levels of antiviral cytokines such as *IFNb*, *ISG15*, and *CCL5*, *CXCL10* (Fig. 3e). Additionally, it was observed that the protein levels of CUL5 were inversely correlated with viral RNA and protein (Fig. 3f–h), indicating that CUL5 can effectively reduce SARS-CoV-2 infection. To further examine the inhibitory effect of CUL5 on viral replication, we performed an immunofluorescence assay, revealing weaker virus kinetics in cells overexpressing CUL5 compared to control Calu3 cells (Fig. 3i). In contrast, knockdown of CUL5 in Calu3 cells resulted in a significantly enhanced virus replication (Fig. 3j). Furthermore, we engineered the ORF9b-K67R mutant using the ΔORF3-E VLP system.^60^ Despite the N and ORF9b proteins sharing a single open reading frame, we successfully mutated the K67 site on the ORF9b protein to R without altering the corresponding amino acids on the N protein, achieved by specifically mutating the base at site 210 of the *N* gene from “a” to “g” (Supplementary Fig. 6c). The ORF9b protein in K67R-VLP remained stable (Fig. 3k) during the gradient overexpression of CUL5, in contrast to the wild-type VLP. Additionally, the suppression of innate immune response was stronger in K67R-VLP (Fig. 3l and Supplementary Fig. 6d). Taken together, our results demonstrate that CUL5 is a crucial antiviral factor against SARS-CoV-2 by targeting ORF9b for degrading.

### HSP90α maintains the stability of ORF9b

**Fig. 4:**
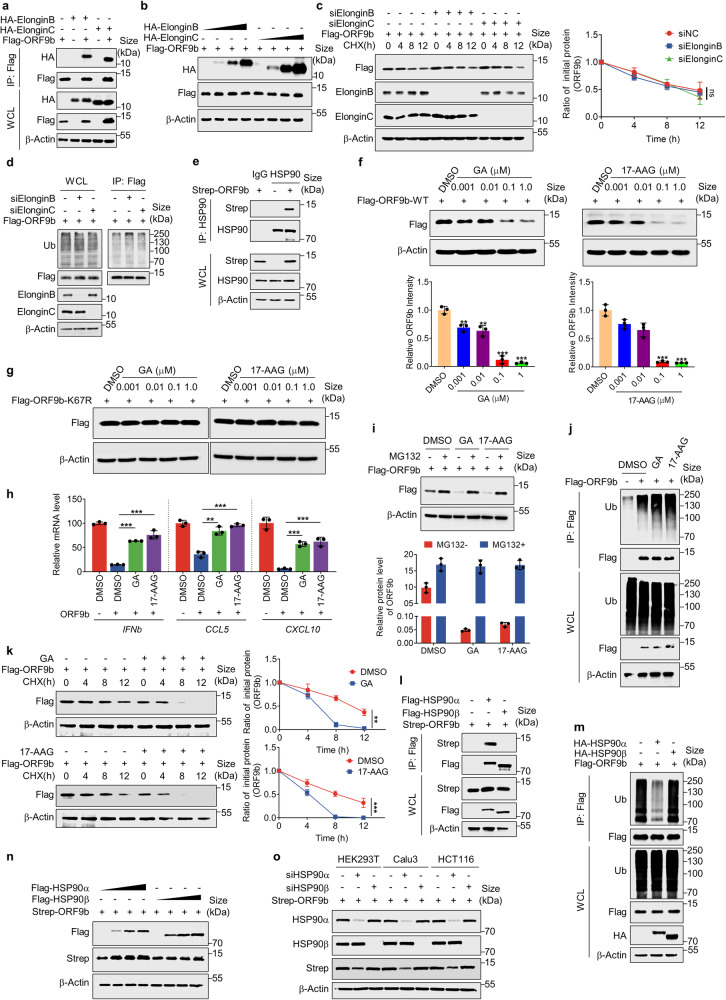
HSP90α maintains the stability of ORF9b. **a** Co-Immunoprecipitation was performed to test the interaction of SARS-CoV-2 ORF9b and ElonginB/C in HEK293T cells. **b** HEK293T cells overexpressing the ORF9b protein were transfected with ElonginB or ElonginC expressing plasmid as indicated. The ORF9b protein level was detected at 24 h after transfection. **c** HEK293T cells stably expressing the indicated siRNA were transfected with plasmids containing Flag-ORF9b, treated with CHX and collected at indicated time for Western blot. **d** In vivo ubiquitination assay of SARS-CoV-2 ORF9b when Elongin B or Elongin C was knocked down in HEK293T cells. **e** Co-Immunoprecipitation was performed to test the interaction of ORF9b and HSP90 in HEK293T cells. **f** and **g** HEK293T cells overexpressing Flag-ORF9b-WT (**f**) or Flag-ORF9b-K67R (**g**) were treated with increasing concentrations of GA or 17-AAG for 24 h and the ORF9b protein level was detected. **h** HEK293T cells expressing ORF9b were treated with DMSO, GA or 17-AAG for 12 h and then infected with SeV (100 HAU/ml). Total RNA was extracted to detect the relative levels of *IFNb*, *CCL5*, and *CXCL10*. **i** HEK293T cells overexpressing Flag-ORF9b were treated with 1.0 μM GA or 17-AAG, together treated with MG132 or not for 24 h. Cells were collected to detect the protein level of ORF9b. **j** In vivo ubiquitination assay of SARS-CoV-2 ORF9b in HEK293T cells when treated with HSP90 inhibitors (GA or 17-AAG) or not. **k** Half-life analyses of SARS-CoV-2 ORF9b when treated with GA or 17-AAG. **l** Co-Immunoprecipitation was performed to test the interaction of SARS-CoV-2 ORF9b and HSP90α/β in HEK293T cells. **m** In vivo ubiquitination assay of SARS-CoV-2 ORF9b when overexpressing HSP90α or HSP90β in HEK293T cells. **n** The ORF9b protein level was detected in HEK293T cells transfected with indicated plasmids. **o** The HEK293T, Calu3 and HCT116 cells expressing ORF9b were transfected with indicated siRNA for 36 h and collected to detect the HSP90α, HSP90β, and ORF9b protein levels. Quantification was shown as mean±s.d. *n* = 3 independent experiments. Student’s *t*-test (unpaired, two-tailed) was used to compare two independent groups, and a two-way ANOVA test was performed for comparisons of multiple groups. *P < 0.05; **P < 0.01; ***P < 0.001

Although Elongin B/C are commonly regarded as the adaptor proteins of the CRL5 E3 complex,^61^ they may act similarly on the degradation of ORF9b. While Elongin B/C did indeed co-precipitate with ORF9b from lysates of transfected HEK293T cells (Fig. 4a), the gradient overexpression of Elongin B/C proteins did not affect the protein level of ORF9b (Fig. 4b). Likewise, both overexpression and knockdown of Elongin B/C had no discernible effect on the stability of ORF9b (Fig. 4c and Supplementary Fig. 7a). Additionally, the ubiquitination level of ORF9b remained unchanged even when Elongin B/C was depleted using siRNAs (Fig. 4d). Thus, it appears that CUL5-mediated ORF9b degradation does not adhere to the classical mechanism of Elongin B/C binding to substrate proteins.

For CUL5-mediated degradation of substrate proteins, 37 substrate receptors have been identified, mostly dominated by the SOCS, ASB and SPSB families.^62^ However, while these substrate receptors typically rely on Elongin B/C for binding to CUL5, various others, such as HSP90, also play a significant role in the CUL5-mediated ubiquitination of substrate proteins. HSP90 has been reported as a CUL5 substrate receptor but functions in contrast to CUL5 by enhancing the stability of substrate protein.^36,37^ When cells were treated with HSP90 inhibitors such as GA and 17-AAG,^63,64^ the substrate proteins could be promoted to degradation induced by CUL5 via the ubiquitin-proteasome pathway. In the host-interacting protein profiling of ORF9b, abundant peptides of HSP90 were identified (Supplementary Fig. 7b), suggesting that HSP90 and ORF9b could bind to each other in host cells. Indeed, co-immunoprecipitation revealed an interaction between HSP90 and the ORF9b protein (Fig. 4e). We treated HEK293T, Calu3, and HCT116 cells overexpressing ORF9b with increasing concentrations of GA or 17-AAG, and the protein levels of ORF9b gradually decreased (Fig. 4f and Supplementary Fig. 7c, d). When cells overexpressing the ubiquitination-resistant mutant ORF9b-K67R were treated with gradually increasing concentrations of GA and 17-AAG, we found that the protein levels of ORF9b-K67R were unaffected by the HSP90 inhibitors (Fig. 4g and Supplementary Fig. 7e, f). Since ORF9b depends on its protein expression to inhibit the host’s innate immune signaling pathways, the degradation of ORF9b protein could diminish its ability to fight against the host’s innate immunity. Therefore, we treated cells overexpressing ORF9b with 1.0 μM GA or 17-AAG and activated the innate immune pathway with SeV infection for 12 h. The results showed that GA and 17-AAG effectively attenuated the inhibitory effect of ORF9b protein on antiviral cytokines and enhanced the host innate immunity (Fig. 4h). When cells overexpressing ORF9b were treated with 1.0 μM GA or 17-AAG, although drug treatment increased the degradation of ORF9b protein, the protein level of ORF9b recovered to the same level as the control when treated with MG132, suggesting that GA or 17-AAG-induced degradation of ORF9b protein occurred through the ubiquitin-proteasome pathway (Fig. 4i). In vivo ubiquitination assays also showed that the ubiquitination level of ORF9b protein was significantly enhanced when cells were treated with 1.0 μM GA or 17-AAG (Fig. 4j). The half-life further showed that ORF9b protein degraded faster with the treatment of GA or 17-AAG (Fig. 4k). Taken together, HSP90 has the opposite function to CUL5 and can enhance the stability of ORF9b.

HSP90 is a stress protein in the heat shock protein family that is highly conserved and present in various organisms. It is divided into five subfamily members, namely HSP90α, HSP90β, HSP90C, TRAP, and HtpG.^65,66^ Our mass spectrometry results identified many peptides of HSP90α and HSP90β. However, co-immunoprecipitation experiments showed that ORF9b interacted with HSP90α but not HSP90β in the cells (Fig. 4l). The in vivo ubiquitination assay showed that the overexpression of HSP90α, but not HSP90β, significantly reduced the ubiquitination level of ORF9b (Fig. 4m). Moreover, gradient overexpression of HSP90α enhanced the protein level of ORF9b compared to HSP90β (Fig. 4n). The analysis of ORF9b protein half-life revealed that HSP90α overexpression enhanced its stability, whereas HSP90β did not (Supplementary Fig. 7g, h). Knockdown of HSP90α but not HSP90β led to a decrease in ORF9b protein levels in HEK293T, Calu3, and HCT116 cells (Fig. 4o). These results demonstrated that HSP90α plays a regulatory role in the stability of ORF9b protein, whereas HSP90β does not.

### TOM70 serves as a substrate receptor of CUL5-based E3 ligase for ORF9b

**Fig. 5:**
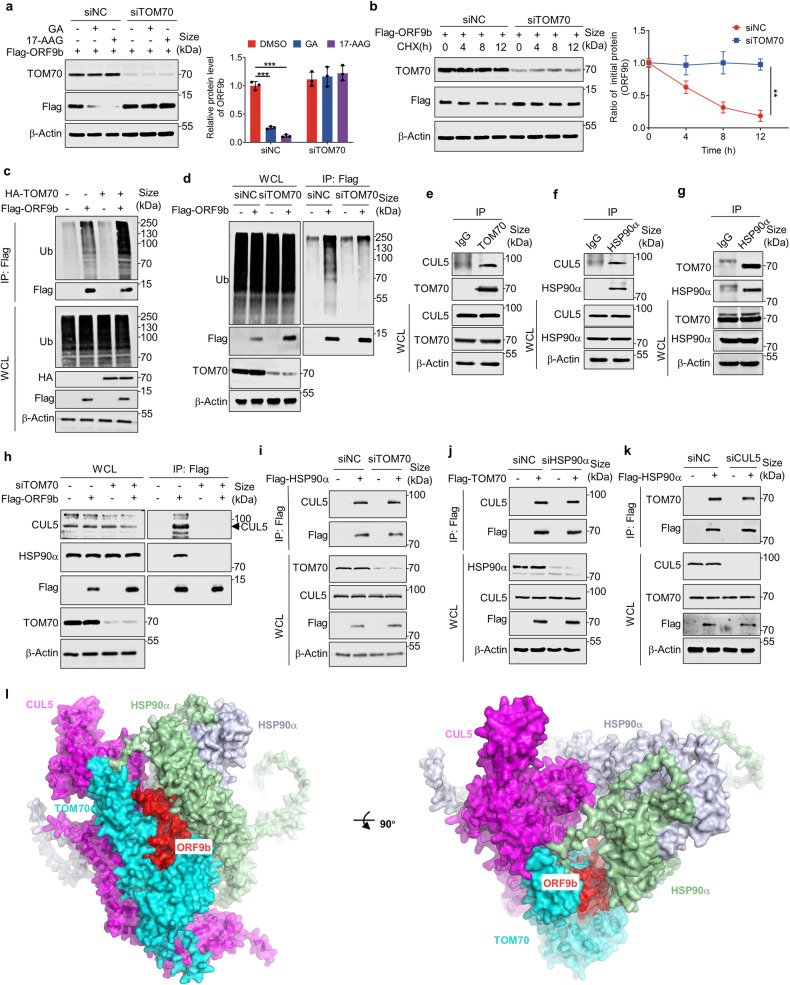
TOM70 serves as a substrate receptor of CUL5-based E3 ligase for ORF9b. **a** HEK293T cells expressing siNC or siTOM70 were transfected with plasmids containing *ORF9b* and treated with DMSO, GA or 17-AAG for 24 h. The protein levels of ORF9b were detected by Western blot. **b** Half-life analyses of SARS-CoV-2 ORF9b when knocking down TOM70 or not in HEK293T cells. **c** In vivo ubiquitination assay of SARS-CoV-2 ORF9b when overexpressing TOM70 or not in HEK293T cells. **d** In vivo ubiquitination assay of SARS-CoV-2 ORF9b when knocking down TOM70 or not in HEK293T cells. **e**–**g** Co-Immunoprecipitation was performed to test the interaction among TOM70, CUL5 and HSP90α in HEK293T cells. **h** The interactions between SARS-CoV-2 ORF9b and CUL5, or ORF9b and HSP90α were tested in HEK293T cells when siTOM70 was transfected or not. **i**–**k** HEK293T cells expressing indicated siRNAs were transfected with plasmids expressing Flag-tagged proteins. The whole cell lysates and precipitated proteins were analyzed by immunoblotting with indicated antibodies. **l** A structural model of the ORF9b–TOM70–CUL5–HSP90α complex is presented in two orthogonal views. The complex includes two copies of HSP90α (light green and blue-white) and one copy each of ORF9b (red), CUL5 (magenta), and TOM70 (cyan). The model was generated using AlphaFold v2.3.2. Quantification was shown as mean ± s.d. *n* = 3 independent experiments. Student’s *t*-test (unpaired, two-tailed) was used to compare two independent groups, and a two-way ANOVA test was performed for comparisons of multiple groups. **P < 0.01; ***P < 0.001

It has been reported that ORF9b protein can interact with the C-terminal domain of host TOM70, affecting its N-terminal binding to HSP90.^12,67,68^ We found that the overexpression of TOM70 decreased the ORF9b protein level in a gradient manner and accelerated its degradation rate (Supplementary Fig. 8a, b). Knockdown of TOM70 by siRNA prevented the promotion of ORF9b degradation by HSP90 inhibitor and enhanced the stability of ORF9b protein (Fig. 5a, b). Additionally, TOM70 affected the formation of polyubiquitin chains on ORF9b (Fig. 5c, d). These results demonstrated that TOM70 played a crucial role in the ubiquitination and proteasome-mediated degradation of ORF9b protein.

Delving deeper, we aimed to investigate the interplay among TOM70, CUL5 and HSP90α proteins. It has been established that the HSP90 protein can directly interact with TOM70. Through co-immunoprecipitation experiments, we observed interactions among CUL5, TOM70, and HSP90α within cells (Fig. 5e–g). Interestingly, knocking down TOM70 led to the loss of interactions between ORF9b and HSP90α, as well as between ORF9b and CUL5 (Fig. 5h), suggesting that HSP90α does not act as a substrate receptor in the complex. However, knocking down any one of the proteins within the CUL5-TOM70-HSP90α complex did not affect the interaction between the other two, implying that these three proteins bind to each other in pairs (Fig. 5i–k). Furthermore, the interaction between the two remaining proteins remained unaffected when CUL5, TOM70, and HSP90 were individually overexpressed in a gradient, indicating the absence of competition between them (Supplementary Fig. 8c). These findings suggest that TOM70 might serve as a substrate receptor in the HSP90α-CUL5 regulation of ORF9b stability. Indeed, a phosphomimetic mutant ORF9b-S53E, with drastically reduced binding to TOM70, exhibited a longer half-life compared to ORF9b-WT (Supplementary Fig. 8d). Several studies have reported that ORF9b exists in two forms: monomer and dimer. Among these, the monomeric form of ORF9b can bind to TOM70, while the dimeric form does not.^12,54^ To explore the correlation between the conformation of ORF9b and its stability. we lysed cells overexpressing ORF9b with a non-reducing buffer (without SDS and DTT) and examined the protein levels of ORF9b monomer and dimer in the presence or absence of overexpressed HSP90α. Our results demonstrate that HSP90α specifically enhances the protein level of ORF9b in its monomeric form with no effect on dimerization. Therefore, we hypothesize that only the monomeric form of ORF9b is susceptible to degradation, whereas the dimeric form remains stable (Supplementary Fig. 8e). Based on the aforementioned exploration, we propose a model in which the CUL5-TOM70-HSP90α complex collaboratively regulates the stability of ORF9b protein. In this model, TOM70 acts as a substrate receptor, facilitating the binding of HSP90α and CUL5 to ORF9b, with HSP90α enhancing its stability while CUL5 inducing its ubiquitination and subsequent degradation.

The amino acid sequences and structures of SARS-CoV and SARS-CoV-2 ORF9b proteins share 73% homology. In cell lysates, TOM70, HSP90α and CUL5 were also co-precipitated with SARS-CoV ORF9b, and GA and 17-AAG treatment had no effect on their interactions (Supplementary Fig. 9a). Similarly, the SARS-CoV-2 ORF9b ubiquitination-resistant mutant K67R could also interact with these proteins, unaffected by HSP90 inhibitors (Supplementary Fig. 9b). Notably, HSP90 inhibitors target the GA domain of HSP90 to inhibit the ATPase activity, but do not interfere with the interactions within the CUL5-TOM70-HSP90α complex (Supplementary Fig. 9c, d). We have documented that the half-life of SARS-CoV ORF9b and SARS-CoV-2 ORF9b-K67R were significantly extended compared to SARS-CoV-2 ORF9b-WT, suggesting that ubiquitination of the K67 site is essential for the regulation of SARS-CoV-2 ORF9b stability by the CUL5-TOM70-HSP90α complex.

To gain further insight into the organization of this complex, we have generated a structural model of the ORF9b-CUL5-TOM70-HSP90α complex using AlphaFold2.^69^ This quaternary complex comprises two copies of HSP90α and one copy each of ORF9b, CUL5, and TOM70 (Fig. 5l). According to this model, ORF9b binds to the C-terminal domain of TOM70, which is highly consistent with previous structural studies (PDB: 7DHG, PubMed ID: 33990585).^12^ Furthermore, the ORF9b-bound TOM70 and CUL5 both bind to the HSP90α dimer from the same side while interacting with each other. This predicted model aligns well with our experimental data, suggesting the important role of TOM70 in mediating the binding of ORF9b to CUL5 and HSP90α and that CUL5, TOM70, and HSP90α can form pairs in their interactions. However, we acknowledge the necessity for further structural analysis, such as X-ray crystallography or cryo-electron microscopy, to validate these findings.

### HSP90 inhibitors restrict the replication of SARS-CoV-2 by promoting ORF9b degradation

**Fig. 6:**
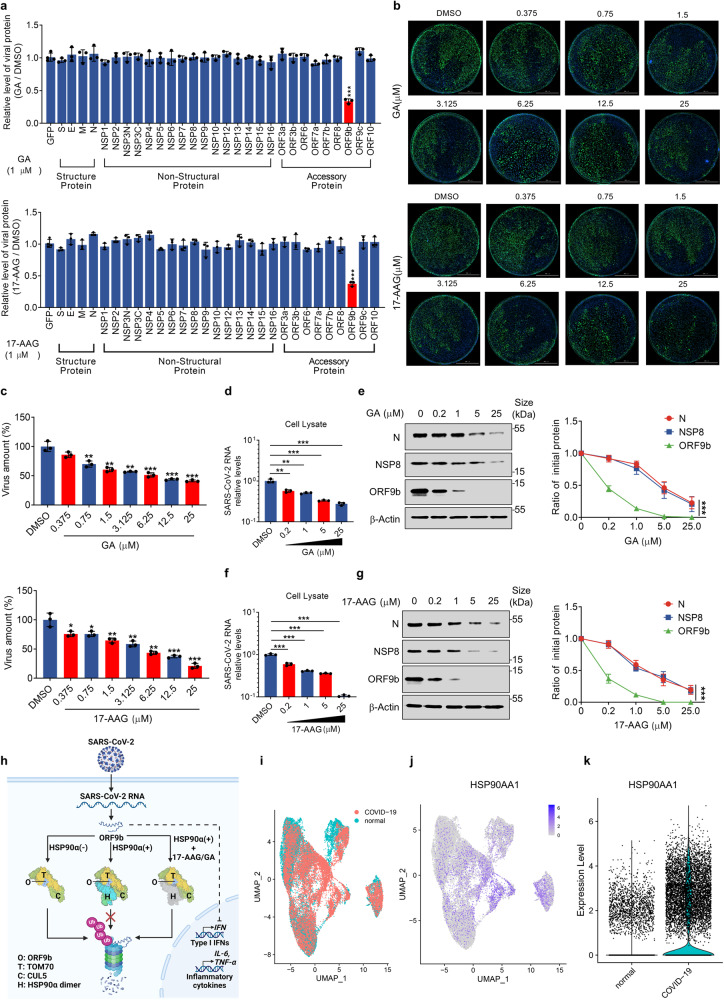
HSP90 inhibitors restrict the replication of SARS-CoV-2 by promoting ORF9b degradation. **a** The plasmids containing Strep-tagged SARS-CoV-2 viral genes were transfected into HEK293T cells and treated with indicated concentrations of GA or 17-AAG for 24 h. The cells were lysed to detect the viral protein levels and quantification of different viral proteins was normalized to β-actin. **b** and **c** Calu3 cells treated with indicated increasing concentrations of GA (**b**-up), 17-AAG (**b**-down) or DMSO were inoculated with SARS-CoV-2 at MOI = 1 for 24 h. Quantification of relative virus amount was measured by immunofluorescence (**c**). **d**–**g** Calu3 cells infected with SARS-CoV-2 at a MOI = 1 were treated with indicated concentrations of GA (**d** and **e**) or 17-AAG (**f** and **g**) for 24 h. Relative mRNA (left) and protein levels (right) were detected by qRT-PCR and Western blot as indicated. **h** A schematic diagram illustrates the regulation of the TOM70–CUL5–HSP90α complex on ORF9b. The interaction model of the complex was based on a predicted model generated using AlphaFold, as shown in Fig. 5l. Different components are represented by distinct colors: ORF9b is depicted in sky blue, TOM70 in yellow, CUL5 in yellow-green, HSP90α monomer 1 in cyan, HSP90α monomer 2 in aquamarine, and the nonfunctional HSP90α dimer in gray. This diagram demonstrates how, upon SARS-CoV-2 entry, host cells counteract viral immune evasion by targeting ORF9b for ubiquitination and subsequent degradation. In the absence of HSP90α, TOM70 and CUL5 mediate ORF9b degradation via the ubiquitin-proteasome pathway (left). Conversely, in the presence of HSP90α, ORF9b is shielded from degradation (middle). However, HSP90 inhibitors such as 17-AAG/GA deactivate HSP90αα, leading to the degradation of ORF9b (right). **i**–**k** A dataset was obtained from GEO with accession number GSE171524 to analyze the difference in RNA levels of *HSP90AA1* between lung epithelial cells of COVID-19 patients and healthy control. Group origins of cells (**i**) and RNA levels of *HSP90AA1* in single cells (**j**) were shown with a UMAP plot. Violin plot (**k**) was performed to show the difference in *HSP90AA1* RNA levels between lung epithelial cells of COVID-19 patients and healthy control. Quantification was shown as mean ± s.d. *n* = 3 independent experiments. Student’s *t*-test (unpaired, two-tailed) was used to compare two independent groups, and a two-way ANOVA test was performed for comparisons of multiple groups. *P < 0.05; **P < 0.01; ***P < 0.001

Our investigations confirm that HSP90 enhances the stability of ORF9b protein, and treatment with the HSP90 inhibitors GA or 17-AAG promotes CUL5-mediated degradation of ORF9b protein via the UPS. The genome of SARS-CoV-2 encodes a total of 29 viral proteins. To investigate whether HSP90 specifically targets the ORF9b protein, all viral proteins were overexpressed in HEK293T cells and treated with HSP90 inhibitors GA or 17-AAG for 24 h. It is worth noting that NSP3 was divided into two segments, NSP3-N and NSP3-C, due to its large size, while NSP11 was not included in the screening due to its extremely small molecular weight of less than 2 kDa. The results showed that the HSP90 inhibitor only attenuated the protein level of ORF9b without affecting the expression of other proteins (Fig. 6a and Supplementary Fig. 10a, b).

Furthermore, we sought to investigate whether HSP90 inhibitors could inhibit the replication ability of SARS-CoV-2. Initially, we tested the toxicity of GA and 17-AAG on Calu3 cells, and the results showed that concentrations up to 50 μM were less toxic to cells (Supplementary Fig. 10c). Next, we infected Calu3 cells with wild-type SARS-CoV-2 at an MOI = 0.1 and treated the cells with GA or 17-AAG in increments up to 25 μM. After 24 h, we detected the intracellular replication level of the virus by immunofluorescent assay (IFA) and observed a gradual decrease in viral load with increasing GA concentrations (Fig. 6b, c). Using the *N* gene of SARS-CoV-2 as the detection index, we analyzed the relative level of SARS-CoV-2 in the infected cells by qPCR, and the results were consistent with those of the IFA assay (Fig. 6d). Additionally, we examined the levels of viral proteins N, NSP8, and ORF9b in the infected cells. Our results indicated that HSP90 inhibitors did not affect the stability of N and NSP8 and that the reduction in their levels was associated with a diminution of the virus. Interestingly, ORF9b was more rapidly degraded than N and NSP8 (Fig. 6e). Notably, 17-AAG had the same effect on SARS-CoV-2 as GA (Fig. 6f, g). Furthermore, our work revealed that intracellular overexpression of HSP90α can increase ORF9b levels, while the HSP90α-induced IFNβ pathway was inhibited relative to cells that did not express ORF9b (Supplementary Fig. 10d, e). These results corroborated the mechanism we elucidated that HSP90 inhibitors could induce more ubiquitinated degradation of ORF9b protein. Taken together, our results provide a pattern diagram reflecting the regulation of ORF9b stability and innate immune responses by the CUL5-TOM70-HSP90α complex (Fig. 6h).

The aforementioned data have demonstrated that HSP90α enhances the stability of ORF9b protein, which is crucial for immune evasion of SARS-CoV-2. It has been reported that the RNA levels of *HSP90AA1* and the amount of SARS-CoV-2 virus RNA were positively correlated in SARS-CoV-2-infected H1299 and Calu3 cells.^70^ To further verify the correlation between *HSP90AA1* and SARS-CoV-2, we collected publicly available single-cell sequencing data from lung epithelial cells of COVID-19 patients, completed the clustering and identification of cellular taxa (Supplementary Fig. 11a, b). Then we analyzed the expression of HSP90α in lung epithelial cells. The results showed that the level of *HSP90AA1* in the lung epithelial cells of the COVID-19 patients was significantly higher than that in the control group (Fig. 6i–k and Supplementary Fig. 11c). Meanwhile, it has long been known that patients with other diseases, especially respiratory and gastrointestinal systemic diseases, are more likely to develop severe disease after SARS-CoV-2 infection. In this context, we wanted to explore further whether HSP90α plays an important role in this phenomenon. To achieve this, we analyzed publicly available single-cell sequencing databases related to chronic obstructive pulmonary disease (COPD), Crohn’s disease, and colorectal cancer (CRC). Remarkably, single-cell sequencing of lung tissues from COPD patients and healthy individuals showed that HSP90α expression was higher in the epithelial cells of COPD patients than in healthy individuals (Supplementary Fig. 12a). Moreover, HSP90α expression was higher in the goblet cells of Crohn’s patients compared to healthy controls (Supplementary Fig. 12b). When comparing cancer and normal tissues from CRC patients, the HSP90α expression was relatively higher in the epithelial and goblet cells of cancer tissues (Supplementary Fig. 12c). Interestingly, the epithelial cells and goblet cells of the lung and intestinal tract are the main target cells of SARS-CoV-2 infection in COVID-19 patients. Further analysis using the GEPIA database and IHC assay confirmed that the expression of HSP90α was significantly higher in cancer tissues than in normal tissues of colon adenocarcinoma (COAD) and rectum adenocarcinoma (READ) patients (Supplementary Fig. 12d–f). These findings provide insights into why patients with these diseases are more vulnerable to SARS-CoV-2.

### Inhibition of HSP90 attenuates the virulence of SARS-CoV-2 in a mouse infection model

**Fig. 7:**
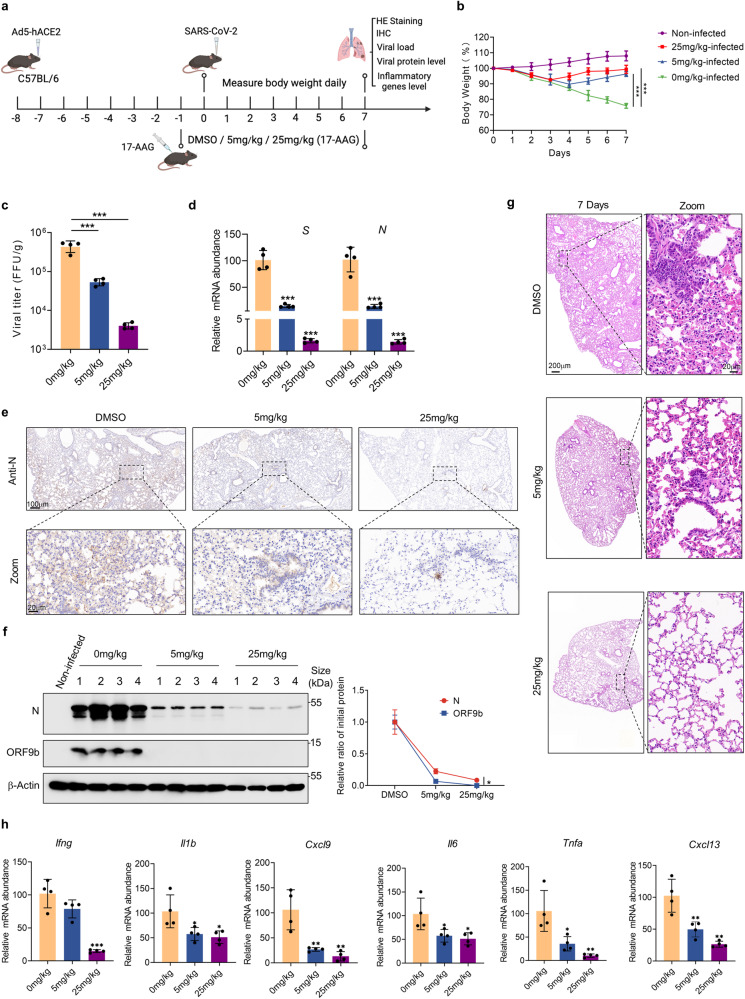
Inhibition of HSP90 attenuates the virulence of SARS-CoV-2 in the infection mouse model. **a** C57BL/6-hACE2 mice were intraperitoneally injected with 0, 5, or 25 mg/kg 17-AAG for 8 consecutive days. On the second day of drug treatment, the mice were nasally infected with SARS-CoV-2. **b** Daily recordings of mouse body weights commenced at the time of infection. **c** Lung tissues were collected seven days post-infection, lysed, and subjected to viral titer analysis using FFA. **d** Total RNA was extracted from mouse lung tissues, and relative levels of SARS-CoV-2 *S* and *N* genes were detected. **e** The levels of SARS-CoV-2 N protein in the lungs of infected mice treated with 17-AAG or DMSO were assessed by Immunohistochemistry with the anti-SARS-CoV-2 N antibody. **f** SARS-CoV-2 N proteins and ORF9b proteins in mouse lung tissues were detected by Western blot. **g** H&E staining of mouse lung tissues was performed to visualize the extent of inflammatory infiltration. **h** Relative RNA abundance of indicated cytokines or inflammatory factors in mouse lung tissues was measured by qRT-PCR. Quantification was shown as mean ± s.d. *n* = 4 independent experiments. Student’s *t*-test (unpaired, two-tailed) was used to compare two independent groups, and a two-way ANOVA test was performed for comparisons of multiple groups. *P < 0.05; **P < 0.01; ***P < 0.001

To further validate the antiviral effects of HSP90 inhibitors in vivo, we performed the analysis using a mouse infection model constructed with Ad5-hACE2. C57BL/6 mice at 6–8 weeks were selected and pre-infected with Ad5-hACE2 nasal drops one week in advance. After 7 days, we administered 17-AAG (at a dose of 5 and 25 mg/kg) or dimethyl sulfoxide (DMSO) continuously daily via intraperitoneal injection. One day after the first administration, we intranasally infected mice with 5 × 10^4^ PFU WT SARS-CoV-2 and monitored their daily weight changes (Fig. 7a). In the non-infected group, the mice showed a slight increase in body weight. In contrast, the weight of mice in the infected and untreated group decreased daily by ~25% 7 days after infection. The body weights of the mice in the infected drug-treated groups all displayed a pattern of decline followed by recovery. The mice in the 25 mg/kg group experienced a maximum body weight loss of about 7% and began to regain weight on the 4th day after infection, eventually returning to near pre-infection levels. Meanwhile, the mice in the 5 mg/kg group began to recover their body weights on the 5th day and showed a maximum drop of about 10% (Fig. 7b).

On the 7th day after infection, all mice were dissected, and lung tissues were taken for further analysis. Viral titers and RNA abundance showed that drug treatment significantly attenuated viral load in the lungs in a dose-dependent manner (Fig. 7c and d). Furthermore, immunohistochemistry revealed a remarkable reduction in virus distribution in the lungs of HSP90 inhibitor-treated mice (Fig. 7e). Compared to the N protein, the proportion of ORF9b protein decreased more rapidly with increasing drug dose, suggesting that ORF9b is more readily degraded when HSP90 activity is inhibited (Fig. 7f). H&E (Hematoxylin and Eosin) staining showed reduced inflammatory infiltration in the lungs of drug-treated mice (Fig. 7g), accompanied by decreased abundance of several inflammatory factors compared to the untreated group (Fig. 7h). Collectively, HSP90 inhibitors markedly inhibited viral replication and virulence in vivo.

## Discussion

Recent studies have highlighted the crucial role of SARS-CoV-2 ORF9b in immune evasion,^55^ while the precise regulatory mechanisms remain unclear. During the preparation of manuscript, Gao, W. et al. reported that SARS-CoV-2 ORF9b is degraded via the UPS,^13^ consistent with our finding. Additionally, we found that the half-life of ORF9b in SARS-CoV-2 was shorter than in SARS-CoV, which may partly account for the lower lethality of SARS-CoV-2 despite its greater transmissibility than SARS-CoV. Although the virus constantly mutates to produce variants such as Delta and Omicron strains, the K67 ubiquitination site of ORF9b protein is conserved, implying a common host antiviral mechanism and inspiring us to target the ORF9b degradation as a potential strategy to develop effective anti-SARS-CoV-2 drugs.

After conducting affinity purification and mass spectrometry, we successfully identified CUL5, a scaffold protein in the CRL5 complex, responsible for the degradation of ORF9b. Given that there are 37 substrate receptors of CRL5, it is crucial to identify which one is responsible for the degradation of ORF9b. Using HSP90 inhibitors, HSP90 overexpression, and siRNA knockdown, we identified that HSP90α specifically targets ORF9b, promoting viral replication by enhancing its stability. Analysis of available single-cell databases shown that HSP90α was highly expressed in the epithelial or goblet cells of patients with respiratory and gastrointestinal diseases, which may partly explains why such patients are more susceptible to developing severe disease after SARS-CoV-2 infection.^71,72^ Although a recent report identified N, M, and three more proteins from SARS-CoV-2 as potential downstream targets when treated with an HSP90 inhibitor,^45^ our data strongly supports that ORF9b is likely the main target. In short, our findings provide a new theoretical foundation for treating SARS-CoV-2 with HSP90 inhibitors.

It has been reported that SARS-CoV-2 ORF9b protein can bind to the C-terminus of TOM70, which attenuates the interaction between TOM70 N-terminus and HSP90. This disruption ultimately affects the HSP90-mediated phosphorylation of IRF3 and inhibits the innate immune pathway.^11,73^ Our study revealed that TOM70 also affects the ubiquitination and stability of ORF9b. Knocking down TOM70 weakens the interaction of ORF9b with HSP90 and CUL5 while significantly enhancing ORF9b stability. Phosphorylation at the S53 site hampers the interaction between ORF9b and TOM70, and the half-life of phosphorylation-resistant mutant S53E is significantly higher than that of WT, further supporting TOM70 as the bridge of ORF9b to ubiquitin-proteasome degradation. Although HSP90 is believed to be the substrate receptor in CRL5,^36^ our study of the ORF9b stability mediated by the CUL5-TOM70-HSP90α complex supports that TOM70 seems to function more like a substrate receptor and plays a critical role in linking ORF9b to HSP90-CUL5 complex. The structure of the complex predicted by AlphaFold also provides some support for our hypothesis. However, whether this phenomenon is universal and can overturn the previous role of HSP90 as a substrate receptor for CUL5 warrants further investigation.

The fight between the host and virus is a universal phenomenon, wherein the host largely relies on immune responses to combat the virus, while the virus manipulates the host pathway and evades the immune responses. Our study has uncovered a novel mechanism highlighting the intense battle between the host and SARS-CoV-2. It is widely recognized that ORF9b binding to TOM70 impedes the downstream activation of IFN and NF-κB pathway. However, the host protein CUL5 also binds to TOM70, triggering ORF9b ubiquitination and degradation, subsequently decreasing the ORF9b effect and more likely acting as a host antiviral factor. Furthermore, SARS-CoV-2 can take advantage of the role of HSP90α and stabilize its accessory protein ORF9b, benefiting viral replication and virulence. To our knowledge, this is the first report detailing such intricate regulation by a viral accessory protein, SARS-CoV-2 ORF9b. The comprehensive mechanism we have described offers a potential new target and strategy for the clinical treatment of SARS-CoV-2.

Some intriguing observations warrant further exploration. It is generally believed that targeting viral proteins would offer a more direct and effective approach, largely due to the common feature of the absence of host homology in most virus proteins, thereby reducing potential side effects. However, unlike extensively studied viral targets such as PLpro, Mpro, and RdRp, ORF9b lacks enzymatic activity, posing a challenge in designing inhibitors that target active pockets like those in the aforementioned targets. While targeting host proteins, particularly multifunctional ones like HSP90, may entail more side effects, it is noteworthy that HSP90 is currently a potential target in the field of oncology, with numerous related drugs already developed and in clinical trials. Therefore, targeting HSP90 to induce ORF9b degradation presents a viable option. Moreover, employing a specific inhibitor of HSP90α, rather than HSP90 in general, could enhance the specificity of the clinical strategy. Another intriguing aspect is that TOM70 serves not only as a target for ORF9b to modulate innate immunity, as previously reported.^11^ but also act as a substrate receptor to mediate ORF9b degradation. While it has been reported that ORF9b interacts with the C-terminal domain of TOM70 and affects TOM70 binding to HSP90,^12^ our data supports that ORF9b and HSP90α bind simultaneously to TOM70. This suggests that the competitive binding between ORF9b and HSP90α may not be entirely mutually exclusive, possibly due to HSP90 possessing two binding sites to TOM70^68^ and ORF9b being a fold-switching protein.^74^ While this deepens our understanding of the regulatory complexities in this scenario, further investigation, particularly through structural analysis, is warranted to elucidate the exact mechanism.

In summary, our study reveals for the first time that the CUL5-TOM70-HSP90α complex regulates the stability of SARS-CoV-2 ORF9b protein. With this complex, TOM70 acts as a substrate receptor, HSP90 enhances the stability of ORF9b protein, while CUL5 induces the degradation of ORF9b protein through the ubiquitin-proteasome system. This intricate regulation highlights the battle between the host cells and SASR-CoV-2, providing a new target and strategy for the clinical treatment of the virus.

## Materials and methods

### Ethics statement

The collection of tissues from hospitalized patients was approved by the ethics committee of the School of Life Sciences, Central South University (No. 2023-1-30). All research participants signed the informed consent. COAD and para-cancerous tissues from 5 COAD patients (29–50 years old). READ tissues and para-cancerous tissues from 5 READ patients (30–62 years old).

The animal experiments involving authentic SARS-CoV-2 were conducted in the ABSL-3 Laboratory of Shenzhen Third People’s Hospital. The animal experimental procedures used in the study were approved by the Institutional Animal Care and Use Committee of Shenzhen Third People’s Hospital (No. 2023-015).

### Cell culture

HEK293T, Vero E6 and BEAS-2B were cultured in Dulbecco’s modified Eagle’s medium (DMEM, Hyclone). Calu3 was cultured in MEM medium (Hyclone). The other cell lines, including A549, HCT116, HT29, NCM460, MCF7, Huh7 and HeLa, were cultured in RPMI medium (Hyclone). All the cell lines were supplemented with 10% fetal bovine serum (FBS, ZATA), penicillin (100 U/ml), and streptomycin (100 μg/ml) and maintained at 37 °C in a humidified atmosphere of 5% CO_2_.

### Viruses

Sendai virus (SeV) was kindly gifted from Hao Feng (Hunan Normal University). The SARS-CoV-2 WT strain was isolated from nasopharyngeal aspirate specimens sourced from COVID-19 patients at Shenzhen Third People’s Hospital and propagated in Vero E6 cells.

### Plasmids, siRNAs, and reagents

The expressing plasmids containing SARS-CoV-2 viral genes and the pCAG-ORF9b-Flag plasmd for SARS-CoV-2 *ORF9b* were purchased from Tsingke. The SARS-CoV-2 ORF9b-3R, K4R, K59R, K67R, R4K, R59K, and R67K were cloned into pCAGGS vector with Flag-tag by ourselves. CUL5 and Ub expressing plasmids were gifts from Lingqiang Zhang’s lab. All other genes used in this work were reverse transcribed from cellular RNA and cloned into the pCAGGS plasmid by ourselves. Additionally, SARS-CoV-2 *ORF9b* was individually inserted into plasmids with CMV, CAG, and TRE promoters by ourselves as well.

All siRNAs were synthesized by RIBOBIO and the sense strand sequences are blown.

ElonginB: 5’-UGAACAAGCCGUGCAGUGA-3’

ElonginC: 5’-AAACCAAUGAGGUCAAUUU-3’

HSP90: 5’-ACAAGAAGAAGAAGAAGAA-3’

HSP90α: 5’-GGAAAGAGCUGCAUAUUAATT-3’

HSP90β: 5’-GGAGAUUUUCCUUCGGGAGTT-3’

TOM70: 5’-GGCAUUAACAGAUCAACA-3’

Geldanamycin (HY-15230) and 17-AAG (HY10211) were purchased from MedChemExpress.

### Animal experiments

C57BL/6 mice aged 6–8 weeks were intranasally transduced with 2.5 × 10^8^ FFU Ad5-hACE2. The mice expressing hACE2 were then treated with either 5 mg/kg/day or 25 mg/kg/day of 17-AAG or DMSO diluted in 60% propylene glycol and 30% physiological saline until they were sacrificed at the end of the experiment. One day after the drug treatment, mice were anesthetized and intranasally inoculated with 5 × 10^4^ FFU WT SARS-CoV-2 strain. The body weights of the mice were monitored daily, and their lungs were harvested for the analysis of the viral titer, mRNA abundance, Western blotting, Immunofluorescence and H&E staining. All the animal experiments related to SARS-CoV-2 were conducted in the ABSL-3 Laboratory of Shenzhen Third People’s Hospital.

### Cell transfections, immunoprecipitation and immunoblotting

Cells were transfected with various plasmids using Lipofectamine 3000 (Invitrogen) reagent according to the manufacturer’s protocol. Forty-eight hours later, cells were harvested and lysed with NP40 buffer (50 mM Tris–HCl [pH 7.4], 150 mM NaCl, 1% NP-40, 5 mM EDTA, 5% glycerol) supplemented with a protease inhibitor cocktail (Bimake). Whole-cell lysates were sonicated and centrifuged at 12,000 rpm for 15 min. The supernatant was harvested and incubated with anti-Flag agarose beads at 4 °C for 4 h. The agarose beads were washed extensively, and samples were eluted by boiling at 100 °C for 10 min with protein loading buffer. Both lysates and immunoprecipitation were examined using the indicated primary antibodies, followed by detection with the related secondary antibodies and the Immobilon Western Chemiluminescent HRP Substrate (Millipore). Antibodies used in immunoblotting: anti-FLAG (14793, CST), anti-HA (5017, CST), anti-Ubiquitin (LSS-VU-0101-0050, LifeSensors), anti-Strep (AE066, Abclonal), anti-CUL5 (A5369, Abclonal), anti-HSP90 (ab109248, Abcam), anti-HSP90α (A5006, Abclonal), anti-HSP90β (A19574, Abclonal), anti-TOM70 (A4349, Abclonal), anti-ElonginB (A5362, Abclonal), anti-ElonginC (A12515, Abclonal), anti-SARS-CoV-2-N (A20142, Abclonal), SARS-CoV-2-NSP8 (A20202, Abclonal), anti-SARS-CoV-2-ORF9b (60951, CST), anti-β-actin (BM21023, OMICSAB) and anti-GAPDH (BM14504, OMICSAB).

### Protein purification and mass spectrometry

To identify the interacting proteins and ubiquitination sites of ORF9b, HEK293T cells were transfected with plasmids containing Flag-ORF9b. Transfected cells were harvested at 48 h post-transfection, and the ORF9b proteins were purified with Flag-conjugated agarose beads (Sigma) from whole cell lysates. The proteins on the beads were eluted using 0.2 M glycine–HCl buffer (pH 3.0) and neutralized with 1.0 M Tris–HCl buffer (pH 9.0). Subsequently, the proteins were digested by trypsin, and each fraction was injected for nanoLC-MS/MS analysis. MS spectrometry was searched using Proteome Discoverer engine (version 1.4) against a non-redundant International Protein Index sequence database v3.85. For protein identification, the following parameters were used. Peptide mass tolerance = 20 ppm, MS/MS tolerance = 0.1 Da, Missed cleavage = 2. The LC–MS/MS analysis and data analysis were performed by SHANGHAI APPLIED PROTEIN TECHNOLOGY.

### CHX chase assay (protein half-life assay)

Plasmids encoding *ORF9b* WT were transfected into target cells when these cells in 6 cm dishes reached about 90% confluence. Twelve hours after transfection, the cells were evenly divided into 12-well plates. Twenty hours later, the cells were treated with CHX (Sigma, 50 μg/ml) for the indicated durations before collection. ORF9b mutants were used in transfection as indicated in individual experiments. The cells stably expressing the indicated shRNAs were also transfected into ORF9b and then treated with CHX. For the proteasome inhibitor, MG132 (Sigma, 20 μM) was added to the cells at the same time as CHX.

### In vivo ORF9b ubiquitination assay

For the in vivo ORF9b ubiquitination assay, Flag-ORF9b and HA-ubiquitin were transfected into HEK293T cells. Twenty-four hours later, the cells were treated with 20 μM of the proteasome inhibitor MG132 (Sigma) for 8 h. The cells were washed with PBS, pelleted, and lysed with NP40 lysis buffer (50 mM Tris–HCl [pH 7.4], 150 mM NaCl, 1% NP-40, 5 mM EDTA, 5% glycerol) plus 0.1% SDS, 10 mM DTT and 20 μM MG132. The lysates were centrifuged to obtain cytosolic proteins and incubated with anti-Flag agarose beads for 4 h at 4 °C. Then the beads were washed three times with PBS. The proteins were released from the beads by boiling them in a protein-loading buffer and analyzed by immunoblotting.

### Lentivirus-mediated stable cell line construction

When the HEK293T cells grew to 60%, the lentiviral packaging plasmid and shRNA were co-transfected into the cells in a certain ratio (psPAX2: pMD2.G: shRNA = 5:7:10) through Lipofectamine3000, and replaced with new DMEM medium after 6 hours of transfection. After 48 h of transfection, the culture medium was collected, centrifuged, and filtered with a 0.45 μm filter membrane to obtain the virus liquid. HEK293T cells were infected with the lentivirus in the presence of polybrene (5 μg/ml) with centrifugation at 1800 rpm for 40 mins at 30 °C. Cells were selected at 48 h post-infection and maintained in 10% FBS DMEM supplemented with puromycin (1– 2 μg/ml). The knockdown efficiency of CUL5 was detected by immunoblotting with an anti-CUL5 antibody. The shRNA sequences are blown.

shCul5 no.1: 5’-ACACAAGCACCCTCGTATTTA-3’;

no. 2: 5’-ATTCATGAAGAGCCCATTATC-3’;

no. 3: 5’-TGCAAGAGATAAGGCGTATAA-3’;

no. 4: 5’-AGCAGTTACTTACACTATTTA-3’.

### RNA isolation and quantitative real-time PCR

Total cell RNA was extracted using TRIzol reagent (Invitrogen) following the manufacturer’s instructions. One microgram of total RNA was subjected to reverse transcription to synthesize cDNA using the RevertAid First Strand cDNA Synthesis Kit (Thermo). qRT-PCR was performed using SYBR Green Master Mix (Monad), and a 10 μl volume reaction consisted of 1 μl reverse transcription product and 300 nM of each primer. Relative gene mRNA levels for each target gene were calculated by the 2^−△△Ct^ method using β-actin as an internal control. The primers used for indicated gene products were described in Supplementary Table 3.

### Protein 3D structure prediction

The 3D structures of SARS-CoV N protein (1ssk) and SARS-CoV-2 N protein (7cdz) were downloaded from PDB. The protein structures of both ORF9b were predicated by the iterative threading assembly refinement (I-TASSER) server, which generated three-dimensional (3D) atomic models from multiple threading alignments and iterative structure assembly simulations. The ubiquitination sites were labeled with PyMOL.

### Dual-luciferase reporter gene assay

The IFNβ and NF-κB promoter-driven firefly luciferase reporter plasmid was transfected into HEK293T cells seeded in 12-well plates with Renilla luciferase expression vectors at a ratio of 10:1 (Firefly: Renilla). Twenty-four hours after transfection, the innate immune pathway was activated by Sendai virus (100 HAU/ml) infection for 12 h, poly (I:C) transfection for 24 h, or RIG-I-N transfection for 24 h. After washing once with PBS, the cells were lysed to measure luciferase activity (Dual-Luciferase Reporter Gene Assay Kit, 11402ES60, Yeasen). The relative Firefly luciferase activity levels were normalized to the luciferase activity of the Renilla control plasmid. Data represent the mean ± SD of three independent experiments.

### Cell viability assays

Cell viability was assayed by the CCK-8 reagent (HY-K0301, MedChemExpress) following with the manufacturer’s protocol. In brief, Calu3 cells (3 × 10^3^ cells/well) were transferred into a 96-well plate and treated with GA or 17-AAG after incubation of 24 h. The CCK-8 reagent was added (10 μl per well) after the indicated incubation times of drugs, and 450 nm OD values were determined with a microplate reader after 1 h of incubation.

### Immunohistochemistry analysis

A total of 10 paired tumor samples and matched adjacent tissues were collected from patients with colorectal cancer (5 for colon adenocarcinoma and 5 for rectum adenocarcinoma) at Xiangya Hospital of Central South University. All patients were previously untreated. Colorectal cancer patient tissues were fixed in 10% neutral buffered formalin (Sigma) overnight at room temperature. Tissues were then dehydrated, embedded in paraffin, and cut into 3 μm slices. After antigen retrieval, tissue slices were subject to immunohistochemical staining with anti-HSP90α primary antibody.

### Single-cell sequencing analysis

The single-cell dataset was obtained from the publicly available data: GSE171524, GSE132771, GSE150728, GSE164985 and GSE161277. The corresponding dataset was analyzed using the Seurat, SingleR package in R language. The data were normalized using the NormalizeData function in the Seurat package and then down-dimensioned using the nonlinear dimensionality reduction method UMAP. Based on the PCA dimensionality reduction results, 15 major components were selected for subsequent cell clustering, and the expression of HSP90α in different cell types was visualized and analyzed using FeaturePlot and VlnPlot methods.

### Dimerization assay

The HEK293T cells transfected with plasmids containing ORF9b were lysed in lysing buffer (50 mM Tris[pH7.4], 150 mM NaCl, 1% NP-40, 0.25% sodium deoxycholate) 48 h after transfection. The lysates were centrifugated, and the supernatants were mixed with a non-reducing loading buffer without SDS or DTT. Subsequently, the samples were separated with 15% SDS–PAGE. The monomer and dimer of ORF9b were detected with an anti-ORF9b antibody.

### Virus infection

For the infection of HeLa-hACE2 cells (MOI = 0.1), Huh7 cells (MOI = 1) and Calu3 cells (MOI = 1), the virus was added directly to the cells and incubated for one hour at 37 °C with 5% CO_2_. Following this, the infected media was removed, and the cells were incubated in 2% FBS media under 37 °C with 5% CO_2_ conditions before being harvested for analysis at the indicated time points. Anti-SARS-CoV-2 Nucleocapsid antibody was used as the primary antibody. All experiments involving the infection of SARS-CoV-2 were conducted within the biosafety level-3 (BSL-3) laboratory located at Shenzhen Third People’s Hospital, following standard operating procedures.

### SARS-CoV-2 K67R-VLP or WT-VLP construction and infection

Vero-ORF3-E cells were initially seeded in a T175 flask and cultured in a DMEM medium with 100 ng/mL of doxycycline. The following day, 40 μg of WT-ΔORF3-E mNG and K67R-ΔORF3-E mNG RNA were electroporated into 8 × 10^6^ Vero-ORF3-E cells using the Gene Pulser XCell electroporation system (Bio-Rad, Hercules, CA) at a setting of 270 V and 950 μF with a single pulse. The electroporated cells were then transferred to a T75 flask and maintained in doxycycline (Sigma-Aldrich)-supplemented medium at 37 °C for 3–4 days. Upon completion of the incubation period, the cell culture was collected and centrifuged to remove any precipitate, yielding the VLPs containing the WT and K67R viral genomes, respectively. The infection with SARS-CoV-2 K67R-VLP or WT-VLP was conducted in the same manner as described in the “Virus infection” section.

### Statistical analysis

Each experiment was repeated three times or more. All results were shown as the mean ± s.d., and Student’s *t*-test (unpaired, two-tailed) was used to compare two independent groups, while two-way ANOVA analysis was performed for comparisons of multiple groups. All statistical analyses were performed with GraphPad Prism 7. *P* < 0.05 was considered statistically significant.

### Supplementary information

Supplementary_Materials

Uncropped Western Blots

Supplementary Table 1

Supplementary Table 2

## Data Availability

The MS spectrometry proteomics are available from the ProteomeXchange Consortium (http://proteomecentral.proteomexchange.org) with the dataset identifier PXD050019, and the original data of the Single-cell sequencing are stored in the GEO public database (https://www.ncbi.nlm.nih.gov/geo/) under the accession numbers GSE171524, GSE132771, GSE150728, GSE164985 and GSE161277. The corresponding authors made the data utilized in the present investigation accessible to interested individuals upon a reasonable request.
