# Supplementary material for: A Cullin 5-based complex serves as an essential modulator of ORF9b stability in SARS-CoV-2 replication
Source: Signal Transduct Target Ther. 2024 Jun 28;9:159. doi: 10.1038/s41392-024-01874-5 (PMC11211426; doi:10.1038/s41392-024-01874-5)
Supplement: Supplementary file 1 — Supplementary_Materials [file 41392_2024_1874_MOESM1_ESM.docx]

Supplementary Materials for

**A Cullin 5-based complex serves as an essential modulator of ORF9b stability in SARS-CoV-2 replication**

Yuzheng Zhou^1,2#^, Zongpeng Chen^1#^, Sijie Liu^1^, Sixu Liu^1^, Yujie Liao^1^, Ashuai Du^1^, Zijun Dong^3^, Yongxing Zhang^1^, Xuan Chen^1^, Siyi Tao^1^, Xin Wu^4^, Aroona Razzaq^1^, Gang Xu^5^, De-an Tan^6^, Shanni Li^1^, Youwen Deng^4^, Jian Peng^7^, Shuyan Dai^8^, Xu Deng^8^, Xianwen Zhang^9^, Taijiao Jiang^10^, Zheng Zhang^2^, Gong Cheng^9,11^, Jincun Zhao^2,10,12^ and Zanxian Xia^1,13*^

Correspondence to: xiazanxian@sklmg.edu.cn

**This PDF file includes:**

Supplementary Fig. 1 to Fig. 12

Supplementary Table. 3 to Table. 4

Supplementary Fig. 1.

**
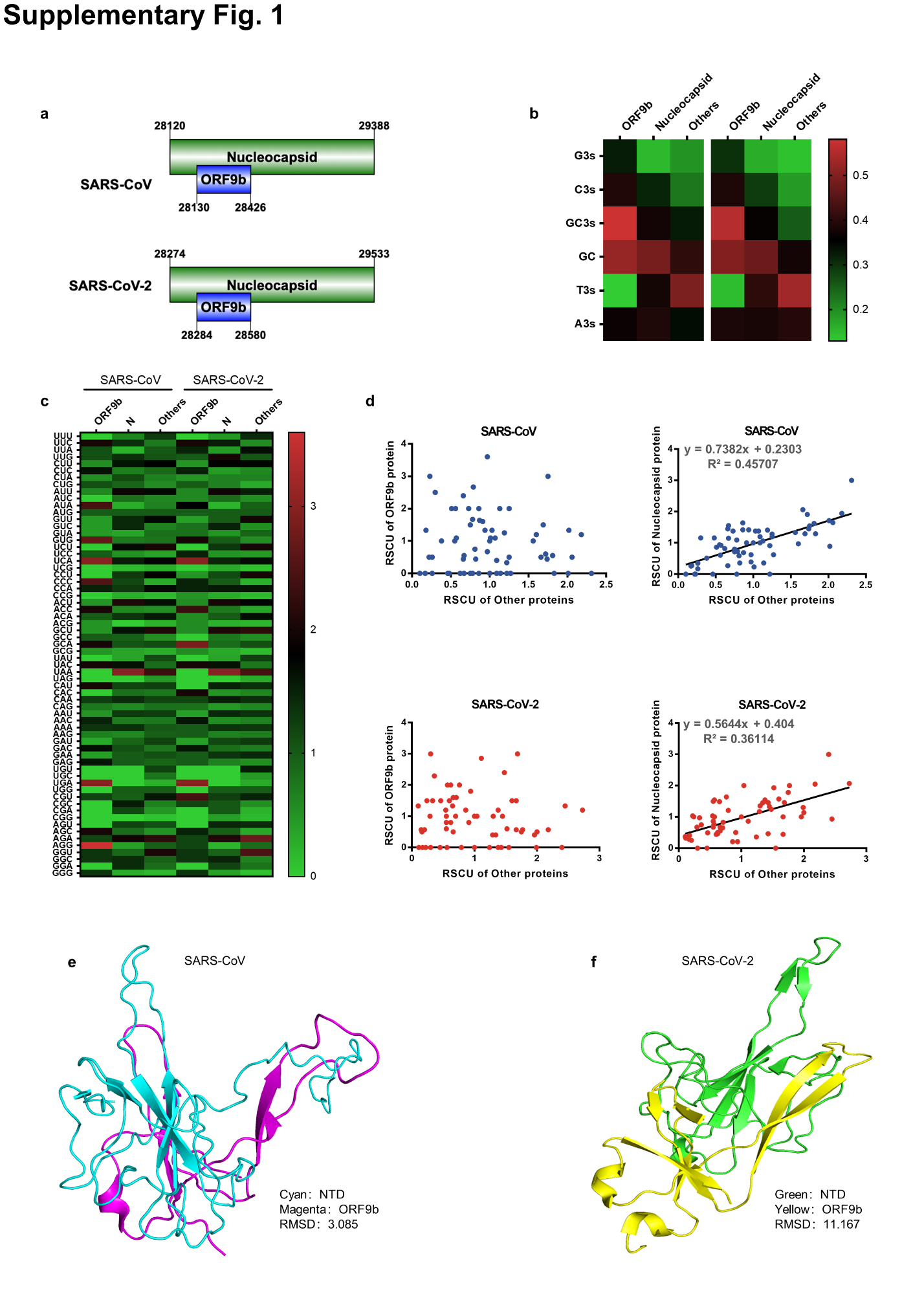
**

**Supplementary Fig. 1 Evolutionary analysis of *N* and *ORF9b* genes of SARS-CoV and SARS-CoV-2 (related to Fig. 1). a** Schematic diagrams of *N* and *ORF9b* genes of both SARS-CoV and SARS-CoV-2. **b** Content of different bases at the third position of the codon and total GC in *ORF9b*，*N*, and other genes, of both SARS-CoV and SARS-CoV-2. **c** Relative synonymous codon usage of *ORF9b*, *N*, and other genes, of both SARS-CoV and SARS-CoV-2. **d** Dot plot and Pearson’s correlation showing the relative synonymous codon usage of *ORF9b* and other genes, or *N*, and other genes of SARS-CoV and SARS-CoV-2. **e-f** The 3D structure alignment between the SARS-CoV ORF9b protein (Magenta) and N-terminal of N protein (NTD, cyan), RMSD=3.085 (**e**). The 3D structure alignment between the SARS-CoV-2 ORF9b protein (yellow) and N-terminal of N protein (NTD, green), RMSD=11.167 (**f**).

Supplementary Fig. 2.


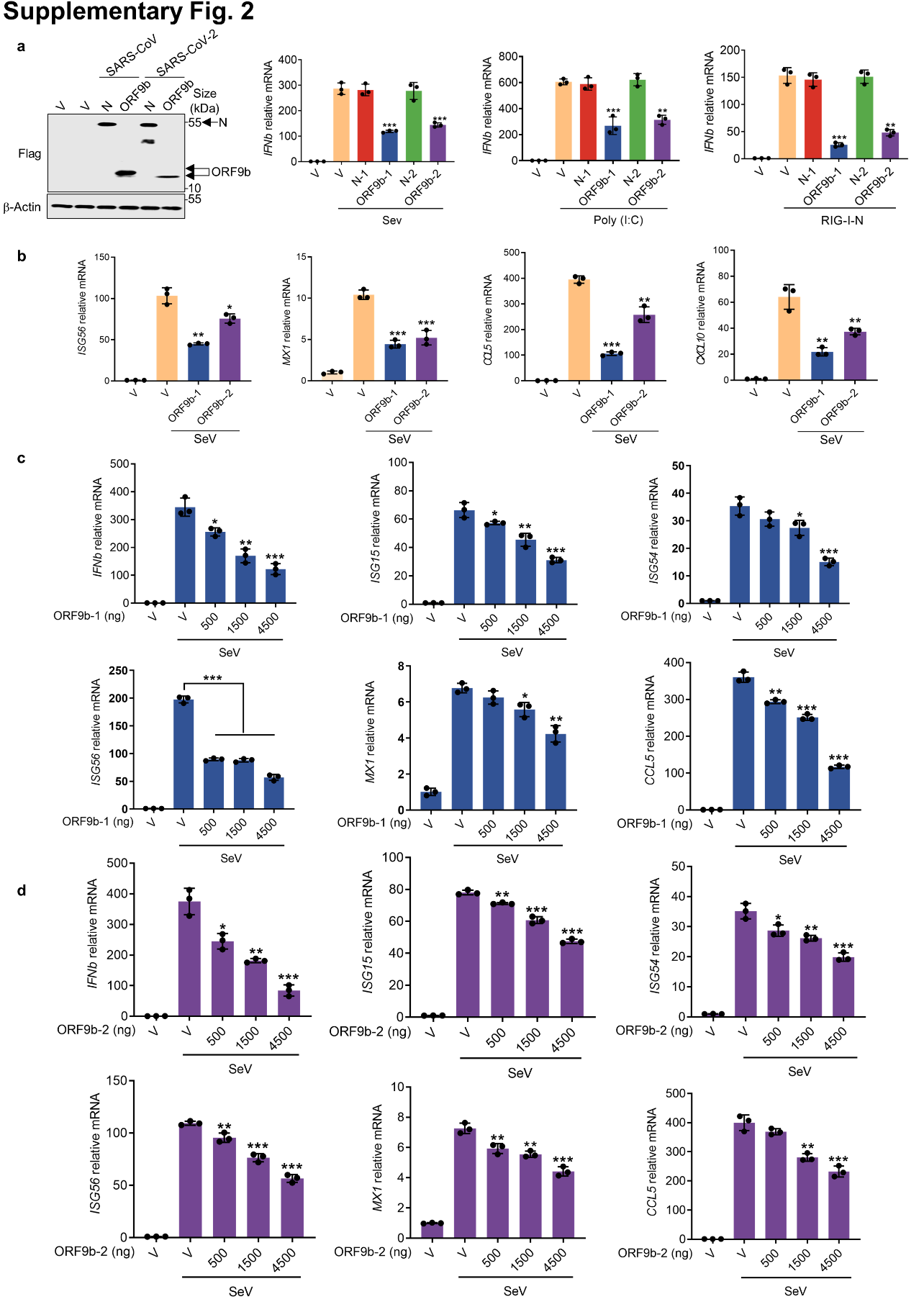


**Supplementary Fig. 2 SARS-CoV-2 ORF9b and SARS-CoV ORF9b can antagonizes host innate immunity (related to Fig. 1). a** HEK293T cells were transfected with either an empty vector or plasmids expressing viral genes (SARS-CoV *N* (N-1); SARS-CoV *ORF9b* (ORF9b-1); SARS-CoV-2 *N* (N-2); SARS-CoV-2 *ORF9b* (ORF9b-2)) . Subsequently, they were infected with SeV (100 HAU/ml) for 12 h, transfected with Poly(I:C) for 12 h, or co-transfected with the RIG-I-N plasmid for 24 h. Total RNA was extracted, reverse transcribed and analyzed by qRT-PCR with primers specific for *IFNb*. **b** HEK293T cells transfected with an empty vector or ORF9b from both SARS-CoV and SARS-CoV-2 were infected with SeV for 12 h. Cells were collected for qRT-PCR with primers specific for *ISG56*, *MX1*, *CCL5*, and *CXCL10*. **c-d** SARS-CoV ORF9b (**c**) or SARS-CoV-2 ORF9b (**d**) were expressed in gradients in HEK293T cells. Then the cells were infected with SeV for 12 h and collected for qRT-PCR to test the indicated genes.

Quantification was shown as mean±s.d. *n*=3 independent experiments. Student’s *t* test (unpaired, two-tailed) was used to compare two independent groups, and two-way ANOVA test was performed for comparisons of multiple groups. **P*<0.05; ***P*<0.01; ****P*<0.001.

Supplementary Fig. 3.

**
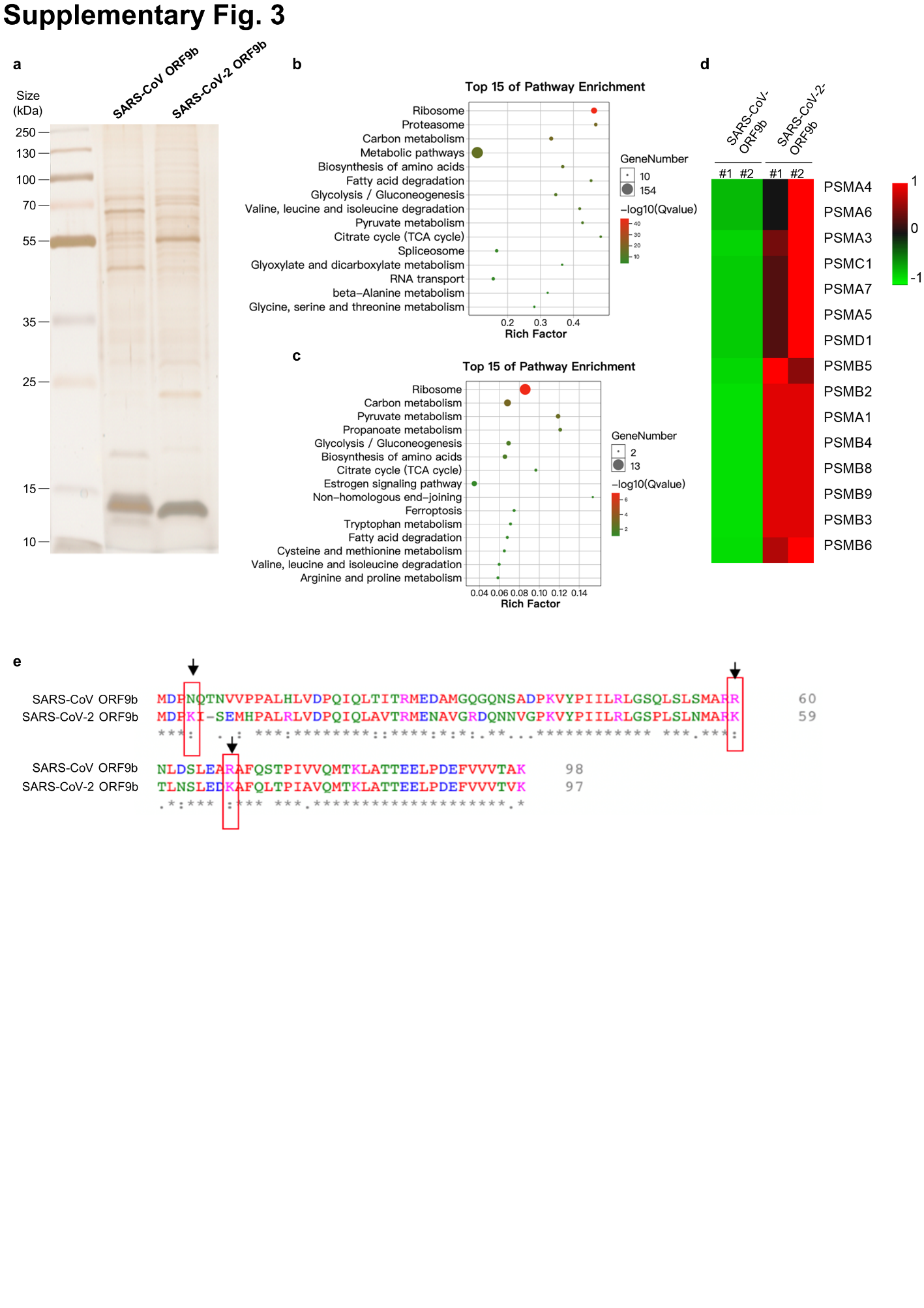
**

**Supplementary Fig. 3 SARS-CoV-2 ORF9b was correlated with the ubiquitin-proteasome pathway (related to Fig. 1). a** The ORF9b protein of both SARS-CoV and SARS-CoV-2 and the interacting proteins were purified from HEK293T cells, and detected by silver staining. **b** The KEGG enrichment analysis of SARS-CoV-2 ORF9b interacting proteins. **c** The KEGG enrichment analysis of SARS-CoV ORF9b interacting proteins. **d** Heatmap shows the differences between the ORF9b interacting proteins enrichment in proteasome pathway of two coronaviruses. **e** The amino acid sequences of SARS-CoV and SARS-CoV-2 ORF9b proteins were aligned by ClustalW. The lysines unique to SARS-CoV-2 were shown.

Supplementary Fig. 4.

**
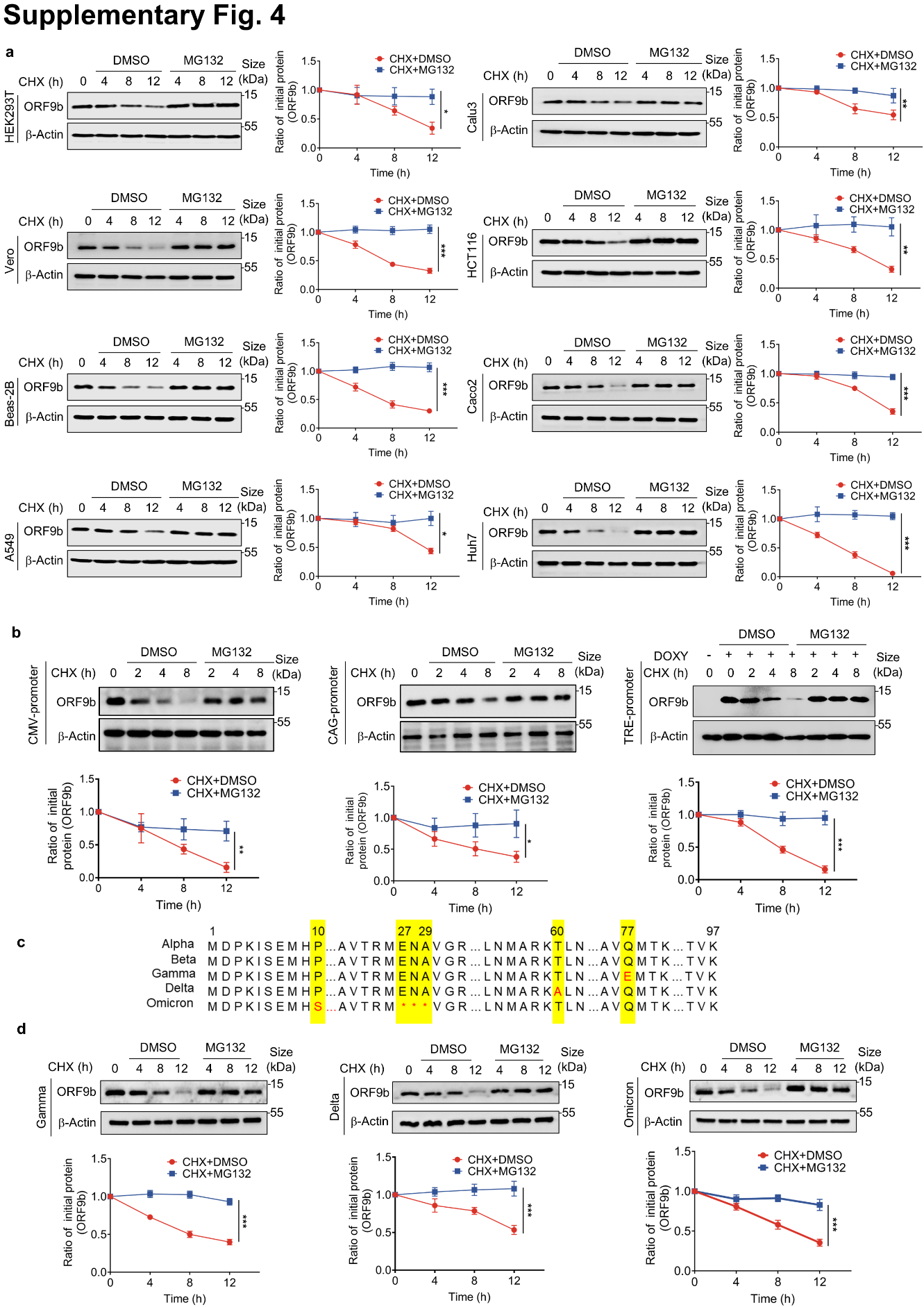
**

**Supplementary Fig. 4 Degradation of SARS-CoV-2 ORF9b via the ubiquitin-proteasome pathway (related to Fig. 1). a** HEK293T, Vero, Beas-2B, A549, Calu3, HCT116, Caco2 and Huh7 cells transfected with Flag-tagged ORF9b plasmid were co-treated with CHX and MG132 to analyze the half-life of ORF9b proteins. Cells were collected at the indicated time for Western blot. **b** Coding sequence of SARS-CoV-2 ORF9b was inserted into plasmids with CMV, CAG, and TRE promoter, respectively. 24 h after transfection, the cells were treated with CHX (50 μg/ml) for specified durations before collection. The expression of pTRE-ORF9b was induced by Doxycline. **c** Amino acid alignment of SARS-CoV-2 variants. The mutations were marked in red. **d** Half-life analyses of ORF9b from different variants in HEK293T cells when co-treating with CHX and MG132.

Quantification was shown as mean±s.d. *n*=3 independent experiments. *, *P*<0.05, **, *P*<0.01, ***, *P*<0.001. Student’s *t* test (unpaired, two-tailed) was used to compare two independent groups, and two-way ANOVA test was performed for comparisons of multiple groups. **P*<0.05; ***P*<0.01; ****P*<0.001.

Supplementary Fig. 5.

**
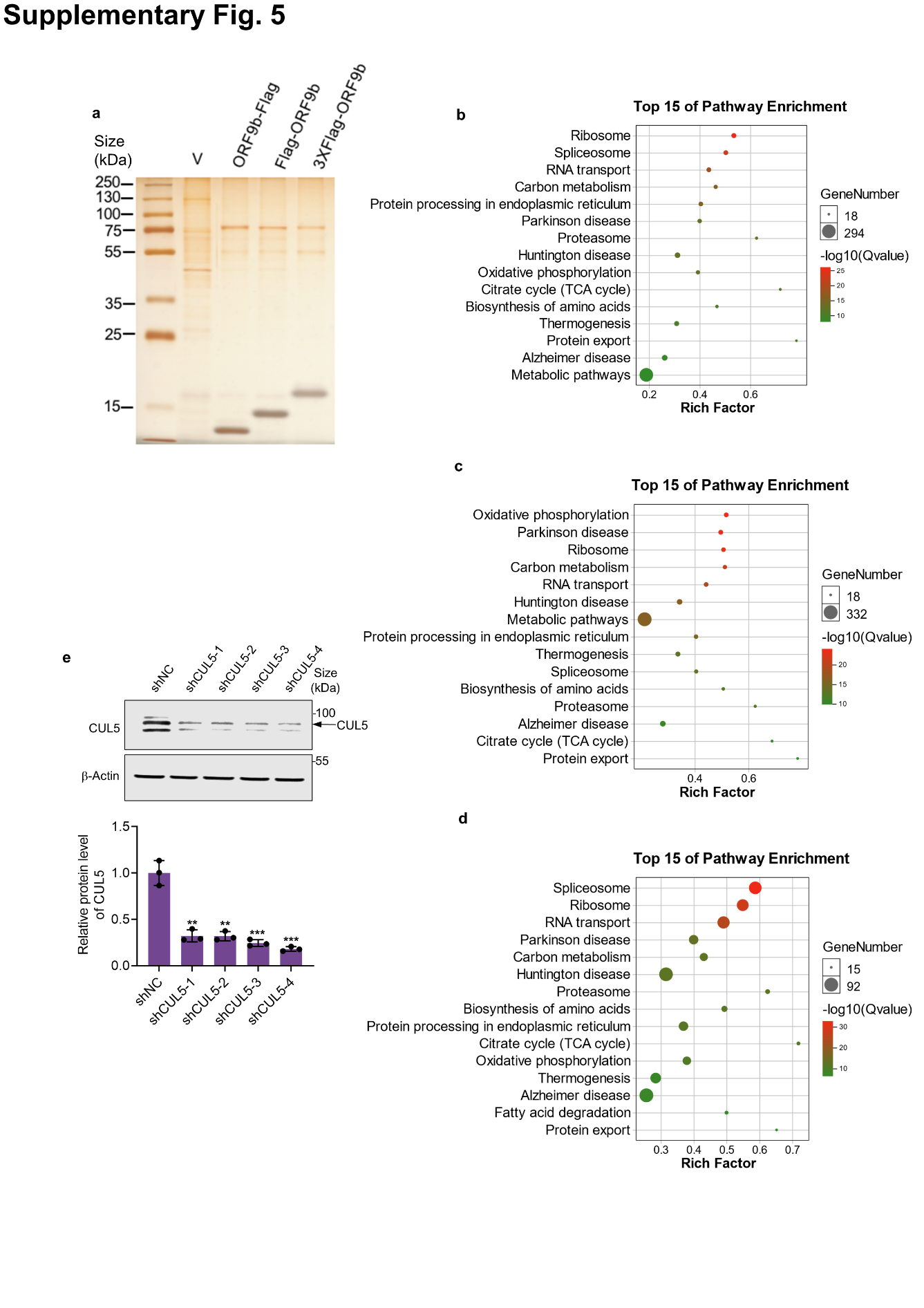
**

**Supplementary Fig. 5 CUL5 induces the degradation of ORF9b (related to Fig. 2). a** HEK293T cells were transfected with vector, Flag-ORF9b, ORF9b-Flag and 3xFlag-ORF9b. 24 h post transfection, the cells were treated with MG132 (10 μM) for 8 h. ORF9b and its interacting proteins were co-precipitated by anti-Flag agarose beads and analyzed by silver staining. **b-d** The KEGG enrichment analysis for the Flag-ORF9b (**b**), ORF9b-Flag (**c**) and 3×Flag-ORF9b (**d**) interacting proteins. **e** Four shRNAs targeting *CUL5* were packaged into the lentivirus and infected with HEK293T cells, respectively. CUL5 protein levels was detected by Western blot.

Quantification was shown as mean±s.d. *n*=3 independent experiments. Student’s *t* test (unpaired, two-tailed) was used to compare two independent groups. ***P*<0.01; ****P*<0.001.

Supplementary Fig. 6.

**
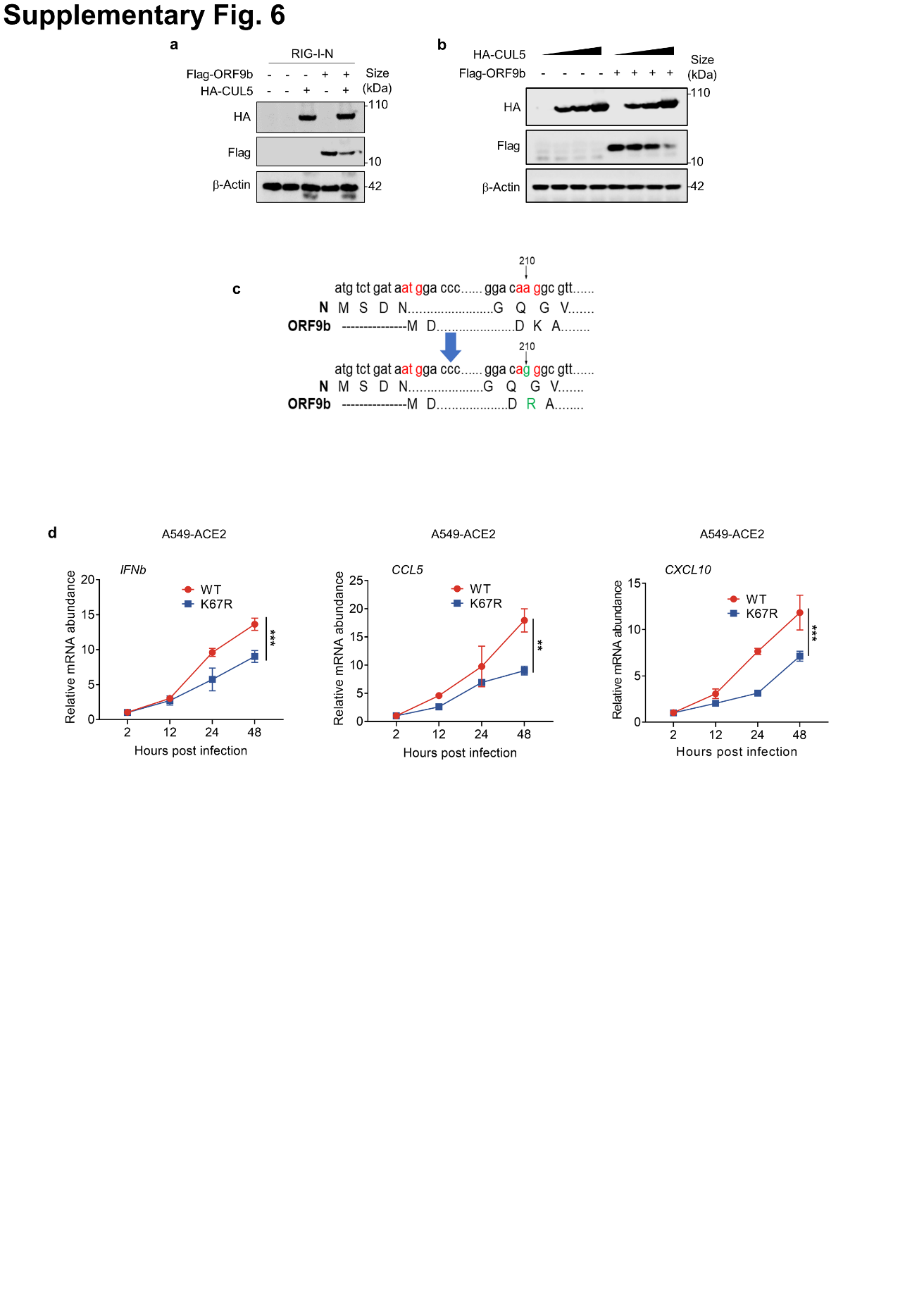
**

**Supplementary Fig. 6 CUL5 modulates the immunosuppressive effects of ORF9b and functions as a host antiviral factor (related to Fig. 3). a** The sample from Fig.3a was also collected for Western blot to test expression of SARS-CoV-2 ORF9b and CUL5 (related to Fig.3a). **b** Parts of the sample from Fig.3c were detected by Western blot for expression of CUL5 and ORF9b (related to Fig.3c). **c** A strategy of “K to R” mutation at the K67 site of ORF9b protein in the VLP system. The base at 210 on the *N* gene was mutated from “a to g” and labeled green. **d** The A549-ACE2 cells were infected with SARS-CoV-2 K67R-VLP or WT-VLP. Cells were collected as indicated time for qRT-PCR to detect *IFNb*, *CCL5*, and *CXCL10*.

Quantification was shown as mean±s.d. *n*=3 independent experiments. Student’s *t* test (unpaired, two-tailed) was used to compare two independent groups, and two-way ANOVA test was performed for comparisons of multiple groups. ***P*<0.01; ****P*<0.001.

Supplementary Fig. 7.

**
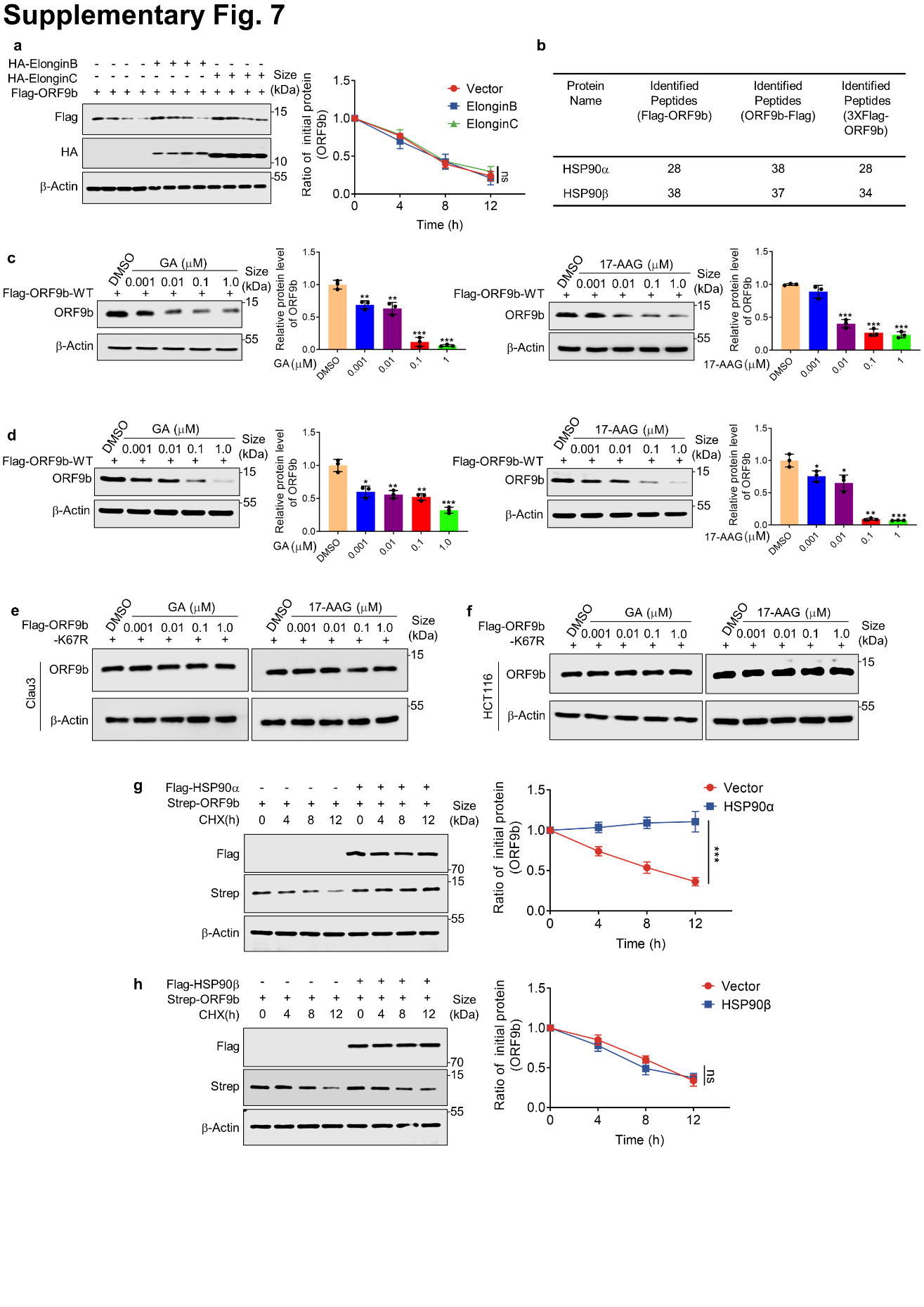
**

**Supplementary Fig. 7 HSP90α maintains the stability of ORF9b (related to Fig. 4). a** Half-life analyses of ORF9b proteins when overexpressing Elongin B, Elongin C or not in HEK293T cells. **b** The identified peptides of HSP90α and HSP90β co-precipitated with Flag-ORF9b, ORF9b-Flag, or 3xFlag ORF9b. **c-d** Calu3 (**c**) and HCT116 (**d**) cells overexpressing Flag-ORF9b were treated with indicated concentration of GA or 17-AAG for 24 h. The cells were collected and lysed to detect the protein level of ORF9b. **e-f** Calu3 (**e**) and HCT116 (**f**) cells overexpressing Flag-ORF9b-K67R were treated with indicated concentration of GA or 17-AAG for 24 h. The cells were collected and lysed to detect the protein level of ORF9b. **g-h** Half-life analyses of ORF9b when overexpressing HSP90α (**g**) or HSP90β (**h**) in HEK293T cells.

Quantification was shown as mean±s.d. *n*=3 independent experiments. Student’s *t* test (unpaired, two-tailed) was used to compare two independent groups, and two-way ANOVA test was performed for comparisons of multiple groups. ***P*<0.01; ****P*<0.001; n.s, not significant.

Supplementary Fig. 8.

**
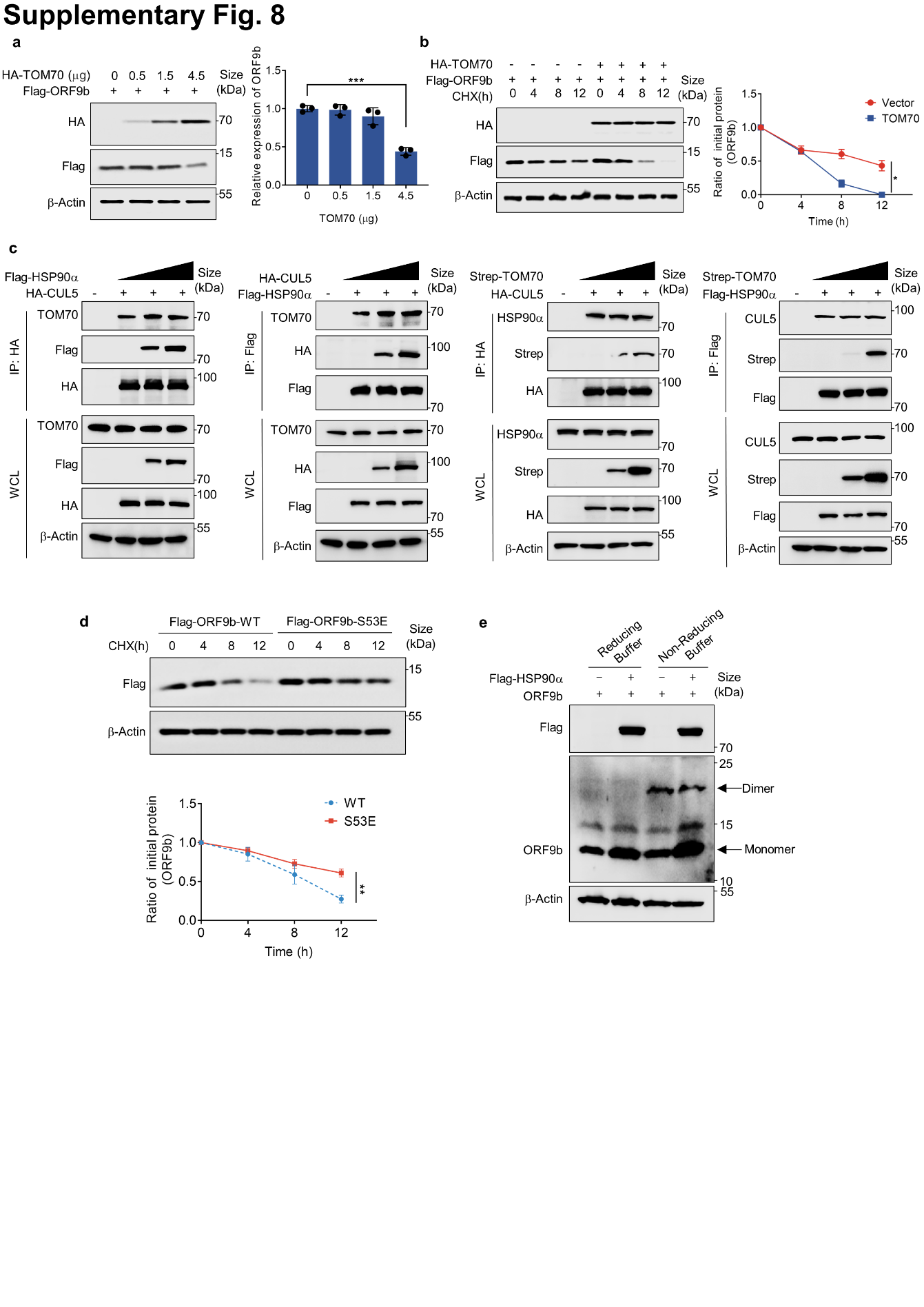
**

**Supplementary Fig. 8 TOM70 serves as a substrate receptor of CUL5-based E3 ligase for ORF9b (related to Fig. 5). a** The ORF9b protein level was detected in HEK293T cells transfected with a gradually increasing amount of plasmids containing HA-TOM70 (left). The quantification of ORF9b protein level was normalized to β-actin (right). **b** Half-life analyses of SARS-CoV-2 ORF9b protein when overexpressing HA-TOM70 or not in HEK293T cells (left). The quantification of ORF9b protein level was normalized to β-actin (right). **c** HEK293T cells were transfected with indicated plasmids. The cells were then lysed and immunoprecipitated with anti-Flag beads or anti-HA beads 48 h after transfection. The whole cell lysates and precipitated proteins were analyzed by immunoblotting with indicated antibodies. **d** Half-life analyses of SARS-CoV-2 ORF9b-WT and SARS-CoV-2 ORF9b-S53E (up). The quantification of ORF9b protein level was normalized to β-actin (down). **e** HEK293T cells transfected with plasmids containing ORF9b were lysed in reducing buffer (with SDS and DTT) or non-reducing buffer (without SDS and DTT). The samples were separated with 15% SDS-PAGE. The monomer and dimer of ORF9b were detected with an anti-ORF9b antibody.

Quantification was shown as mean±s.d. *n*=3 independent experiments. Student’s *t* test (unpaired, two-tailed) was used to compare two independent groups, and two-way ANOVA test was performed for comparisons of multiple groups. **P*<0.05; ***P*<0.01.

Supplementary Fig. 9.

**
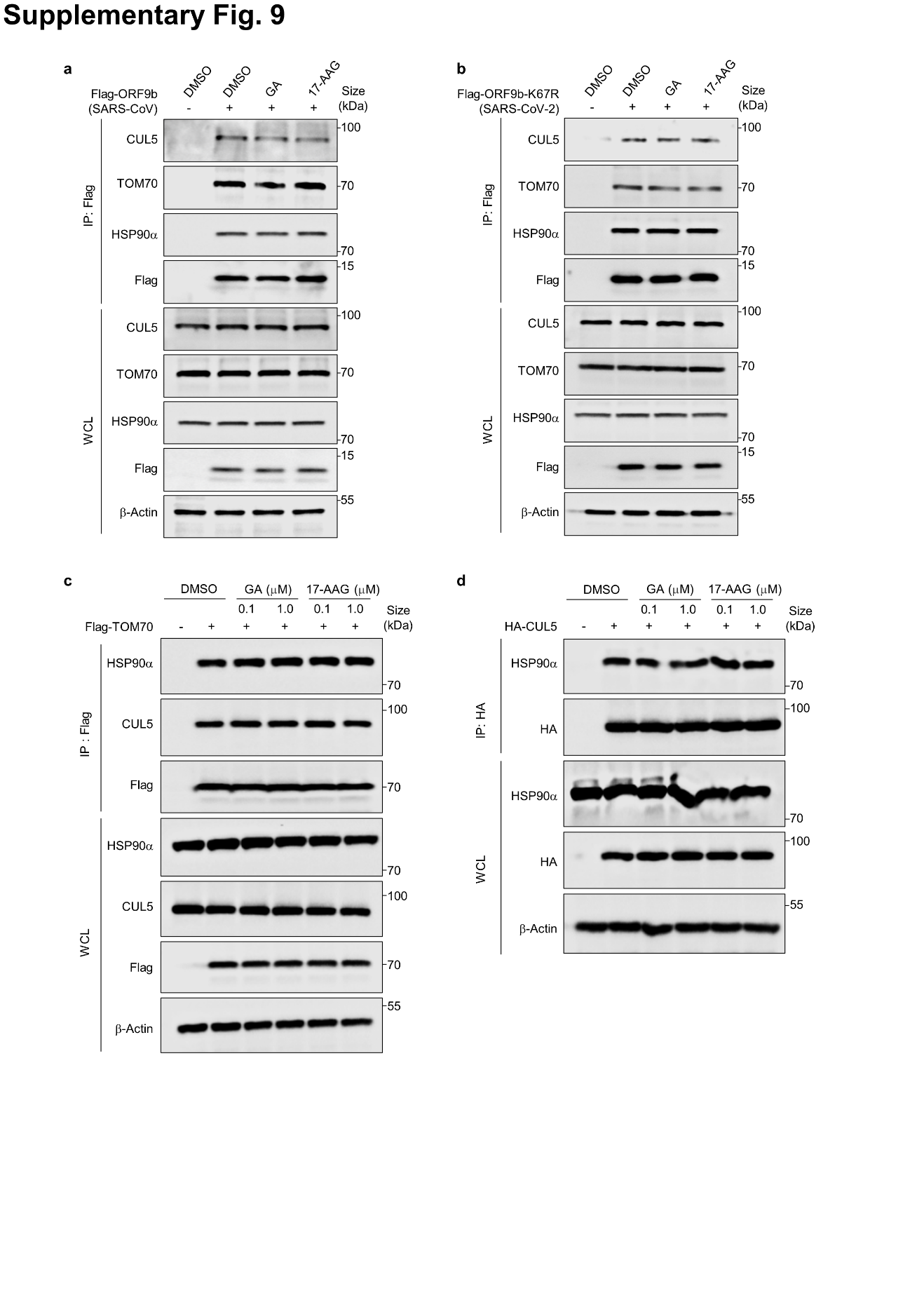
**

**Supplementary Fig. 9 HSP90 inhibitors and K67R mutation of ORF9b cannot influence the interaction of ORF9b and CUL5-TOM70-HSP90α complex (related to Fig. 5). a-b** HEK293T cells were transfected with plasmids containing SARS-CoV Flag-ORF9b (**a**) or SARS-CoV-2 Flag-ORF9b-K67R (**b**) for 12 h and then treated with DMSO, GA or 17-AAG for 24 h as indicated. The cells were lysed and immunoprecipitated with anti-Flag beads. The whole cell lysates and precipitated proteins were analyzed by immunoblotting with indicated antibodies. **c-d** HEK293T cells were first transfected with plasmids containing Flag-TOM70 (**c**) or HA-CUL5 (**d**) for 12 h and then treated with DMSO, GA or 17-AAG for 24 h as indicated. The cells were lysed and immunoprecipitated with anti-Flag or anti-HA beads. The whole cell lysates and precipitated proteins were analyzed by immunoblotting with indicated antibodies.

Supplementary Fig. 10.

**
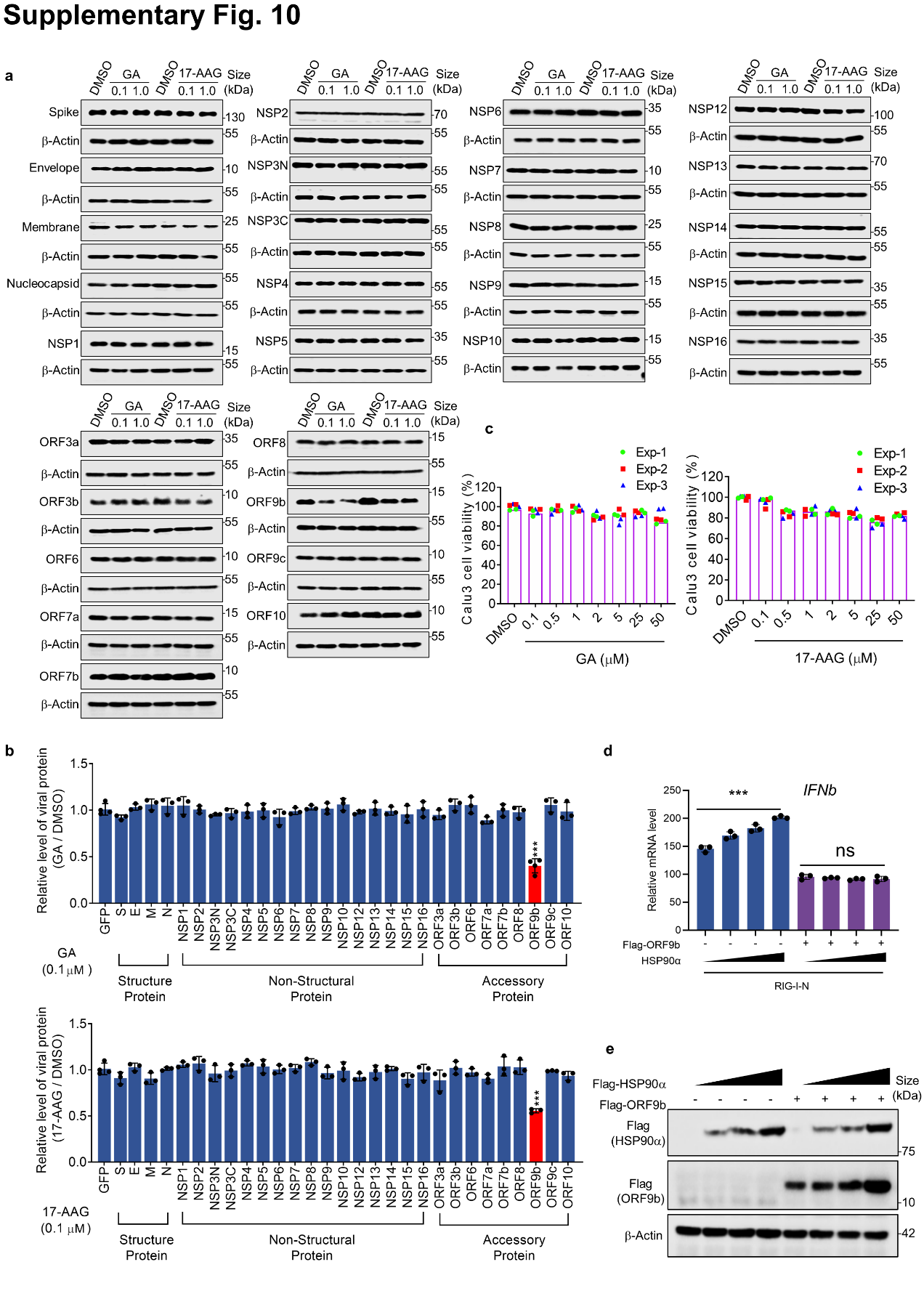
**

**Supplementary Fig. 10 GA and 17-AAG can downregulate the level of ORF9b but not the other proteins of SARS-CoV-2 (related to Fig. 6). a-b** The plasmids containing Strep-tagged SARS-CoV-2 viral genes were transfected into HEK293T cells and treated with indicated concentrations of GA or 17-AAG for 24 h. The cells were lysed to detect the viral protein levels (**a**) and quantification of different viral proteins normalized to β-actin was shown as mean±s.d. n=3 independent experiments (**b**). **c** Calu3 cells were treated with either 0.1% DMSO (drug-vehicle) or 0.1-50 μM GA，or 0.1-50 μM 17-AAG. After 24 h, the proportion of viable cells was evaluated by adding CCK-8 reagent. Quantification was shown as mean±s.d. n=6 independent experiments. **d-e** HEK293T cells overexpressing the ORF9b protein or not were transfected with a gradually increasing amount of *HSP90α* plasmid. Cells were transfected with RIG-I-N plasmid for 12 h. The samples were collected for qRT-PCR (**d**) and Western blot (**e**).

Student’s *t* test (unpaired, two-tailed) was used to compare two independent groups. ****P*<0.001; n.s, not significant.

Supplementary Fig. 11.

**
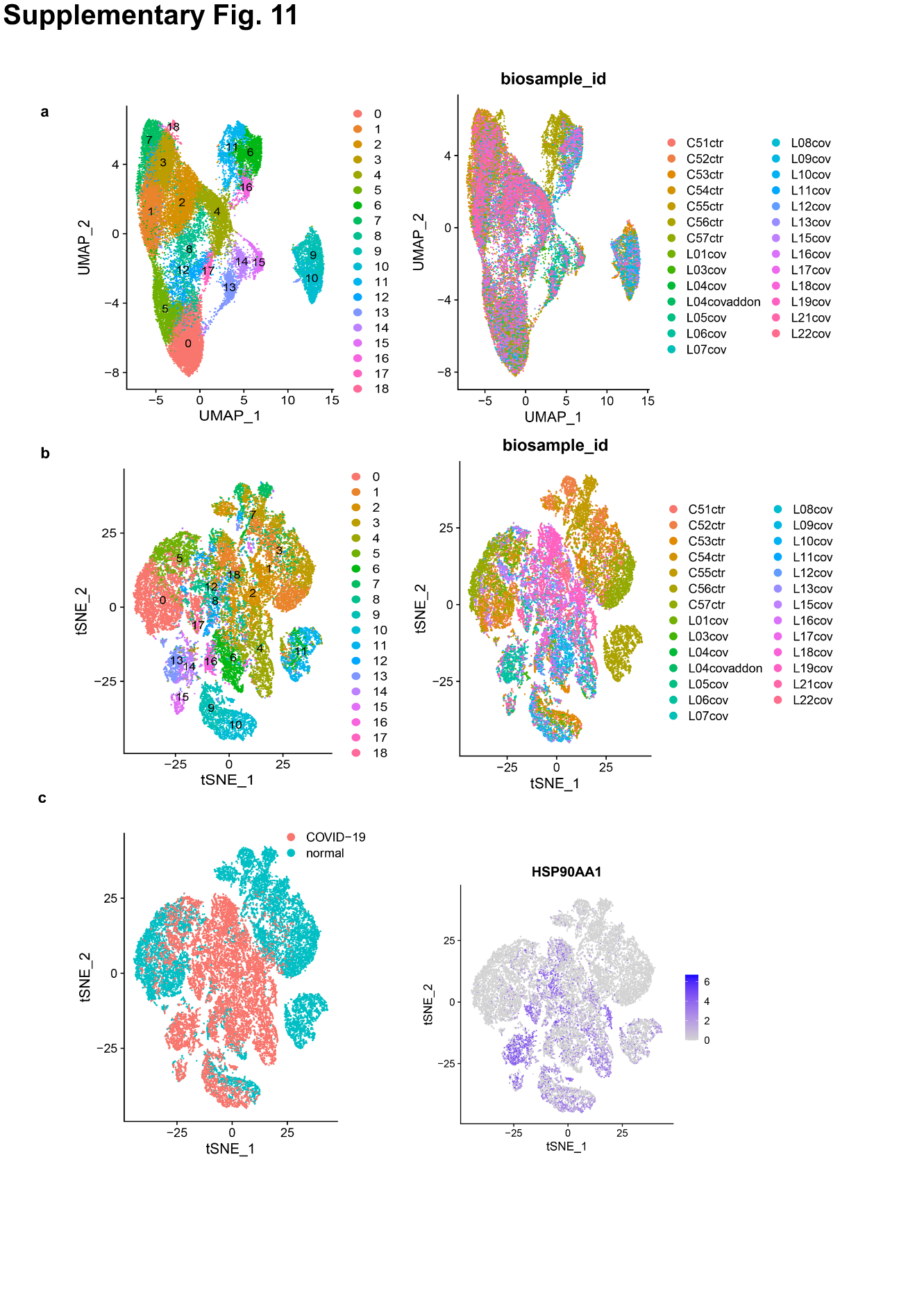
**

**Supplementary Fig. 11 A single-cell analysis of the *HSP90AA1* level in lung epithelial cells of COVID-19 patients (related to Fig. 6).** The single-cell sequencing dataset related to lung epithelial cells of COVID-19 patients with GEO accession number GSE171524 was collected. **a** The UMAP plot showing the clustering and identification of cellular taxa (left) and sample origin (right). **b-c** The tSNE plot showing the clustering and identification of cellular taxa (**b**, left), sample origin (**b**, right), group origins of cells (**c**, left), and RNA levels of *HSP90AA1* in single cells (**c**, right).

Supplementary Fig. 12.

**
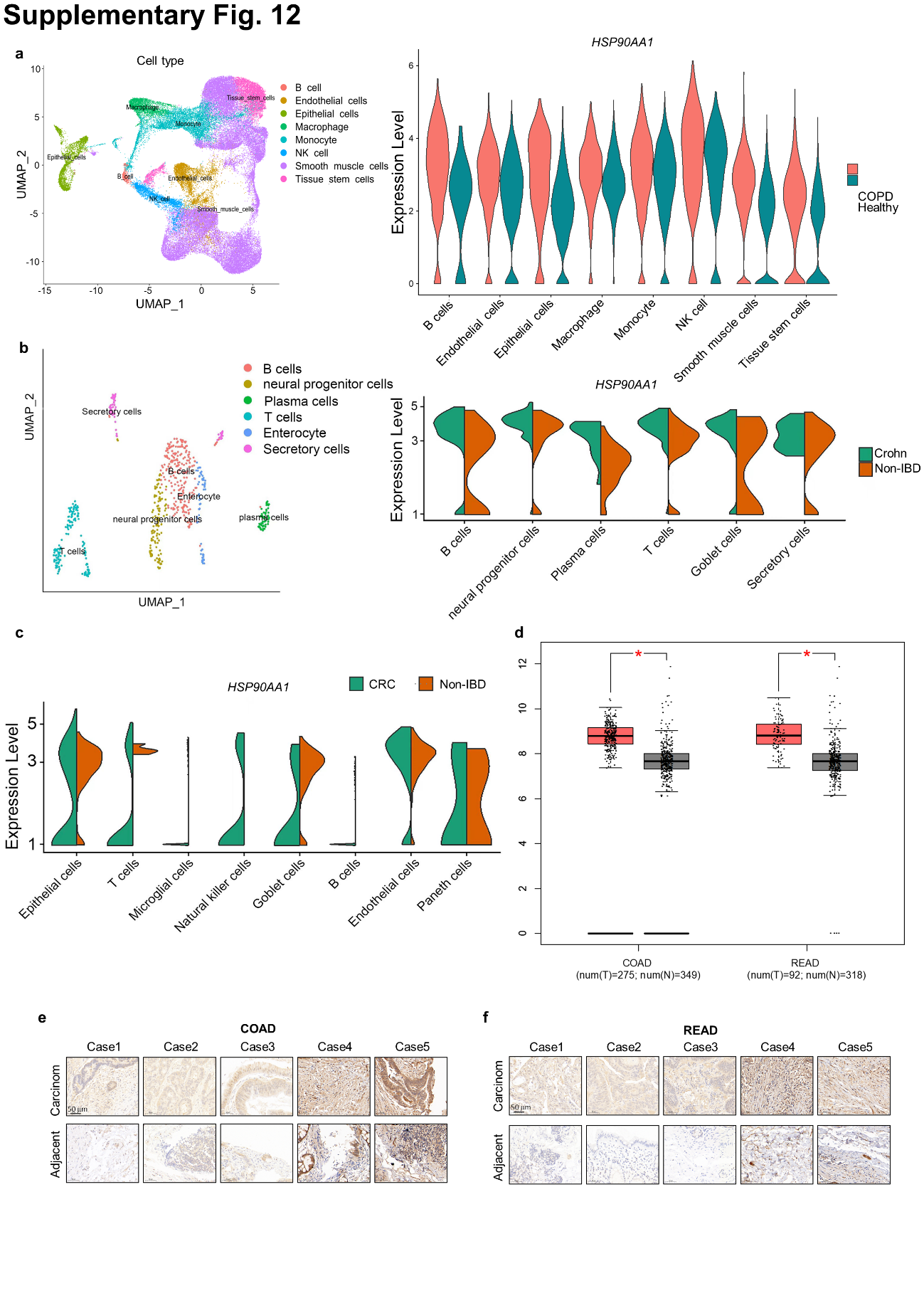
**

**Supplementary Fig. 12 The expression of HSP90α is higher in respiratory and gastrointestinal systemic diseases patients (related to Fig. 6). a** The data from single-cell sequencing databases related to chronic obstructive pulmonary disease (COPD) was collected (GSE132771 and GSE150728). The clustering and identification of cellular taxa was completed firstly (left), and then the expression of *HSP90AA1* in different types of cells was analyzed (right). **b** The data from single-cell sequencing databases related to Crohn’s disease was collected (GSE164985). The clustering and identification of cellular taxa was completed firstly (left), and then the expression of *HSP90AA1* in different types of cells from Crohn’s disease patients and healthy control was compared (right). **c** The expression of *HSP90AA1* in different types of cells from colorectal cancer (CRC) patients was compared to healthy control. The data was collected from the single-cell sequencing dataset with GEO accession number GSE161277. **d** Expression levels of HSP90α proteins were compared between tumor tissue and normal tissue in COAD (Colon adenocarcinoma) and READ (rectum adenocarcinoma) based on the GEPIA data set. **e-f** Immunohistochemistry staining of HSP90α in human COAD and READ tissues, and para-cancerous tissues.

Supplementary Table. 3.

**The primers for detecting mRNA level of genes**

| **Target gene** | **Primer sequence (5’-3’)** |
| --- | --- |
| *Human* *IFNB* | Forward: AGGACAGGATGAACTTTGAC  Reverse: TGATAGACATTAGCCAGGAG |
| *Human* *ISG56* | Forward: TCTCAGAGGAGCCTGGCTAA  Reverse: TGACATCTCAATTGCTCCAG |
| *Human* *ISG54* | Forward: ACGCATTTGAGGTCATCAGGGTG  Reverse:CCAGTCGAGGTTATTTGGATTTGGTT |
| *Human* *ISG15* | Forward: GCGAACTCATCTTTGCCAGTA  Reverse: AGCATCTTCACCGTCAGGTC |
| *Human* *CCL5* | Forward: CCTGCTGCTTTGCCTACATTGC  Reverse: ACACACTTGGCGGTTCTTTCGG |
| *Human* *CXCL10* | Forward: CACCATGAATCAAACTGCGA  Reverse: GCTGATGCAGGTACAGCGT |
| *Human* *MX1* | Forward: GGTGGTGGTCCCCAGTAATG  Reverse: ACCACGTCCACAACCTTGTCT |
| *Human* *ACTB* | Forward: GTTGTCGACGACGAGCG  Reverse: GCACAGAGCCTCGCCTT |
| *SARS-CoV-2 N* | Forward: GGCAGTAACCAGAATGGAGAACG  Reverse: ATGATGCCGTCTTTGTTAGCAC |
| SARS-CoV-2 *S* | Forward: GCTGGTGCTGCAGCTTATTA  Reverse: AGGGTCAAGTGCACAGTCTA |
| *Mouse Ifng* | Forward: TCTTCAGCAACAGCAAGGCGAA  Reverse: TGAGGCTGGATTCCGGCAACA |
| *Mouse Il1b* | Forward: TCGCAGCAGCACATCAACAAGA  Reverse: CCTGGAAGGTCCACGGGAAAGA |
| *Mouse Tnfa* | Forward: TGGAACTGGCAGAAGAGGCACT  Reverse: GTAGACAGAAGAGCGTGGTGGC |
| *Mouse Cxcl9* | Forward: TGGGCATCATCTTCCTGGAGCA  Reverse: TTTGTAGTGGATCGTGCCTCGG |
| *Mouse Il6* | Forward: CACTTCACAAGTCGGAGGC  Reverse: TTTGTATCTCTGGAAGTTTCAG |
| *Mouse Cxcl13* | Forward: TTGTGATCTGGACCAAGATGAA  Reverse: GACTTTTGCTTTGGACATGTCT |
| *Mouse Gapdh* | Forward: AGGTCGGTGTGAACGGATTTG  Reverse: GGGGTCGTTGATGGCAACA |

Supplementary Table. 4.

**Patients’ information in the study.**

| **Patient Number** | **Age** | **Gender** | **Disease** |
| --- | --- | --- | --- |
| 1 | 33 | Female | COAD |
| 2 | 29 | Male | COAD |
| 3 | 33 | Male | COAD |
| 4 | 43 | Female | COAD |
| 5 | 50 | Female | COAD |
| 6 | 30 | Male | READ |
| 7 | 41 | Male | READ |
| 8 | 39 | Female | READ |
| 9 | 51 | Female | READ |
| 10 | 62 | Male | READ |
