# Supplementary material for: A Cullin 5-based complex serves as an essential modulator of ORF9b stability in SARS-CoV-2 replication
Source: Signal Transduct Target Ther. 2024 Jun 28;9:159. doi: 10.1038/s41392-024-01874-5 (PMC11211426; doi:10.1038/s41392-024-01874-5)

Uncropped Western Blots

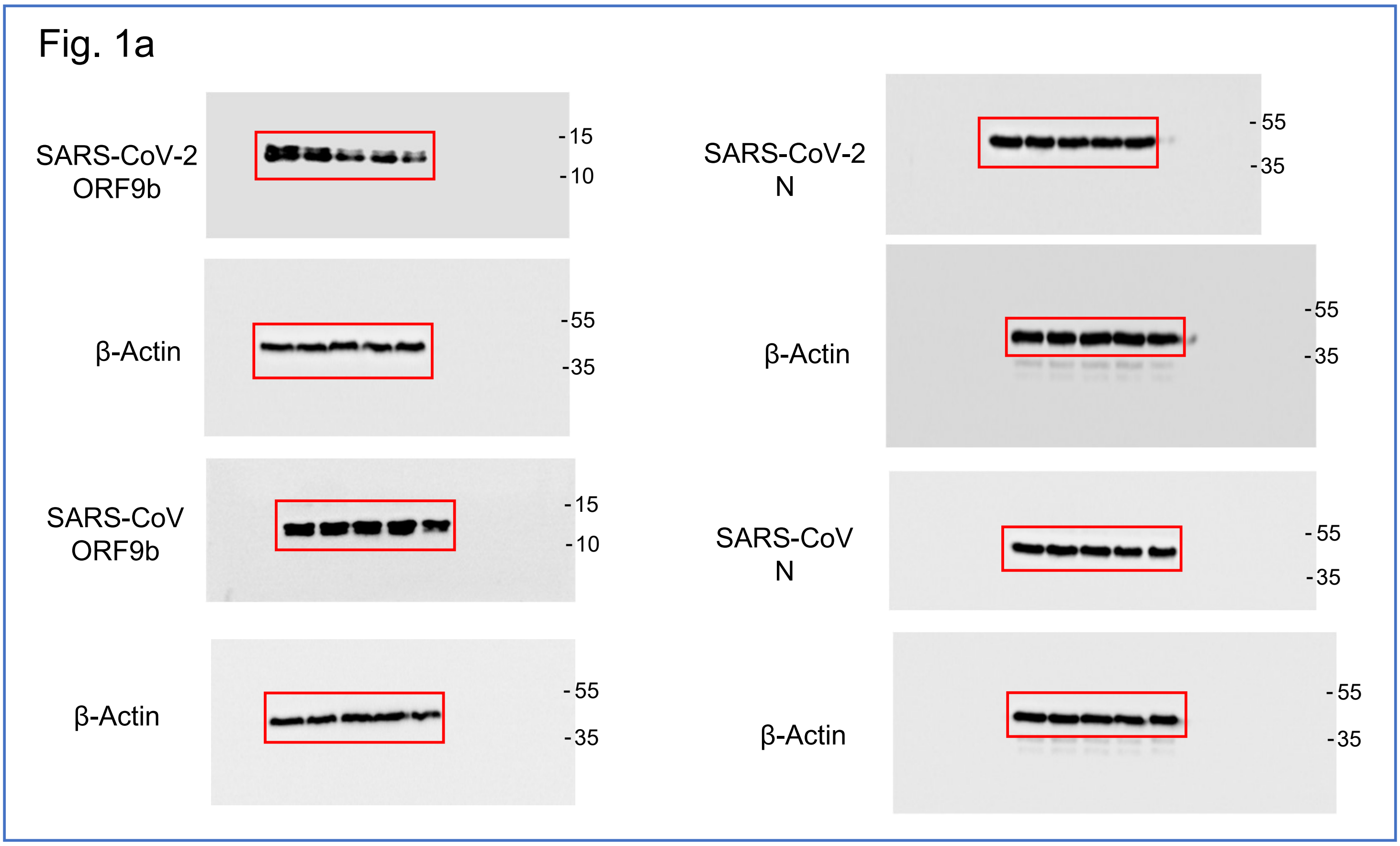
Fig. 1a
SARS-CoV-2 ORF9b
15
10
β-Actin
55
35
SARS-CoV ORF9b
15
10
β-Actin
55
35
SARS-CoV-2 N
55
35
β-Actin
55
35
SARS-CoV N
55
35
β-Actin
55
35

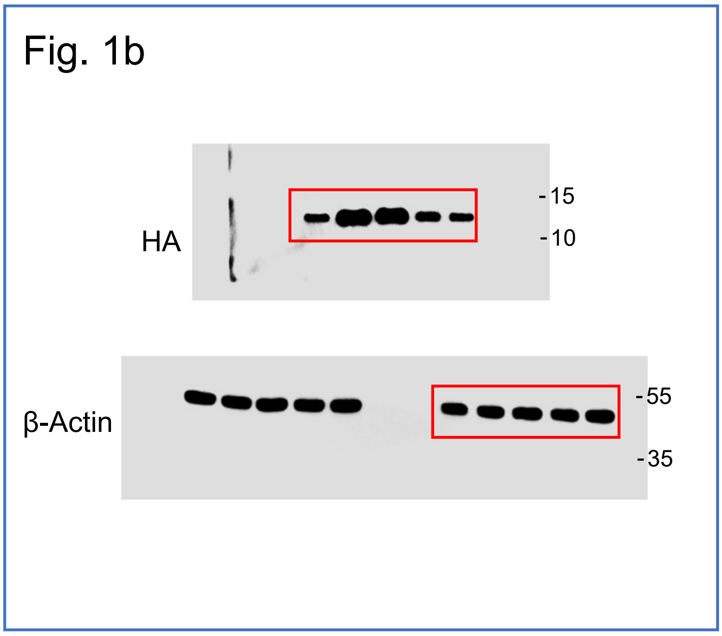
Fig. 1b
HA
15
10
β-Actin
55
35

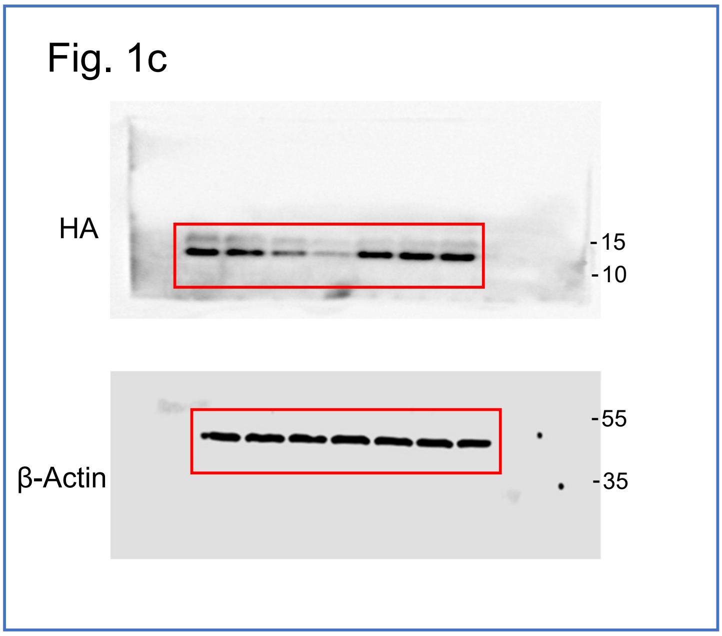
Fig. 1c
HA
15
10
β-Actin
55
35

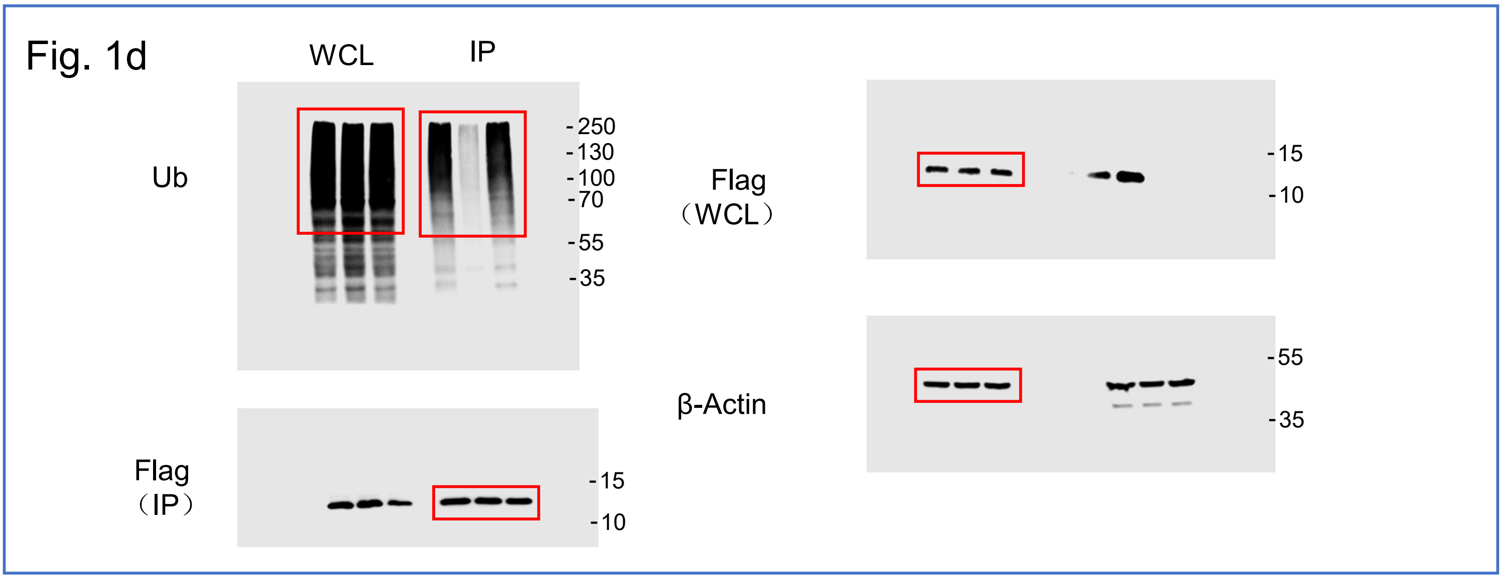
Fig. 1d
WCL
IP
Ub
250
130
100
70
55
35
Flag (IP)
15
10
Flag (WCL)
15
10
β-Actin
55
35

Fig. 1e

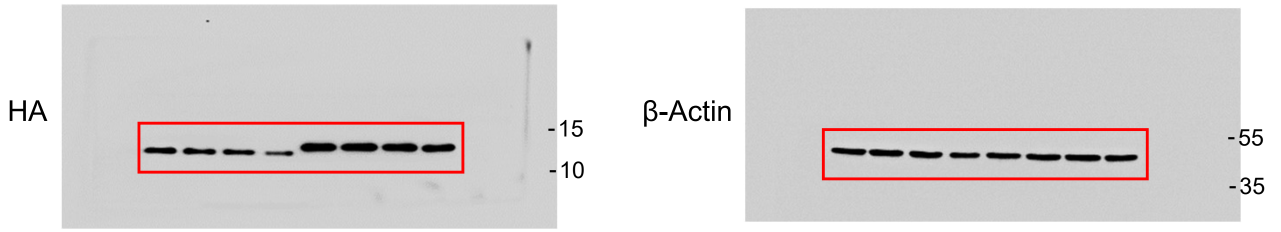

Fig. 1g

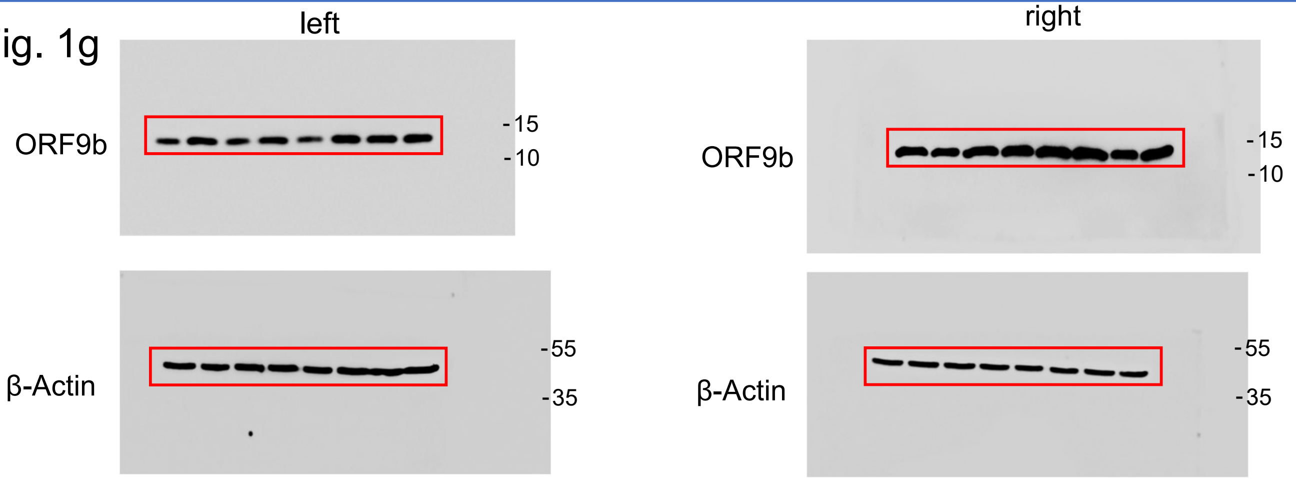

Fig. 1h

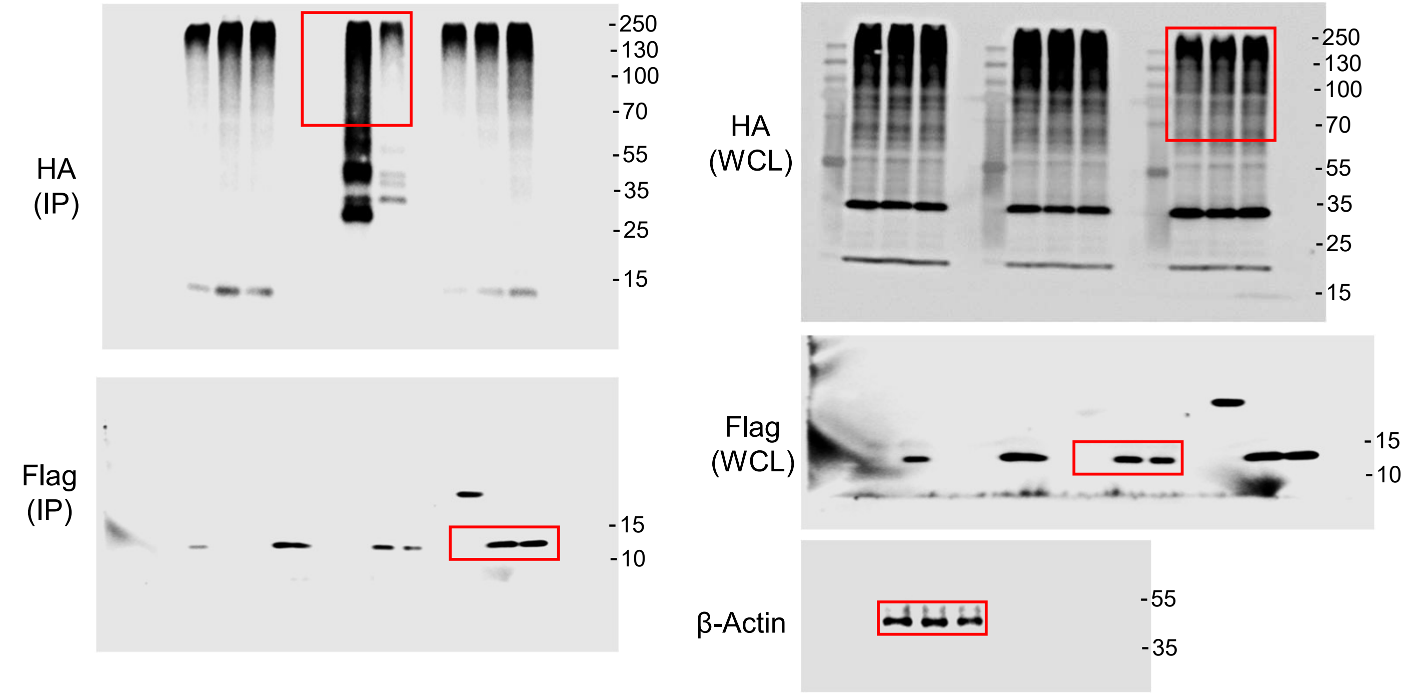

Fig. 1i

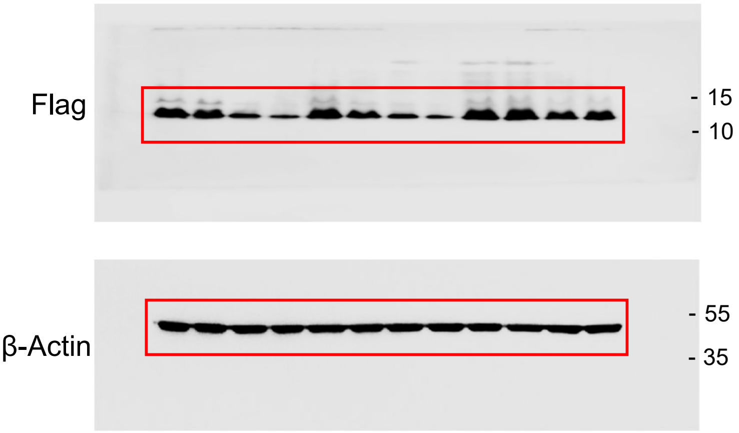

Fig. 1j

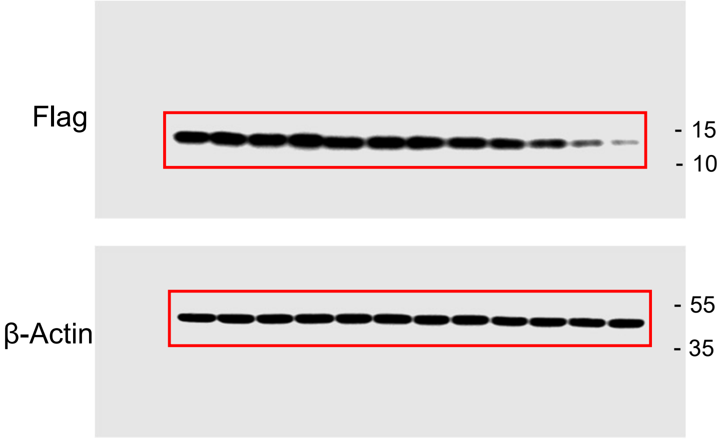

Fig. 2b

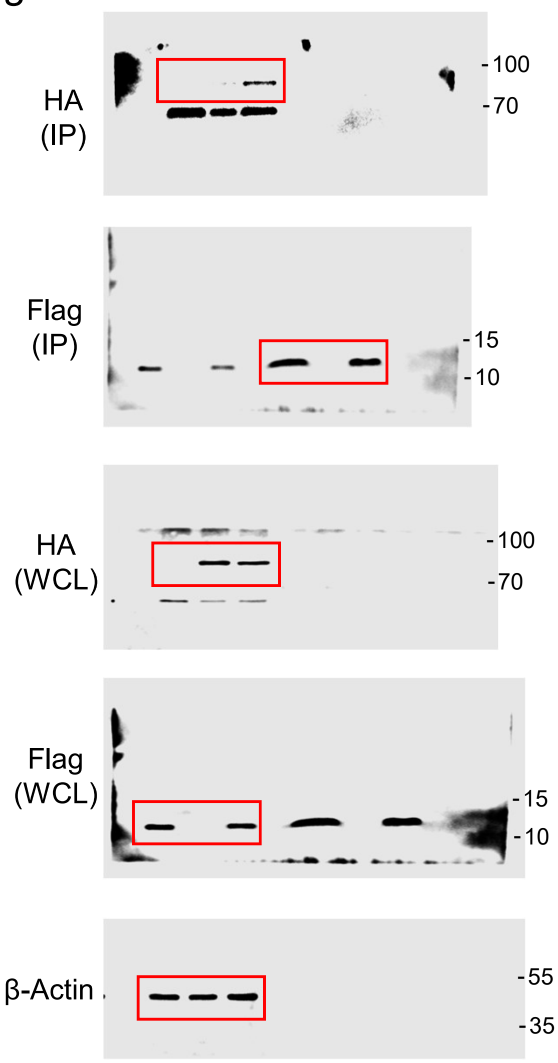

Fig. 2c

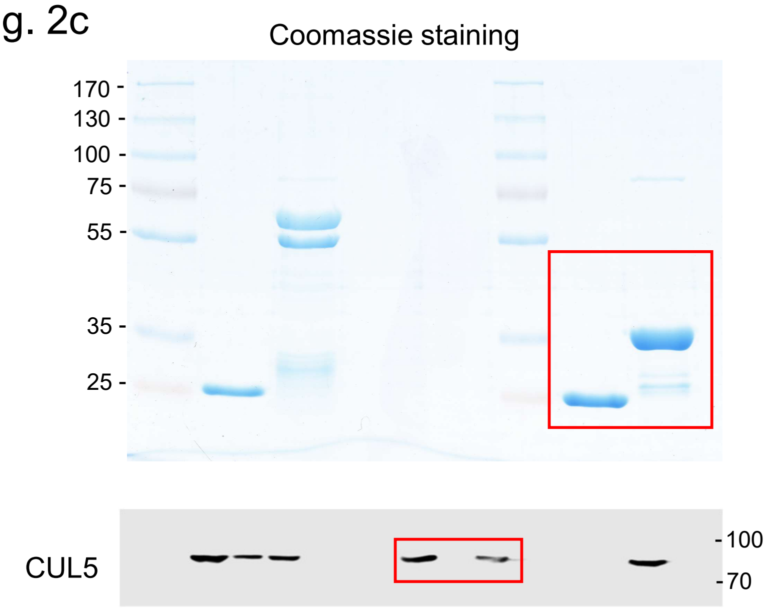

Fig. 2d

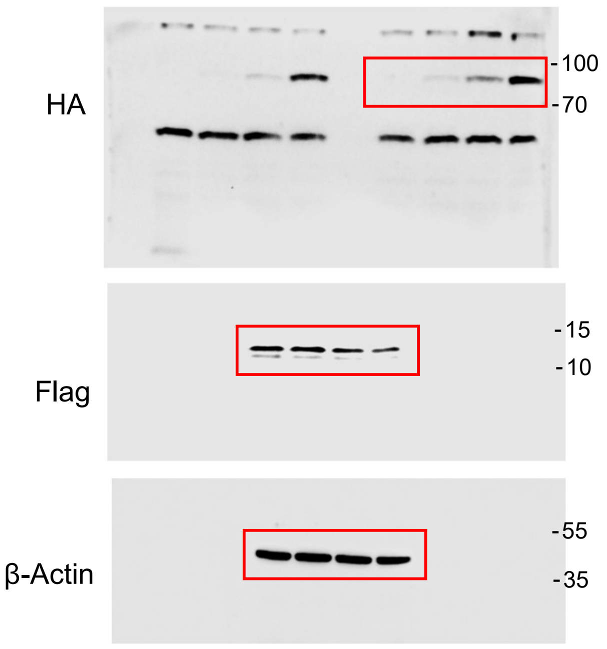

Fig. 2e

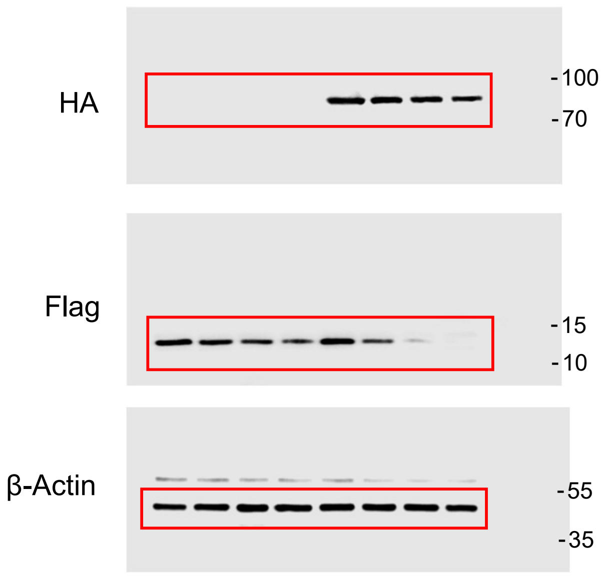

Fig. 2f

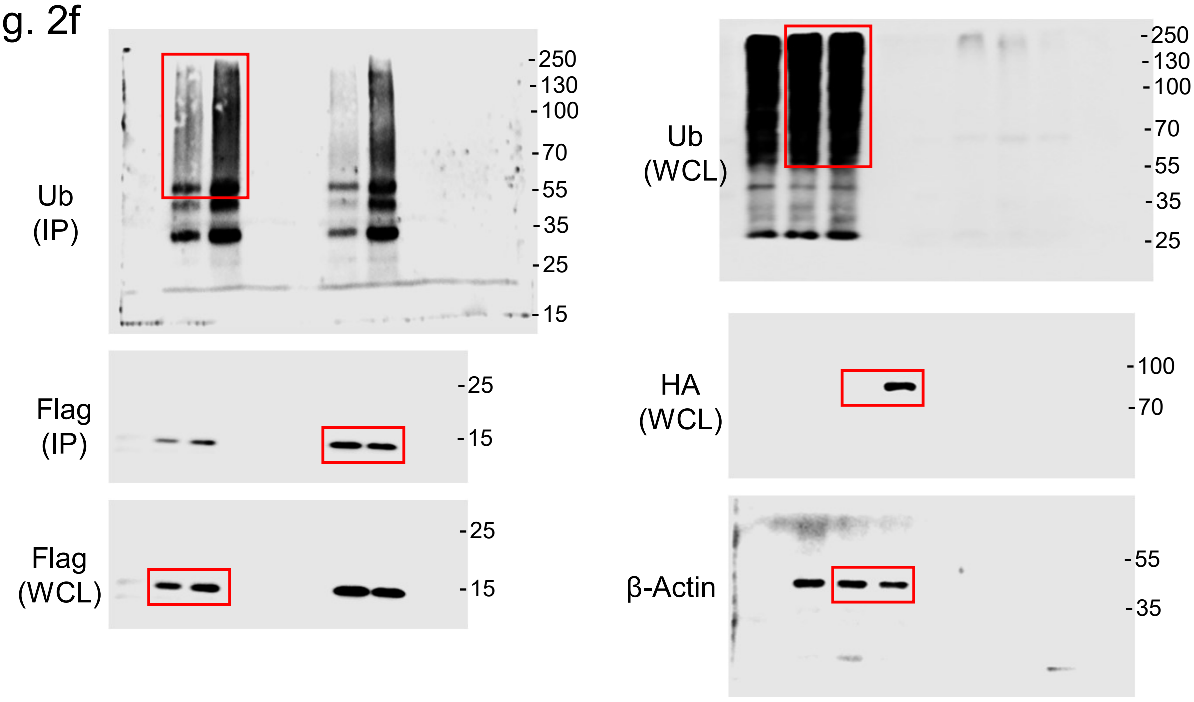

Fig. 2g

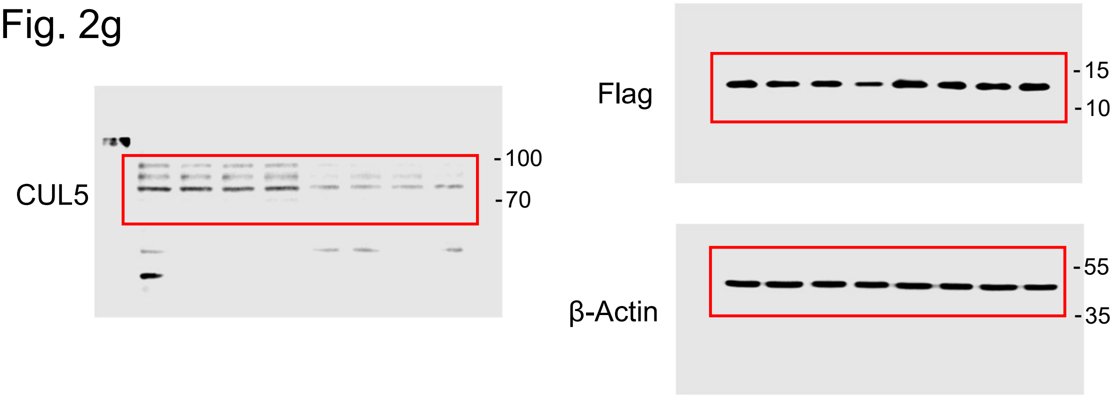

Fig. 2h

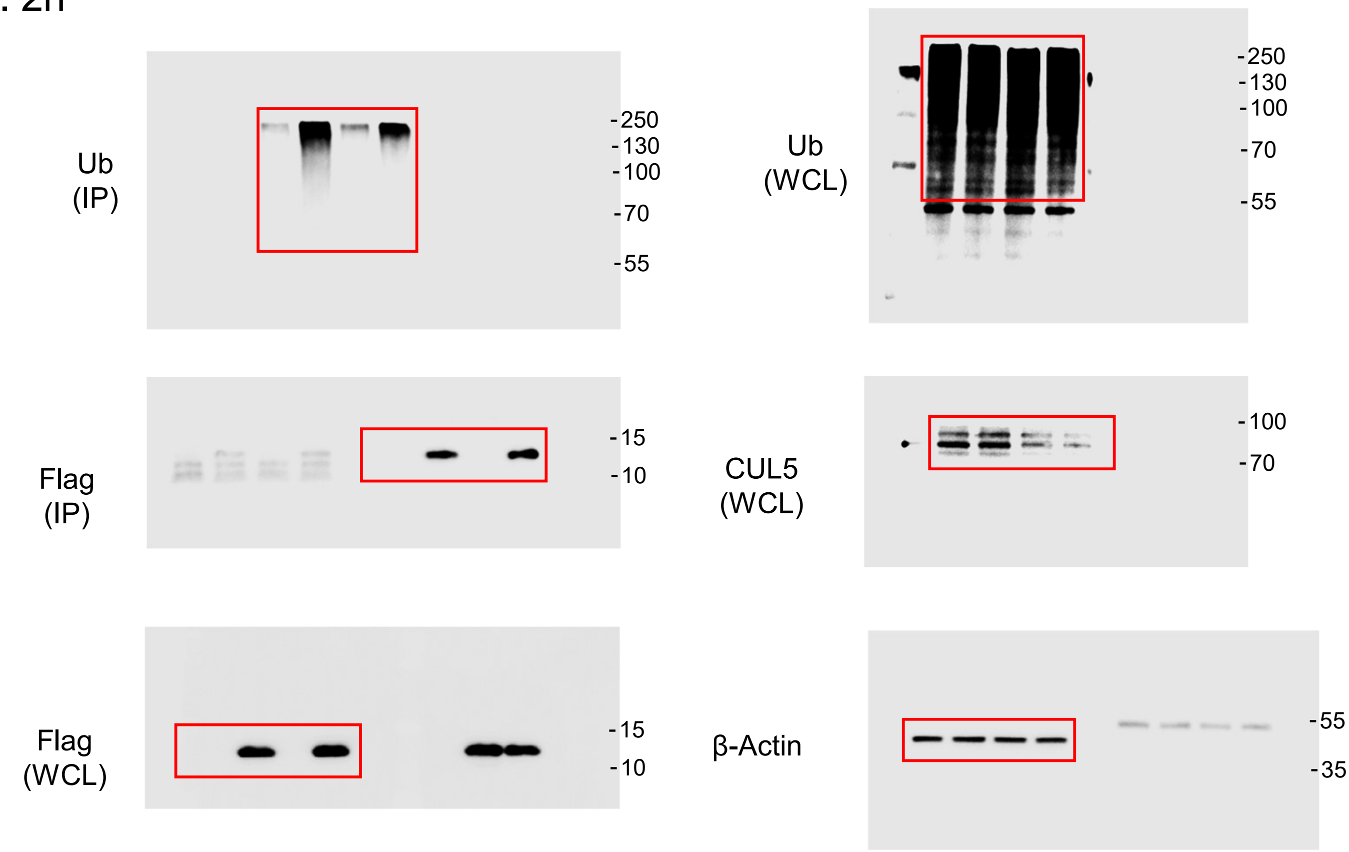

Fig. 3d

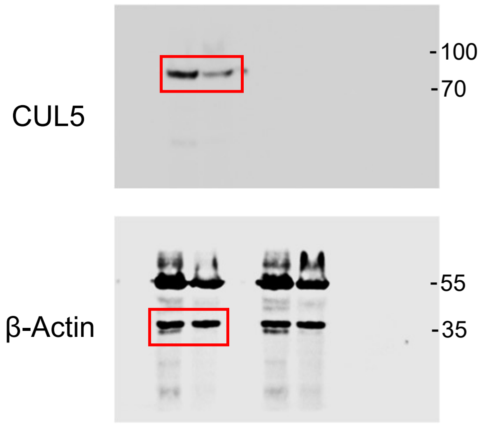

Fig. 3i

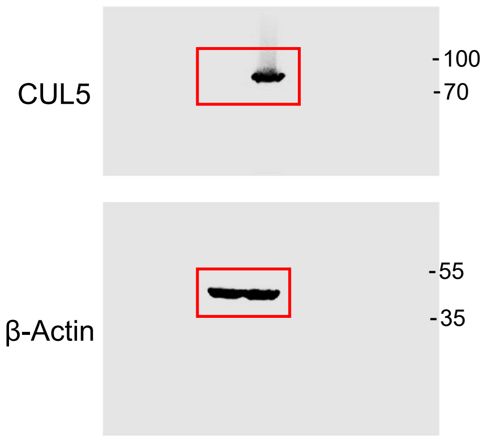

Fig. 3j

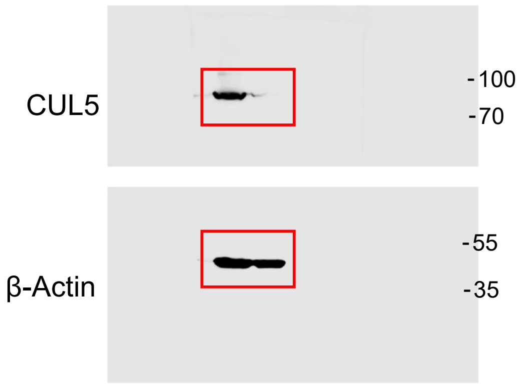

Fig. 3g

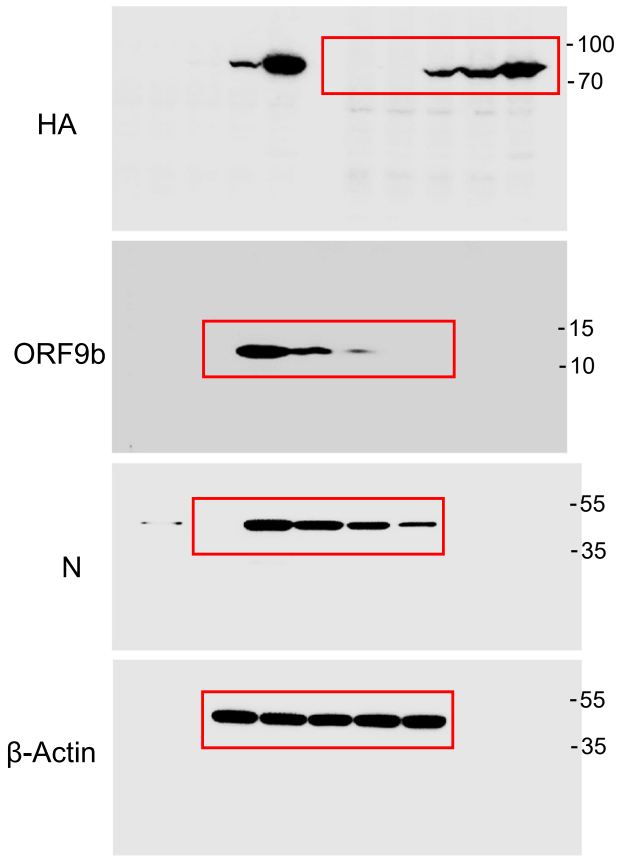

Fig. 3k

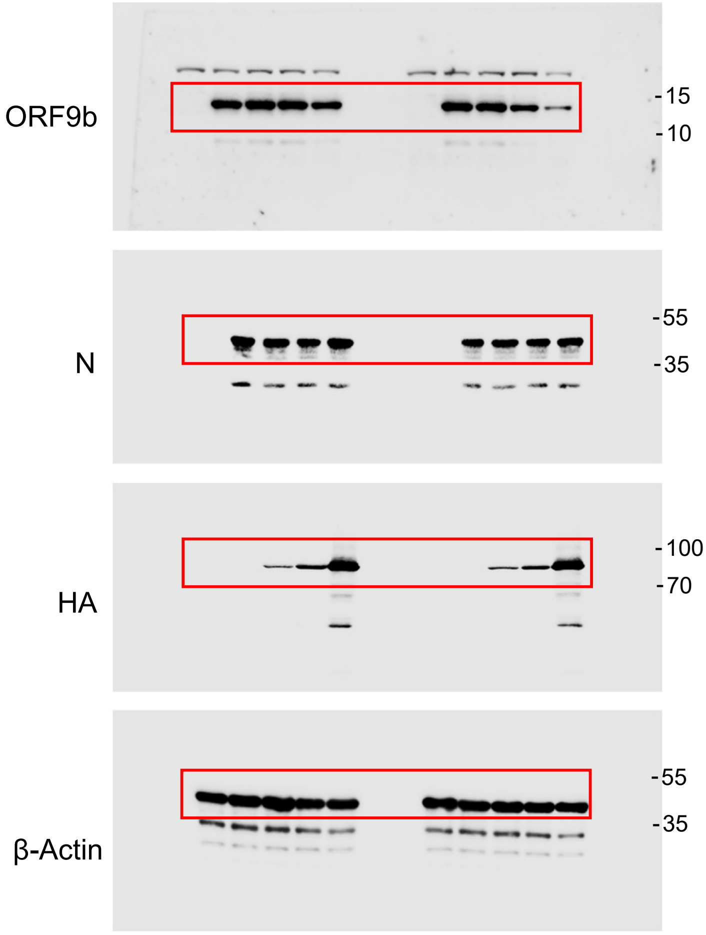

Fig. 4a

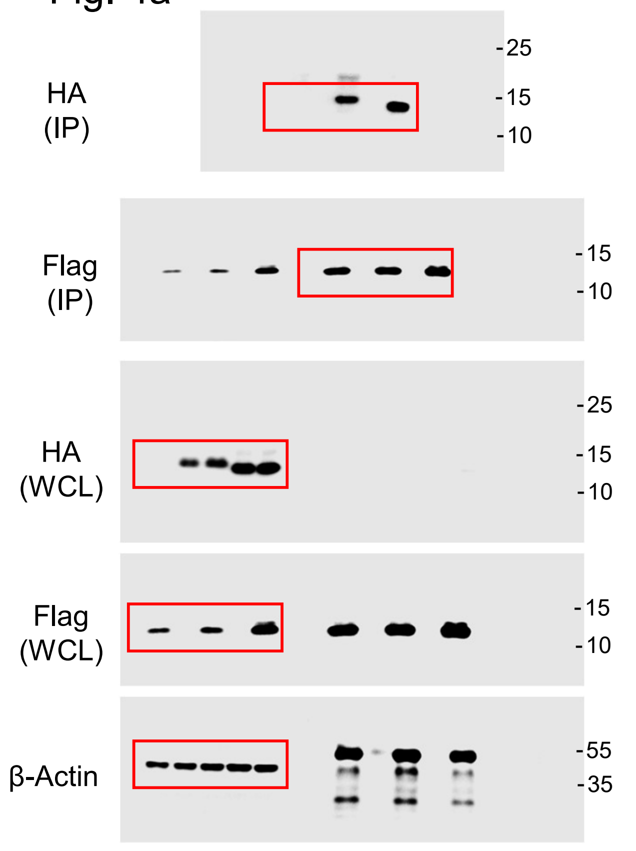

Fig. 4b

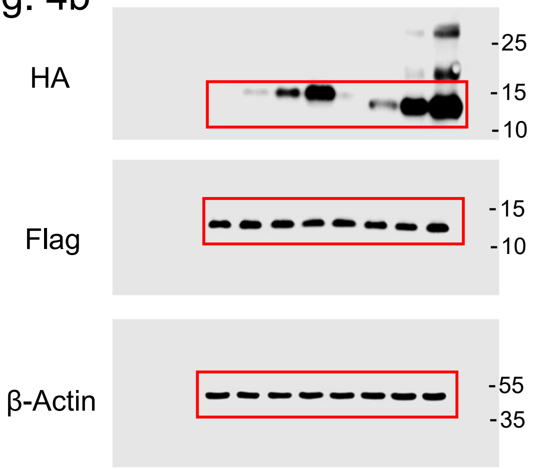

Fig. 4c

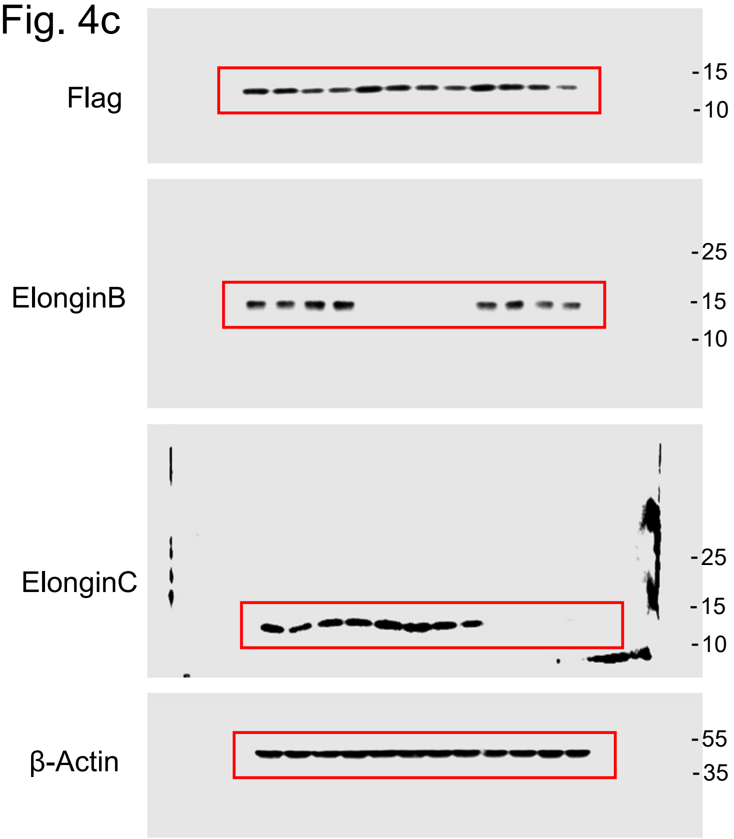

Fig. 4d

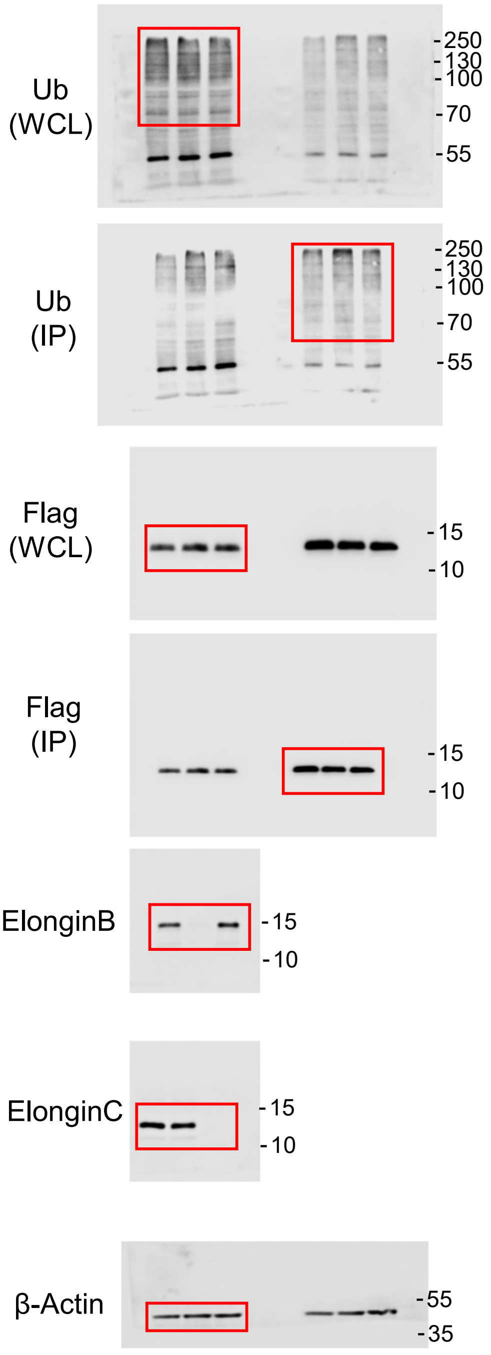

Fig. 4e

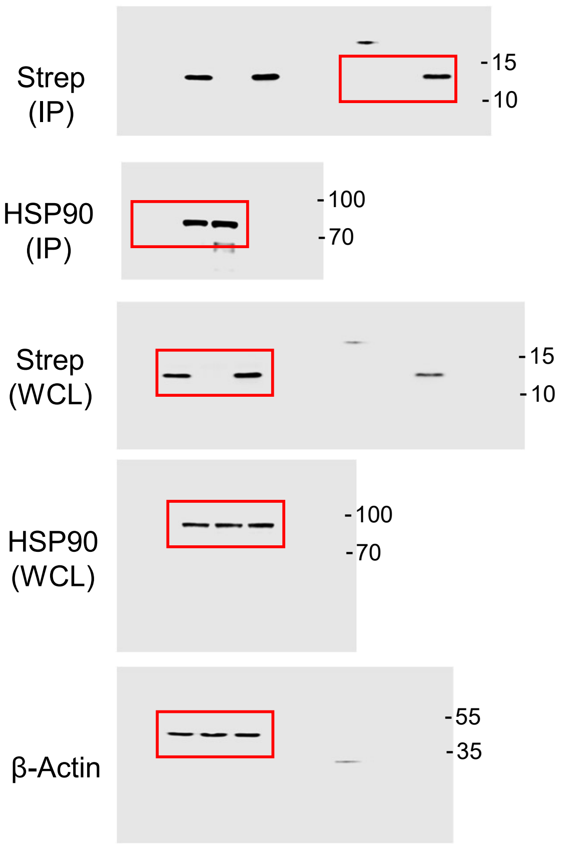

Fig. 4f

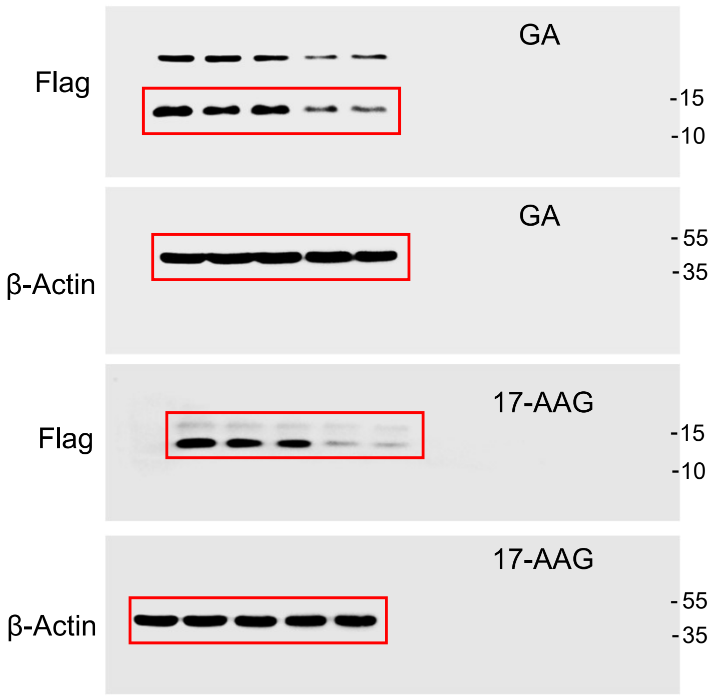

Fig. 4g

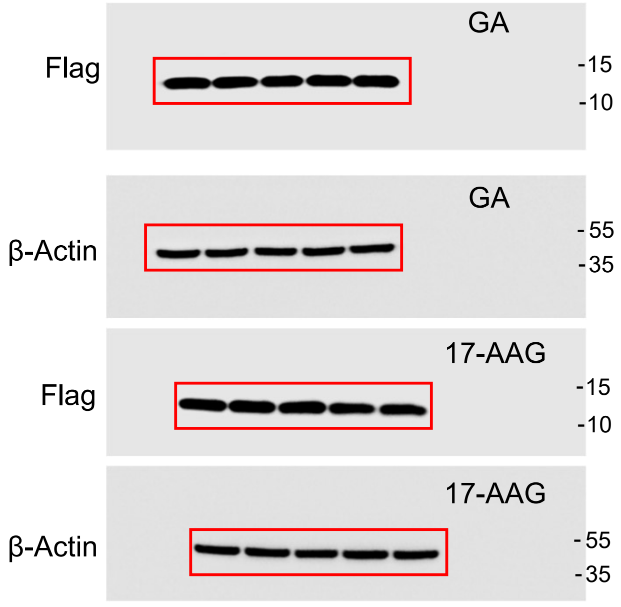

Fig. 4j

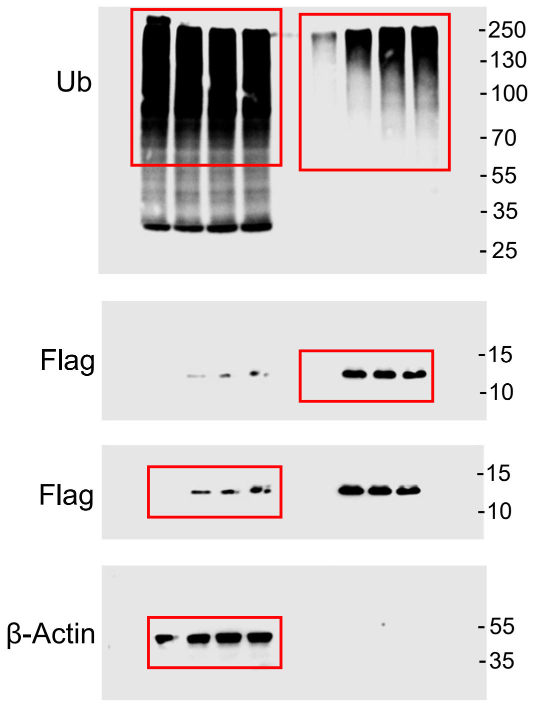

Fig. 4i

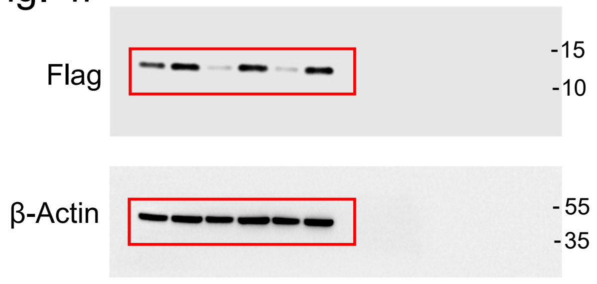

Fig. 4k

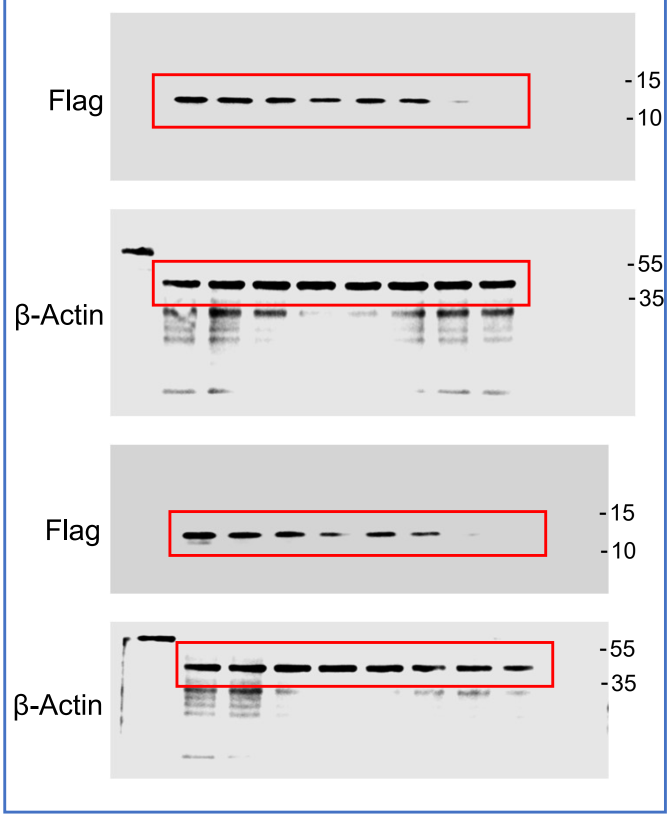

Fig. 4l

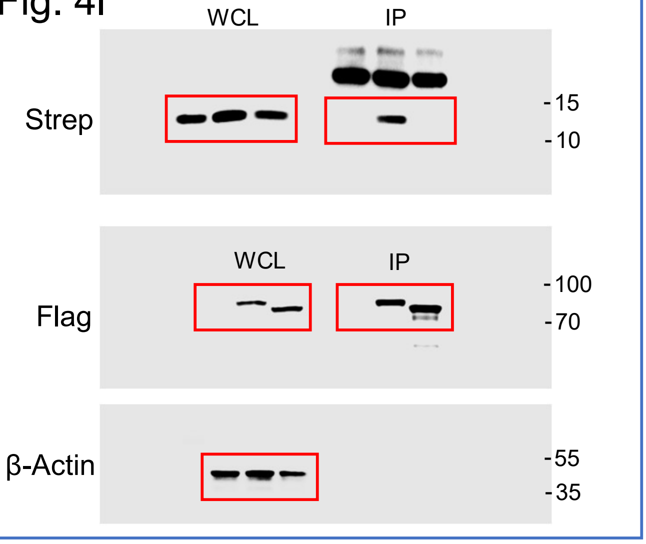

Fig. 4m

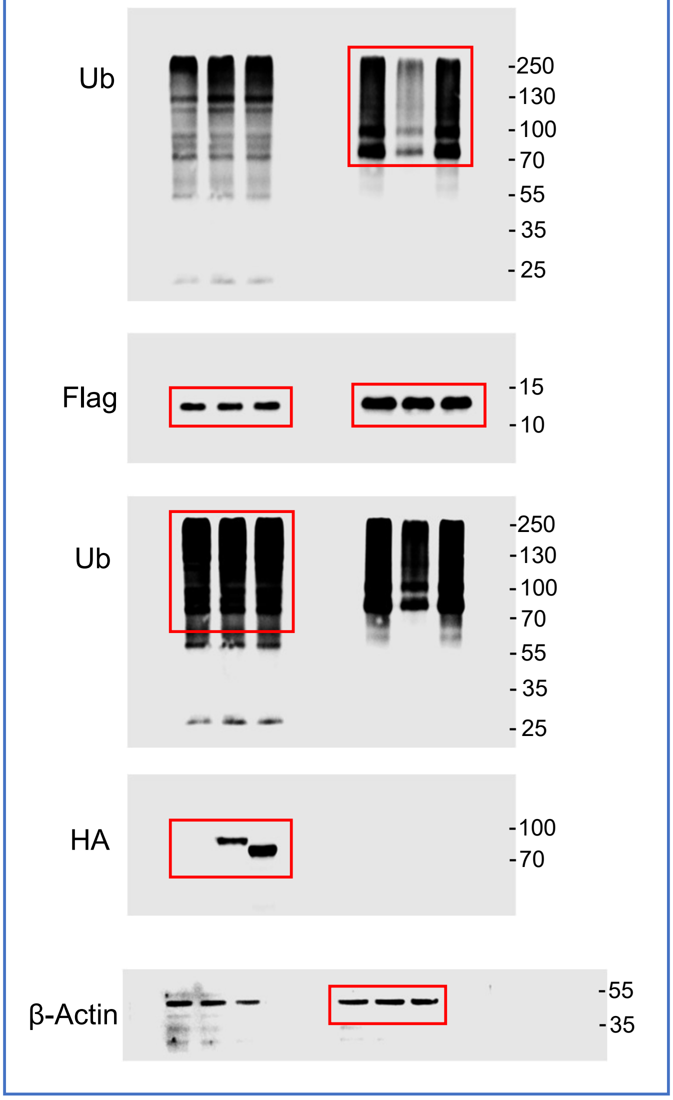

Fig. 4n

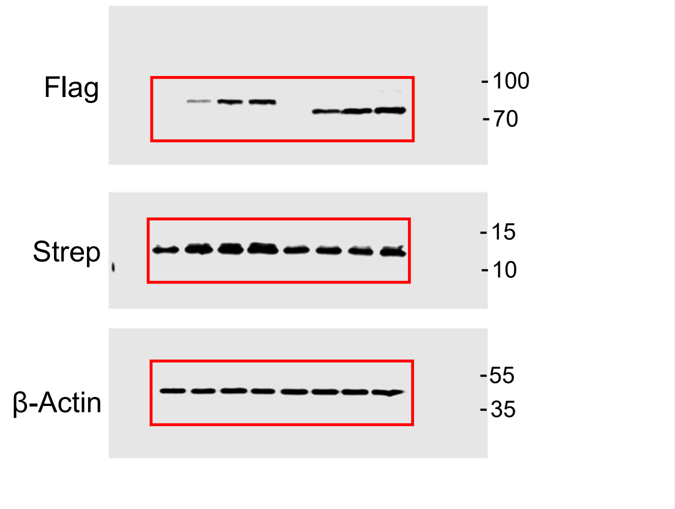

Fig. 4o

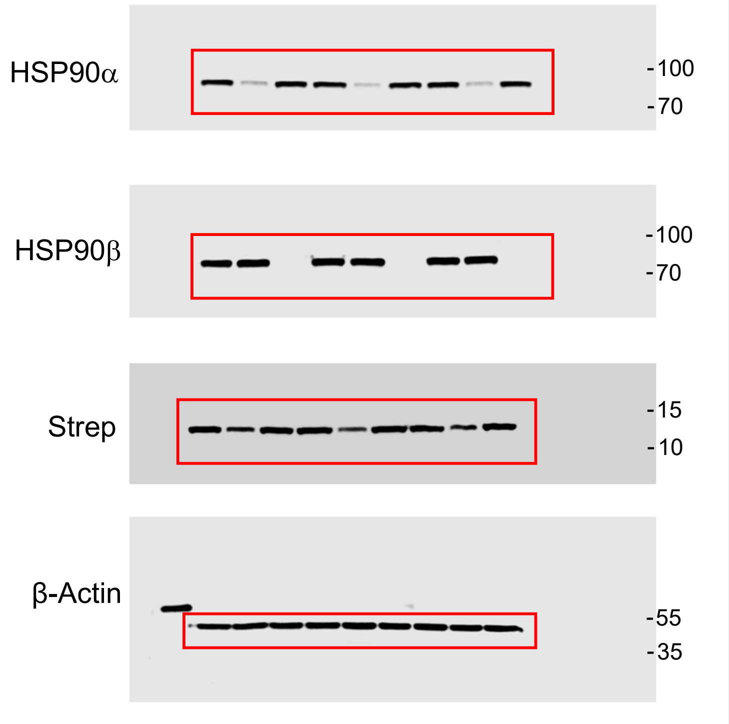

Fig. 5a

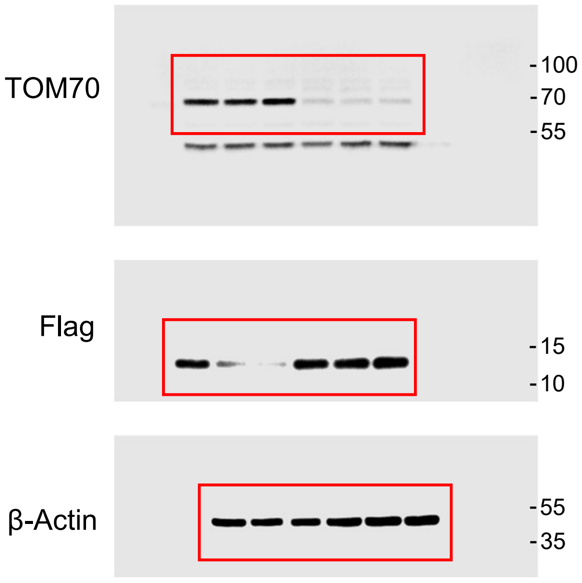

Fig. 5b

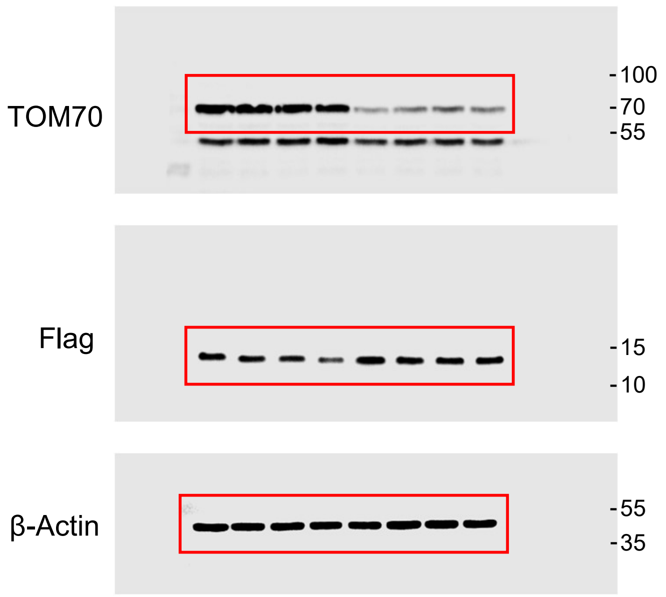

Fig. 5c

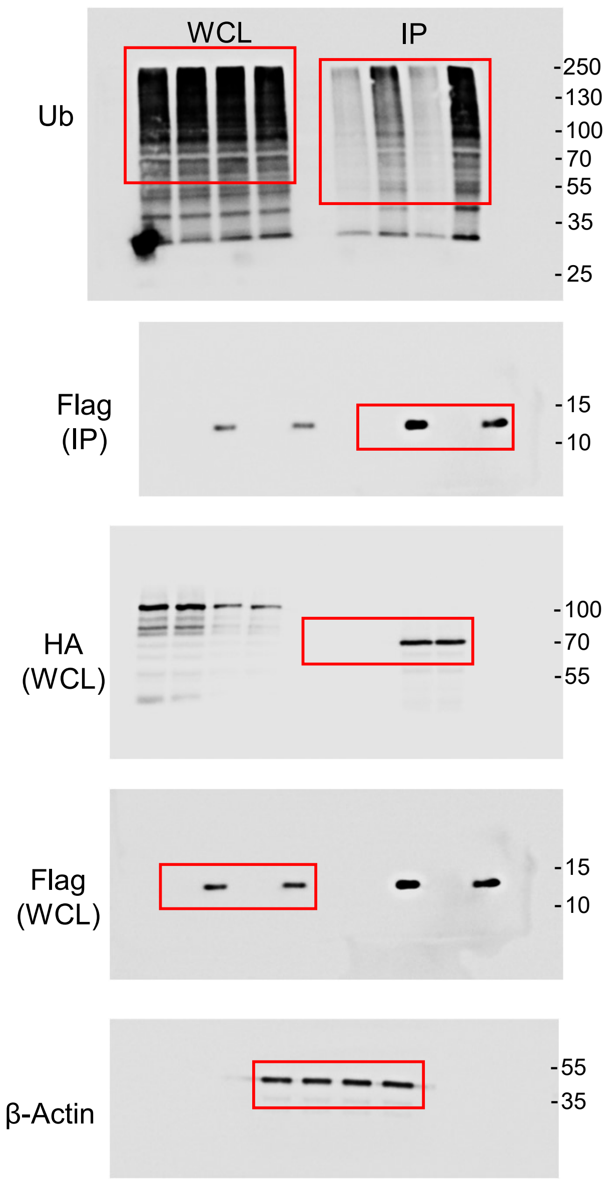

Fig. 5d

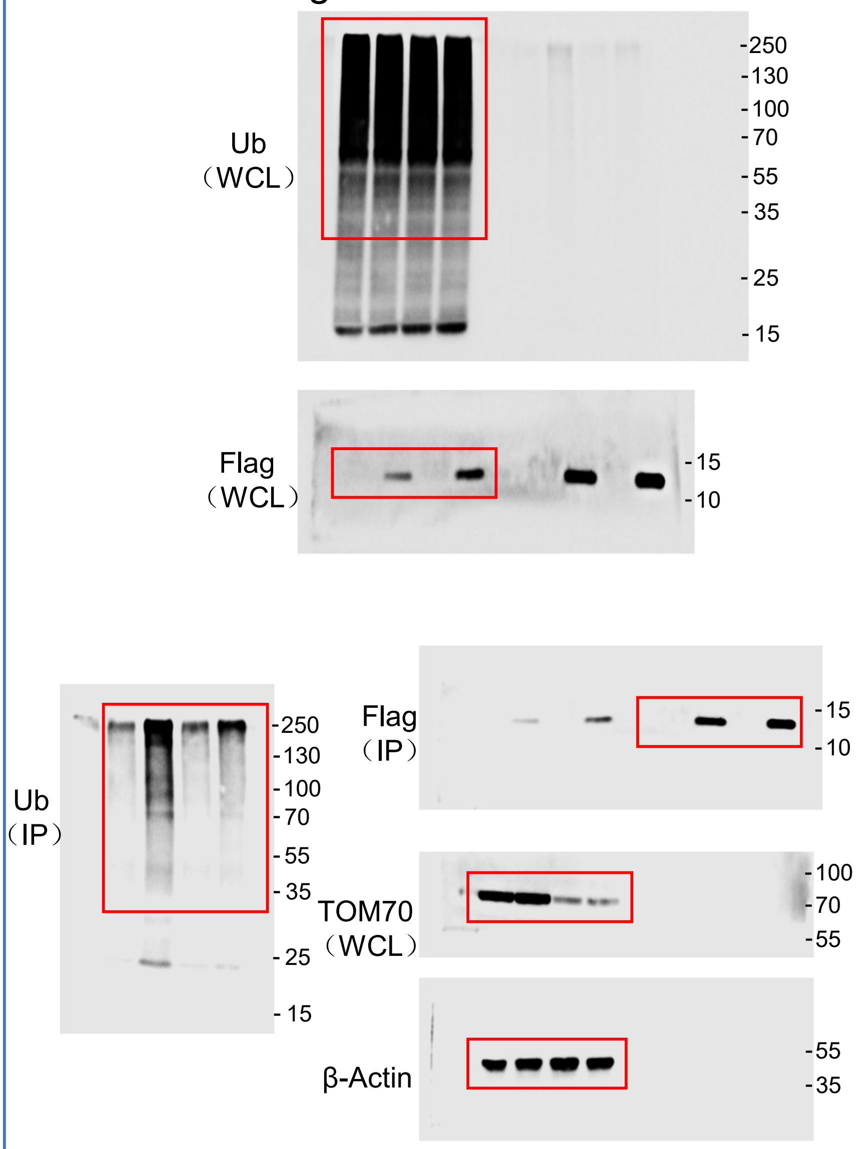

Fig. 5e

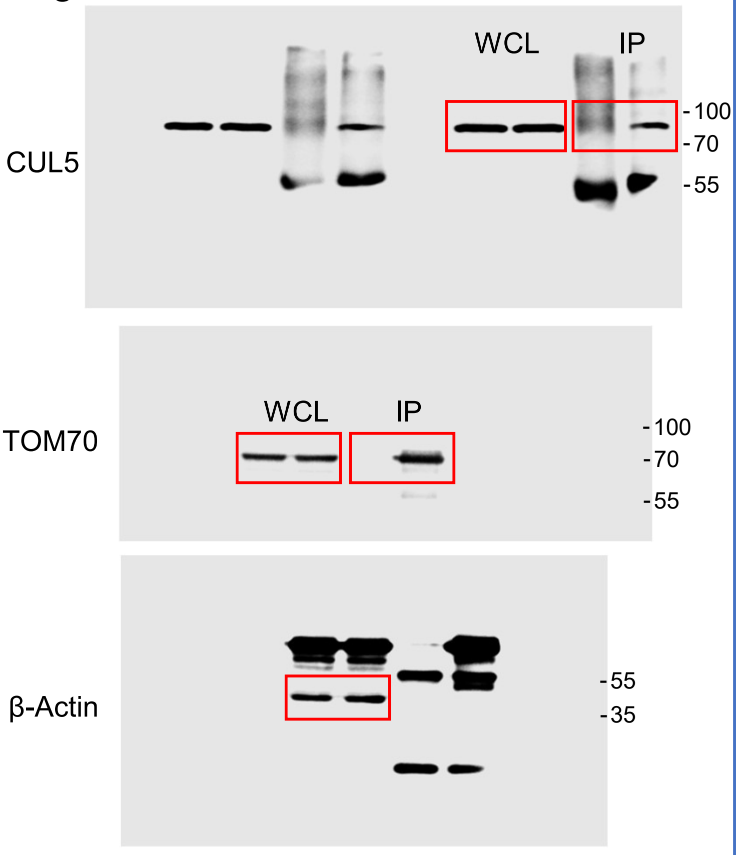

Fig. 5f

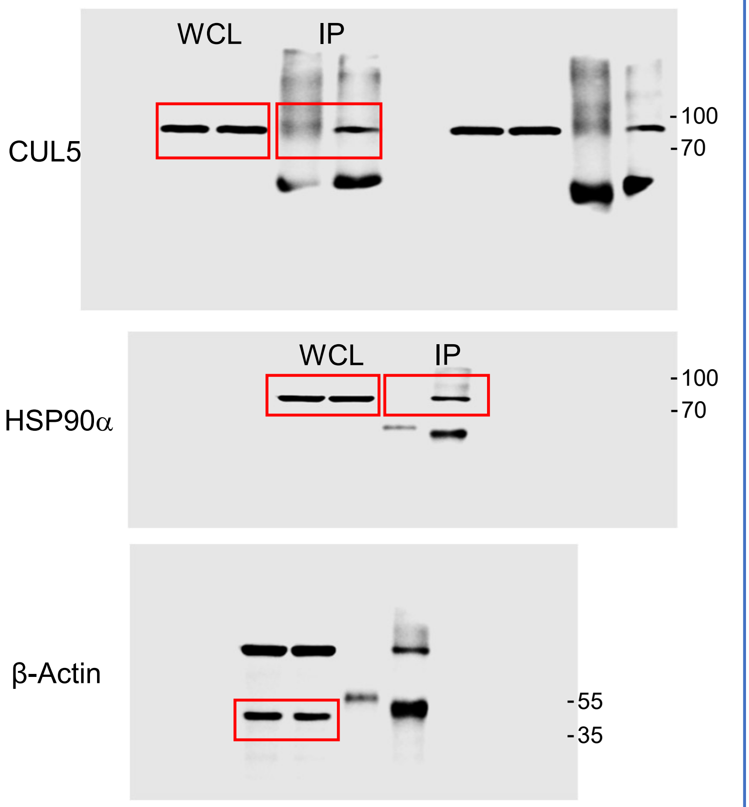

Fig. 5g

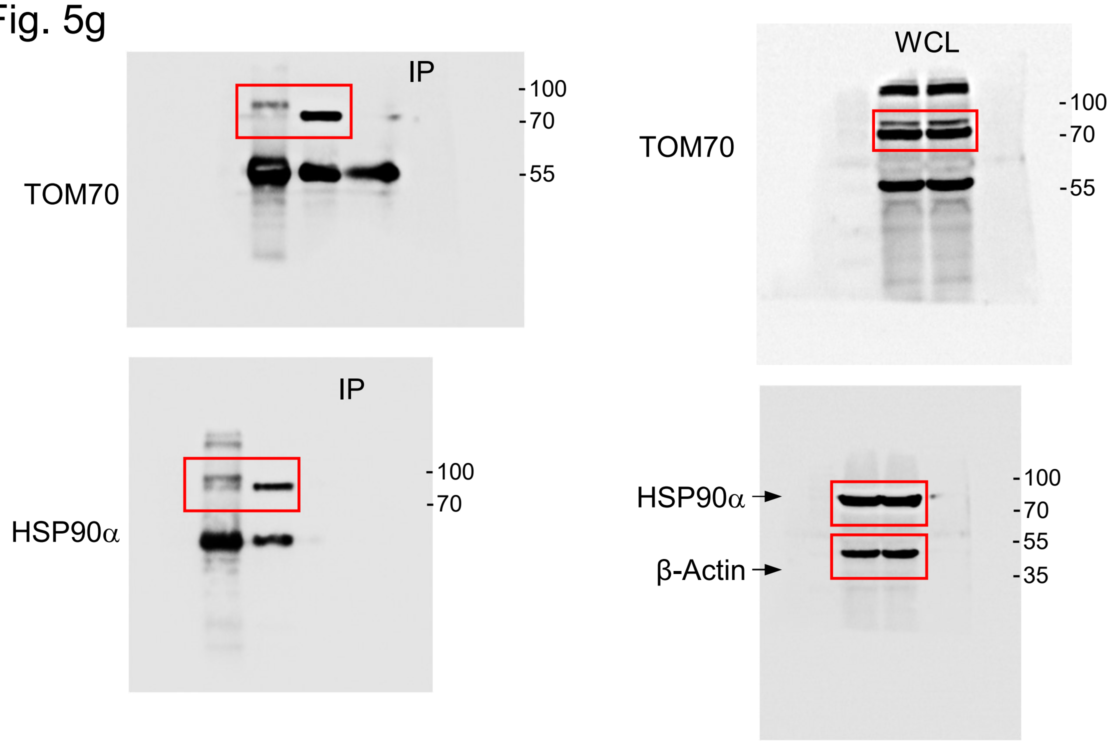

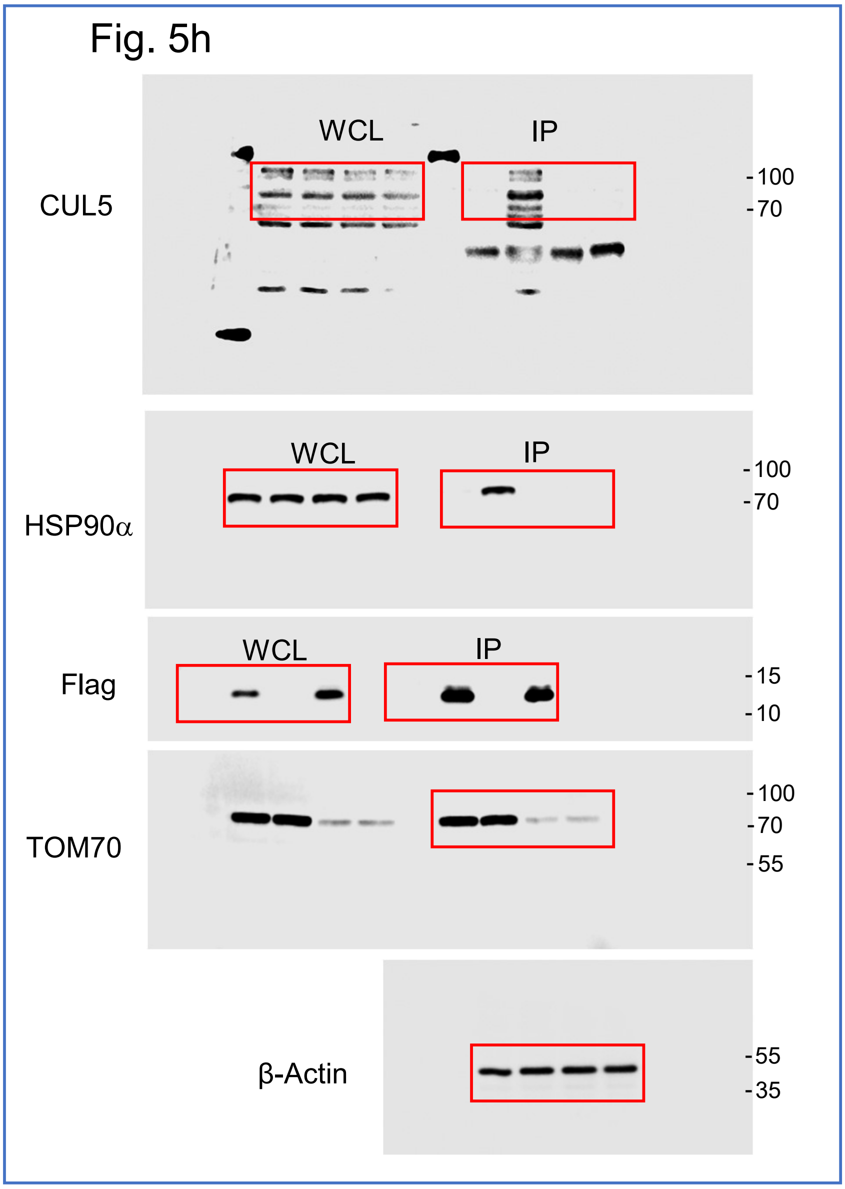
Fig. 5h
CUL5
WCL
IP
100
70
HSP90α
WCL
IP
100
70
Flag
WCL
IP
15
10
TOM70
100
70
55
β-Actin
55
35

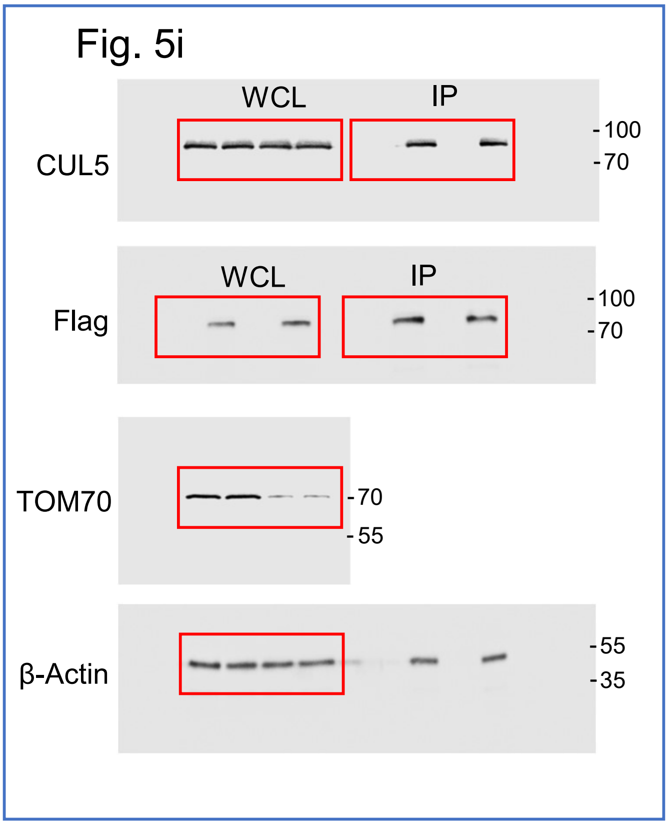
Fig. 5i
CUL5
WCL
IP
100
70
Flag
WCL
IP
100
70
TOM70
70
55
β-Actin
55
35

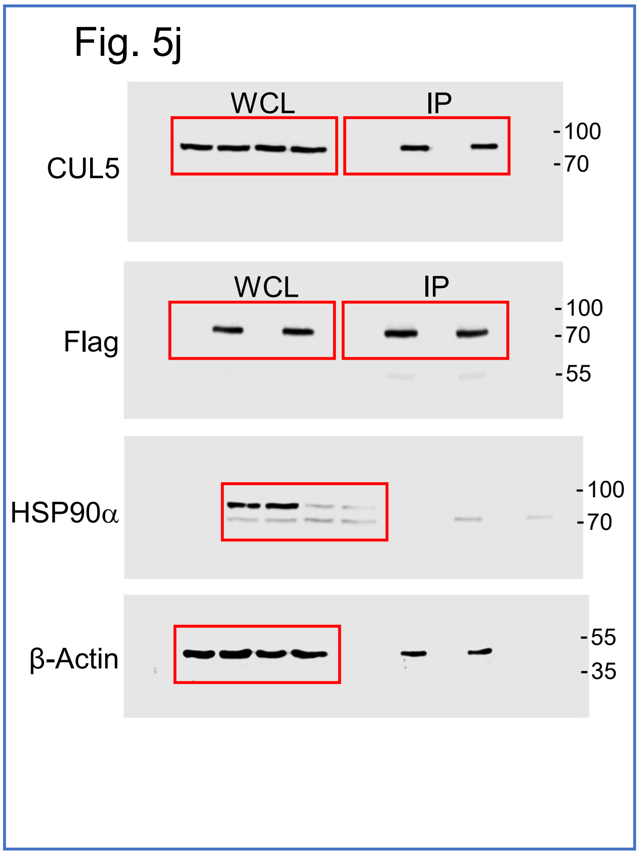
Fig. 5j
CUL5
WCL
IP
100
70
Flag
WCL
IP
100
70
55
HSP90α
100
70
β-Actin
55
35

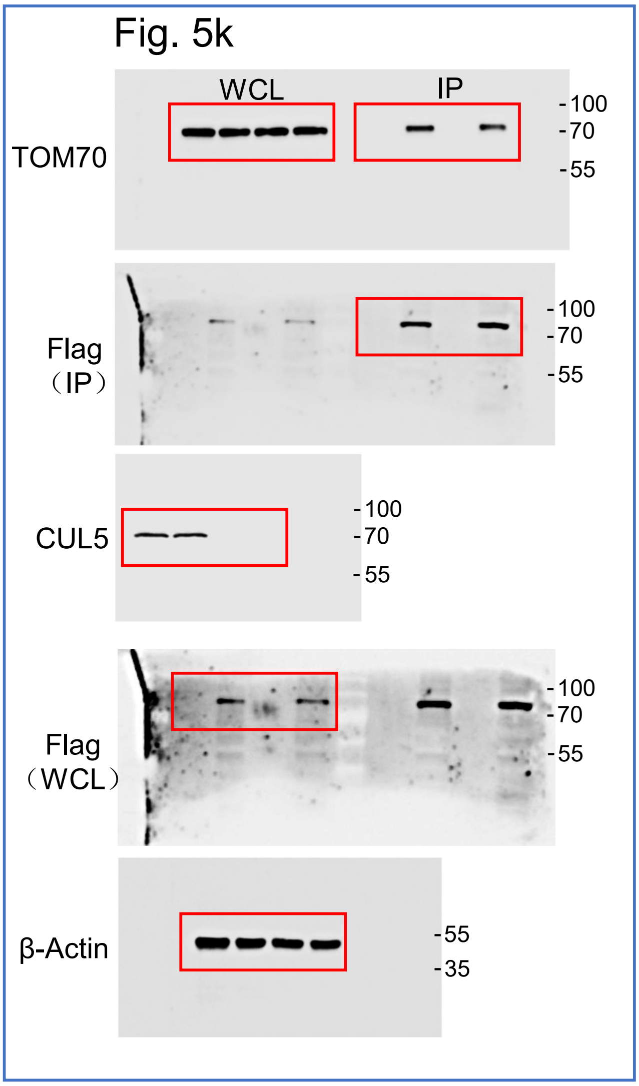
Fig. 5k
TOM70
WCL
IP
100
70
55
Flag (IP)
100
70
55
CUL5
100
70
55
Flag (WCL)
100
70
55
β-Actin
55
35

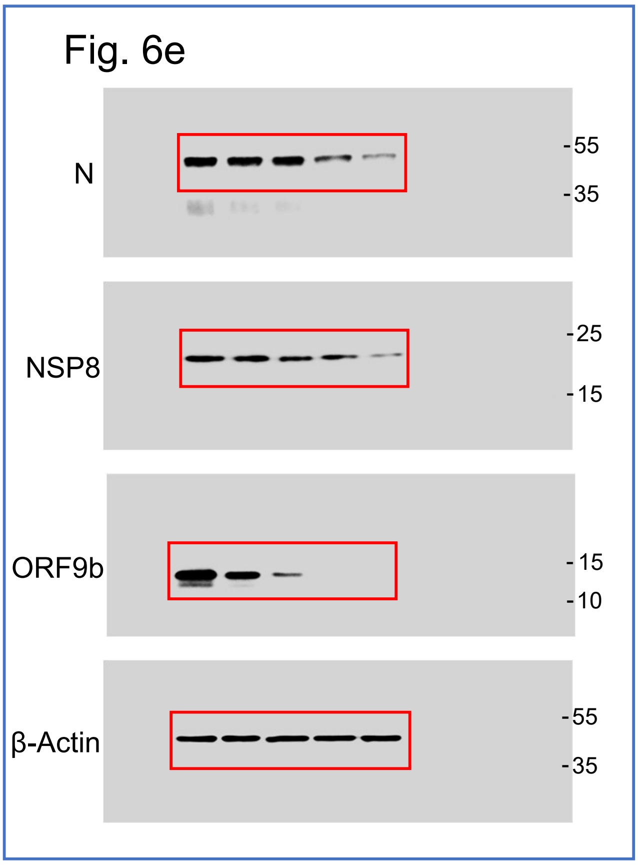
Fig. 6e
N
NSP8
ORF9b
β-Actin
55
35
25
15
15
10
55
35

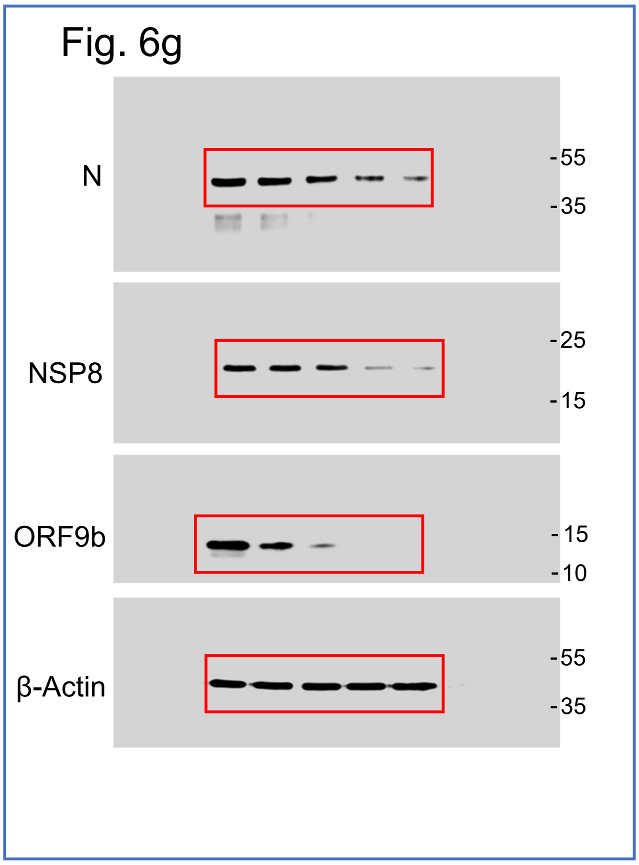
Fig. 6g
N
NSP8
ORF9b
β-Actin
55
35
25
15
15
10
55
35

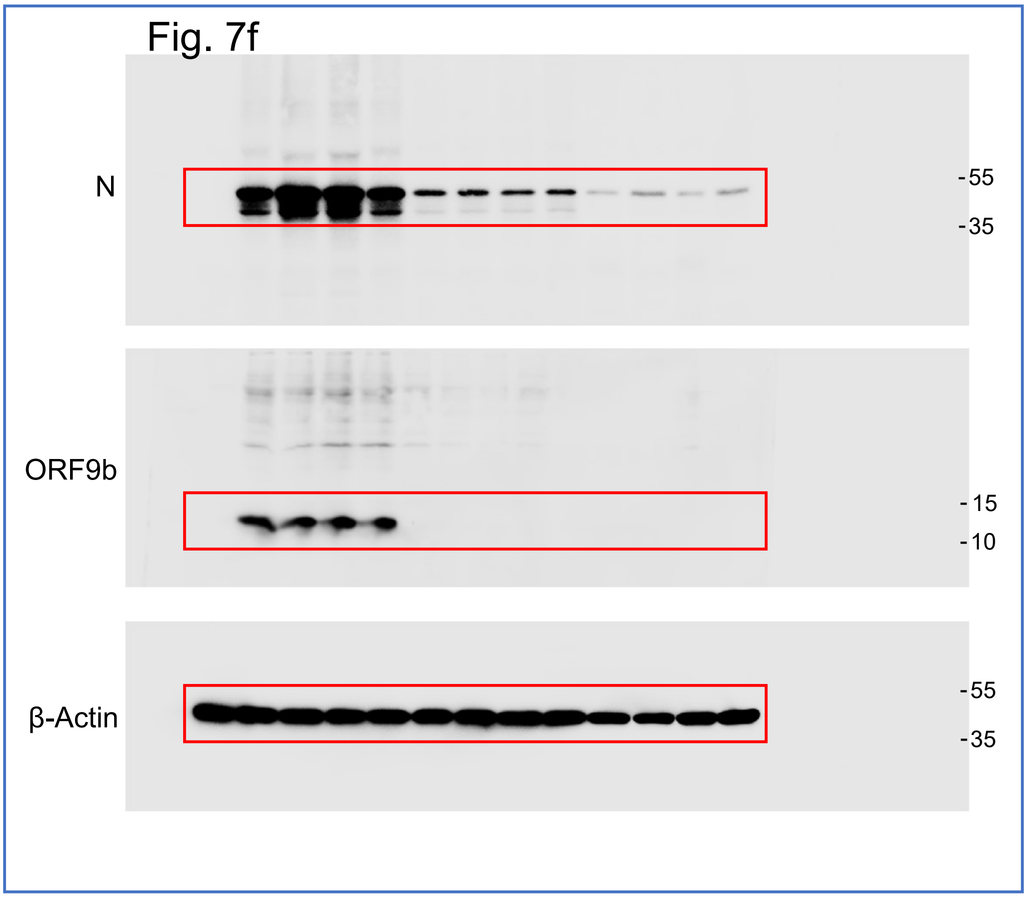
Fig. 7f
N
ORF9b
β-Actin
55
35
15
10
55
35

Fig. S2a

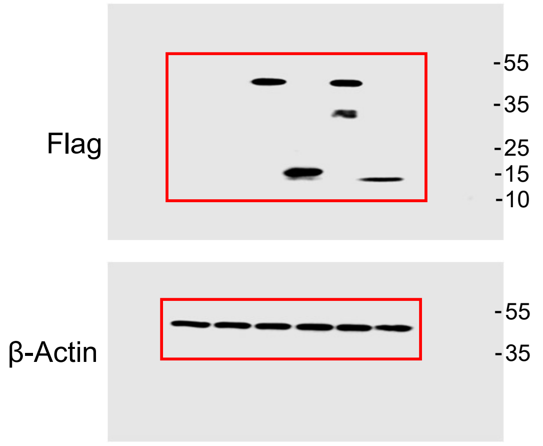

Fig. S3a

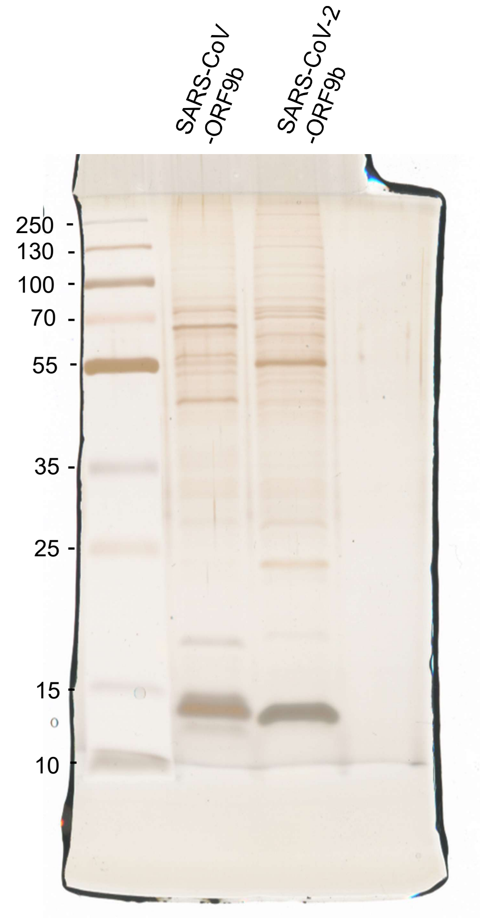

Fig. S4a

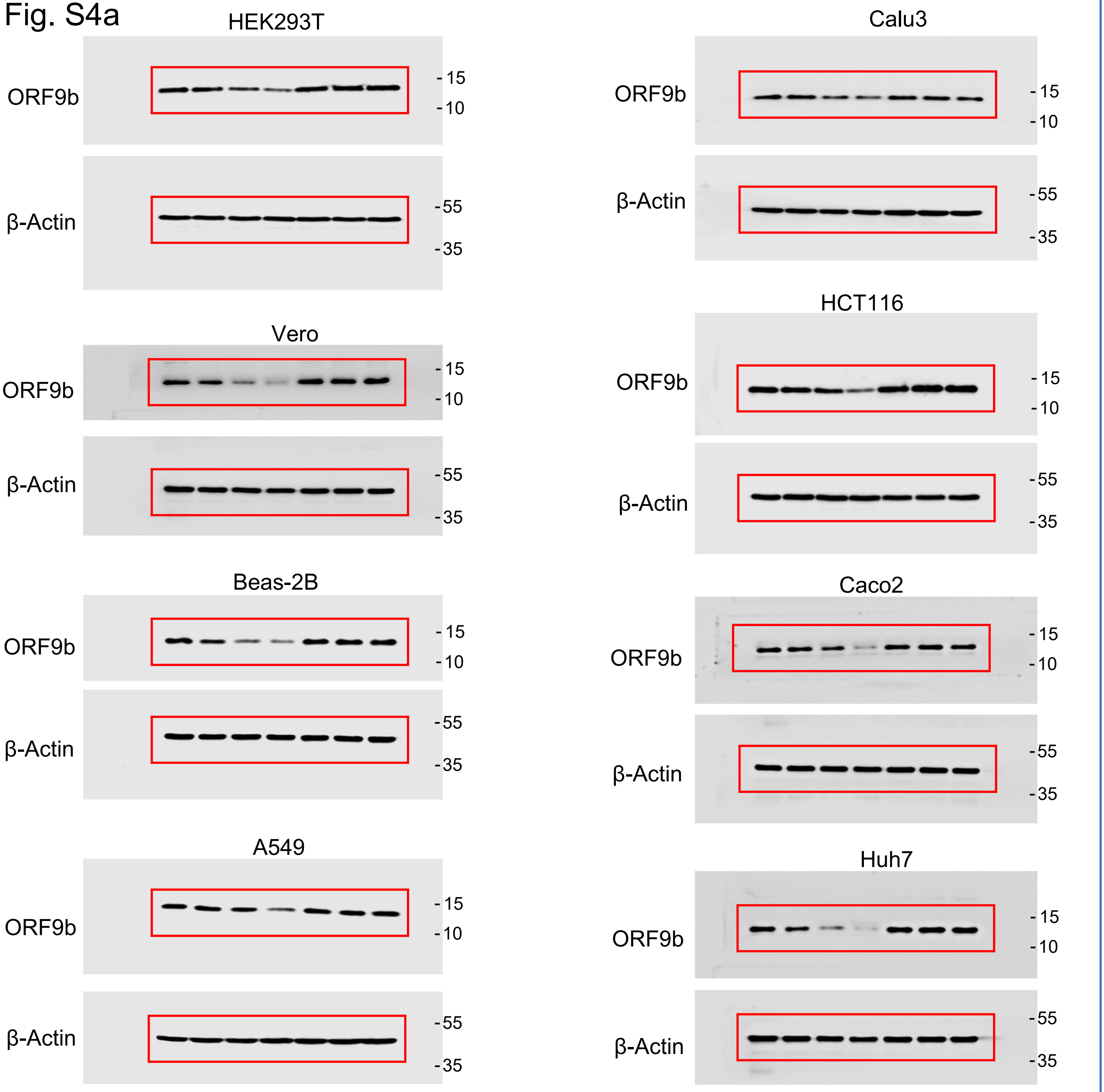
HEK293T
ORF9b
15
10
β-Actin
55
35
Calu3
ORF9b
15
10
β-Actin
55
35
Vero
ORF9b
15
10
β-Actin
55
35
HCT116
ORF9b
15
10
β-Actin
55
35
Beas-2B
ORF9b
15
10
β-Actin
55
35
Caco2
ORF9b
15
10
β-Actin
55
35
A549
ORF9b
15
10
β-Actin
55
35
Huh7
ORF9b
15
10
β-Actin
55
35

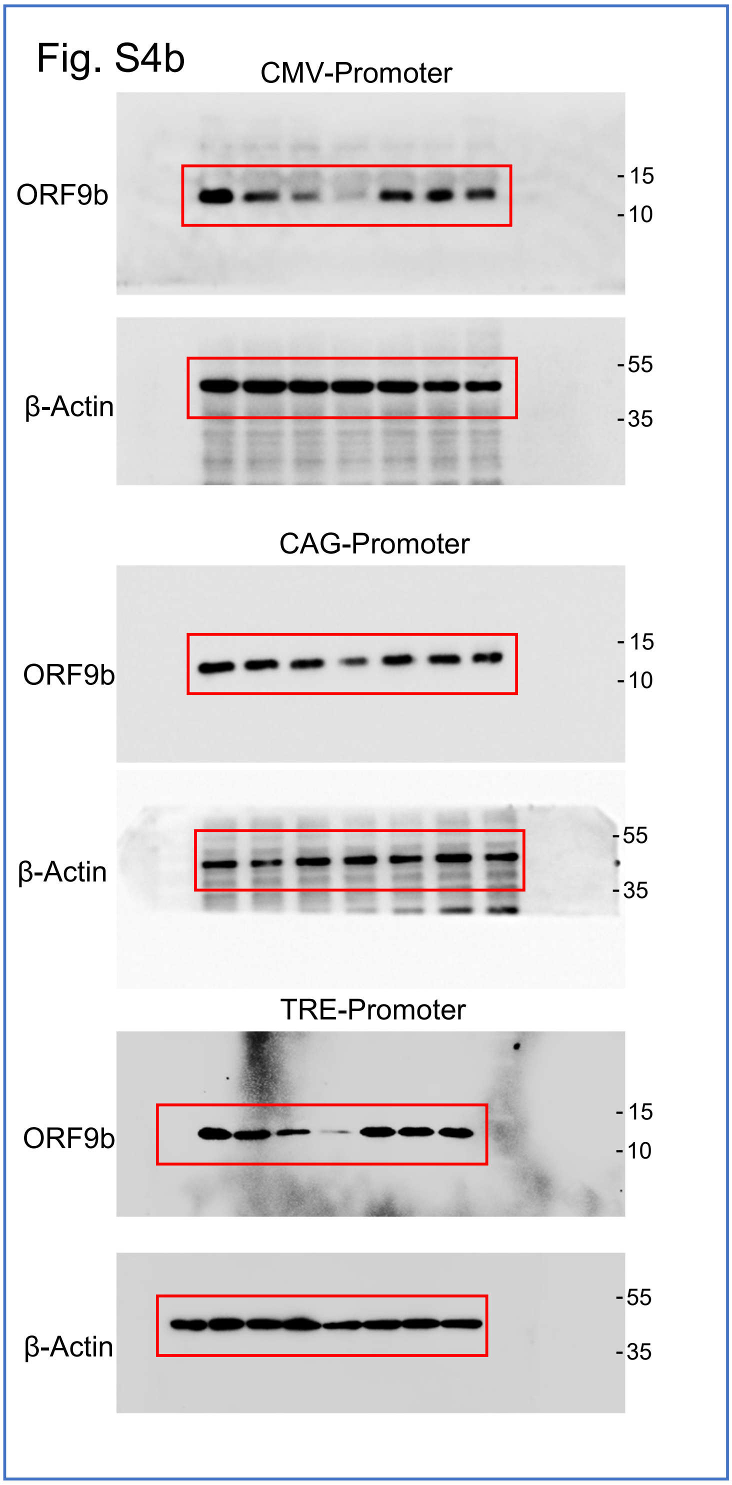
Fig. S4b
CMV-Promoter
ORF9b
15
10
β-Actin
55
35
CAG-Promoter
ORF9b
15
10
β-Actin
55
35
TRE-Promoter
ORF9b
15
10
β-Actin
55
35

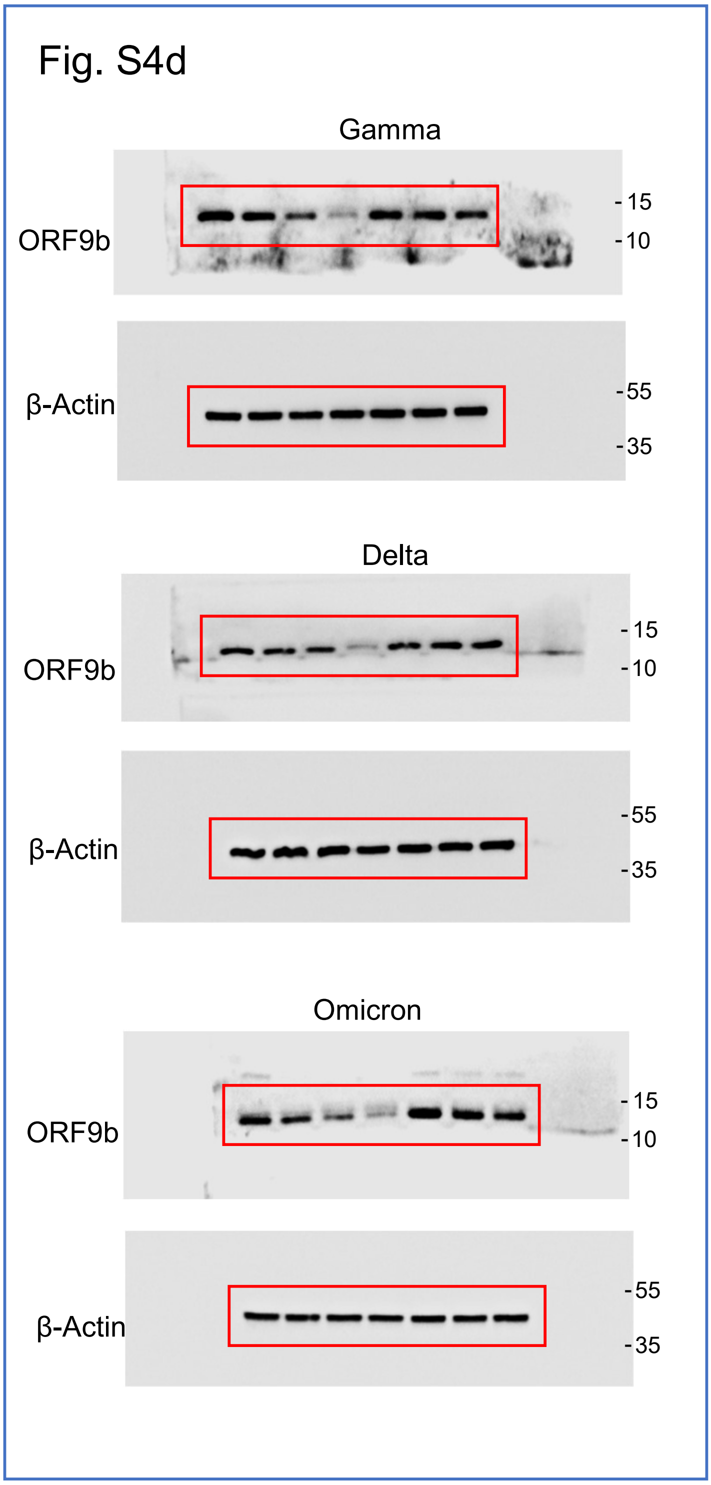
Fig. S4d
Gamma
ORF9b
15
10
β-Actin
55
35
Delta
ORF9b
15
10
β-Actin
55
35
Omicron
ORF9b
15
10
β-Actin
55
35

Fig. S5a

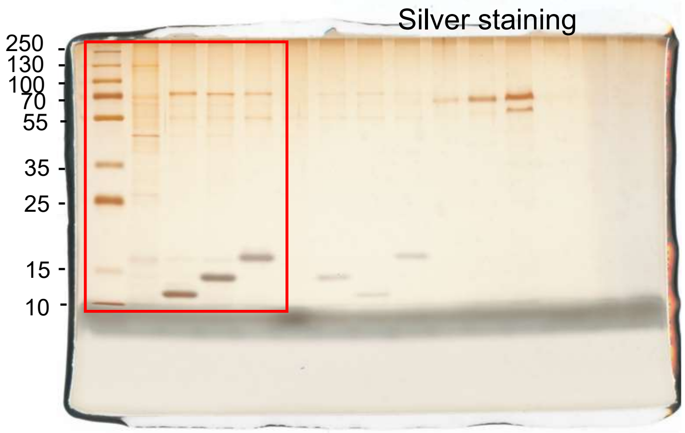

Fig. S5e

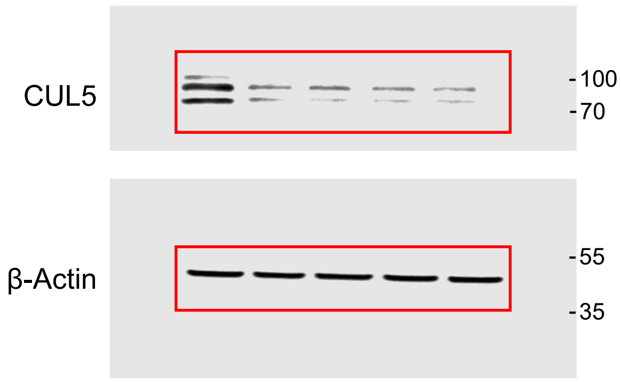

Fig. S6a

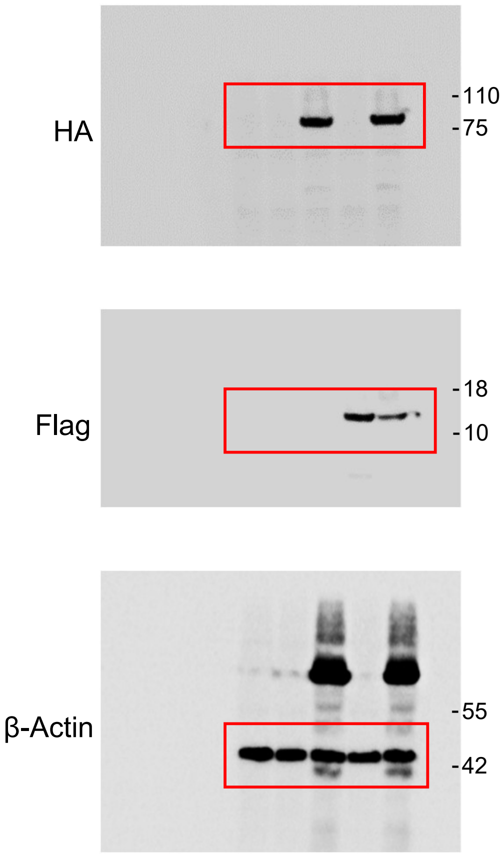

Fig. S6b

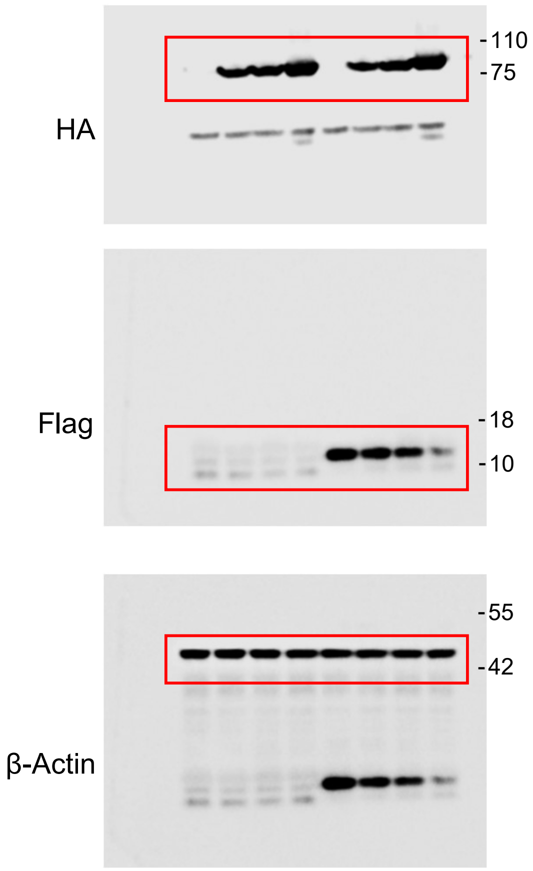

Fig. S7a

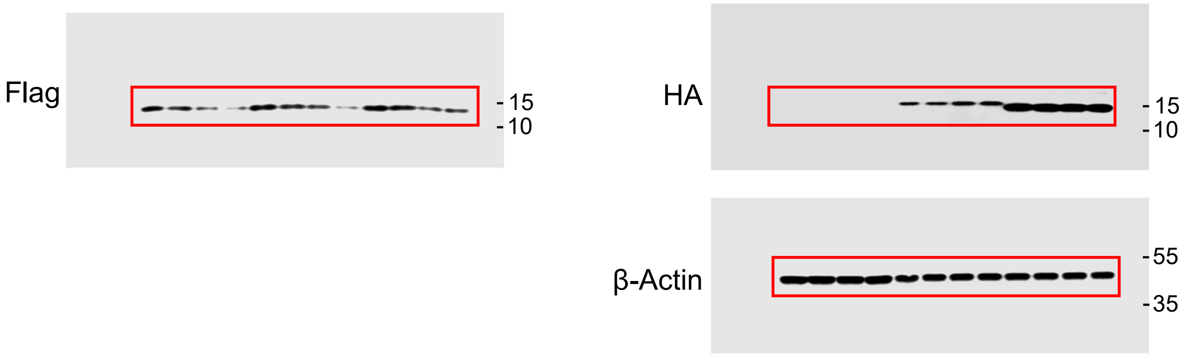

Fig. S7c

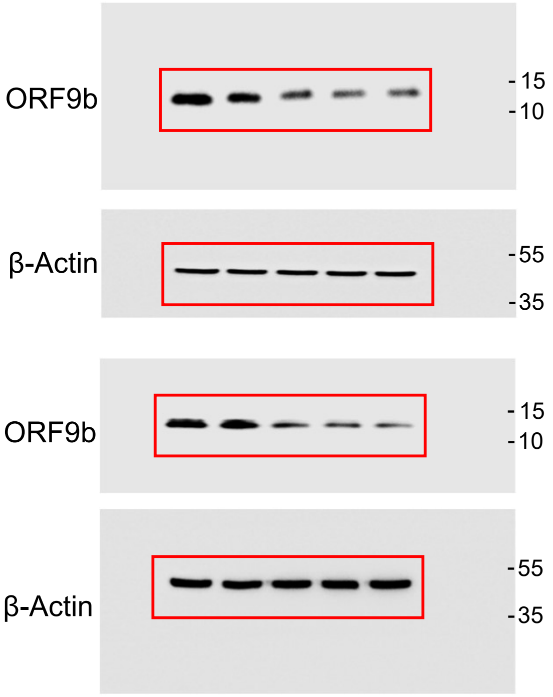

Fig. S7e

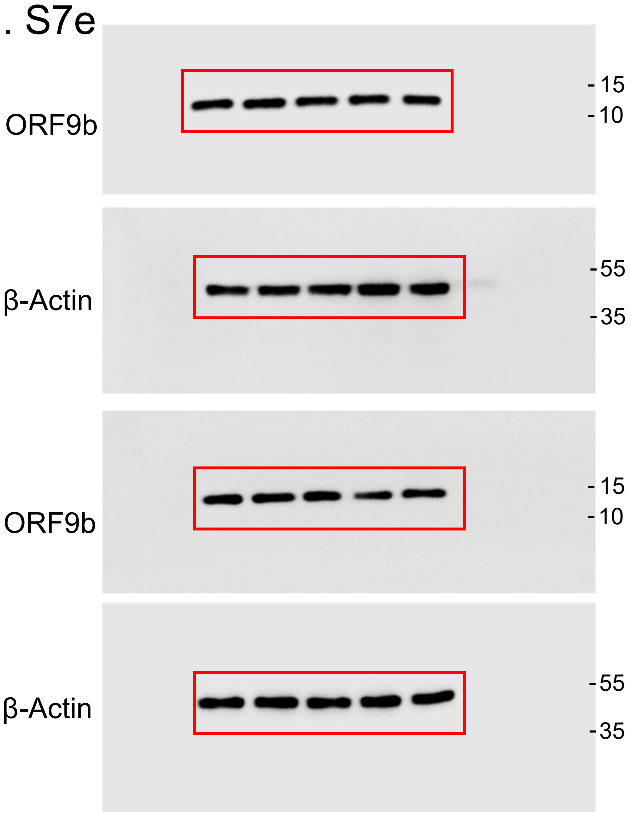

Fig. S7d

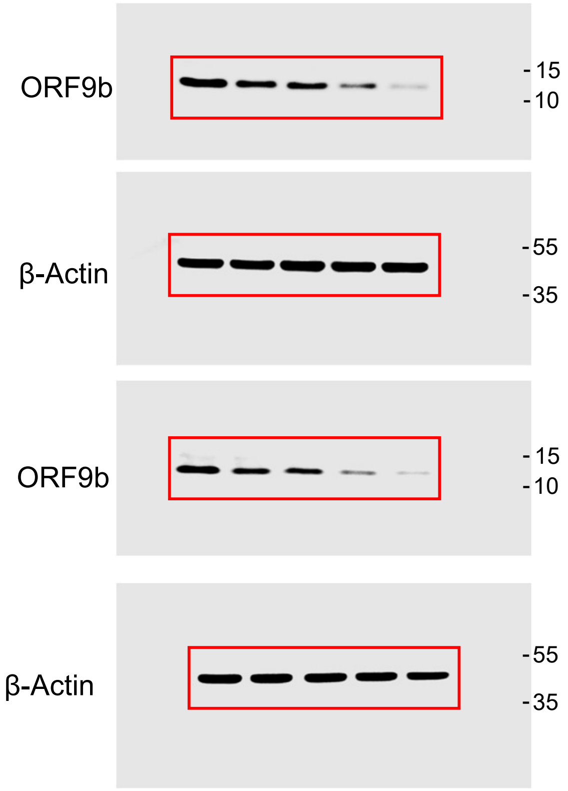

Fig. S7f

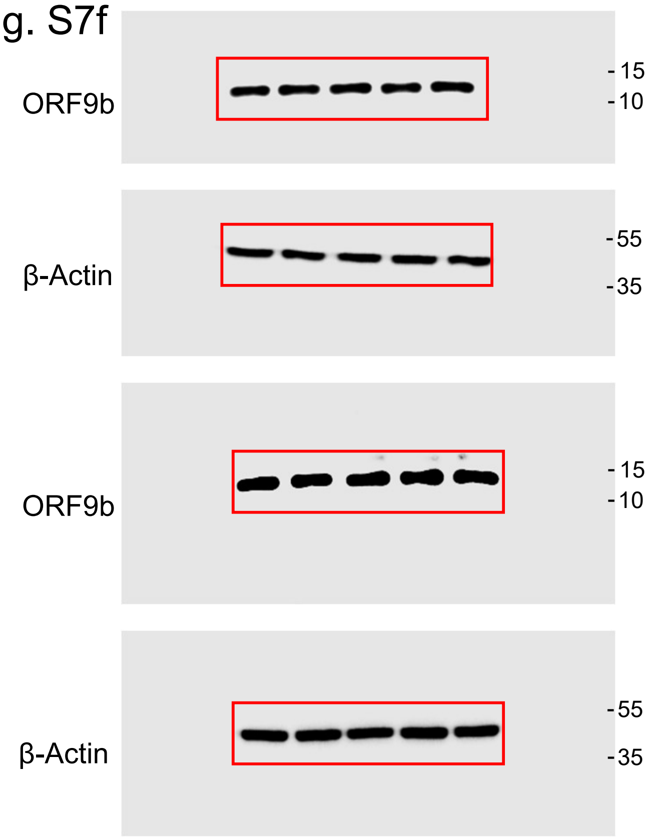

Fig. S7g

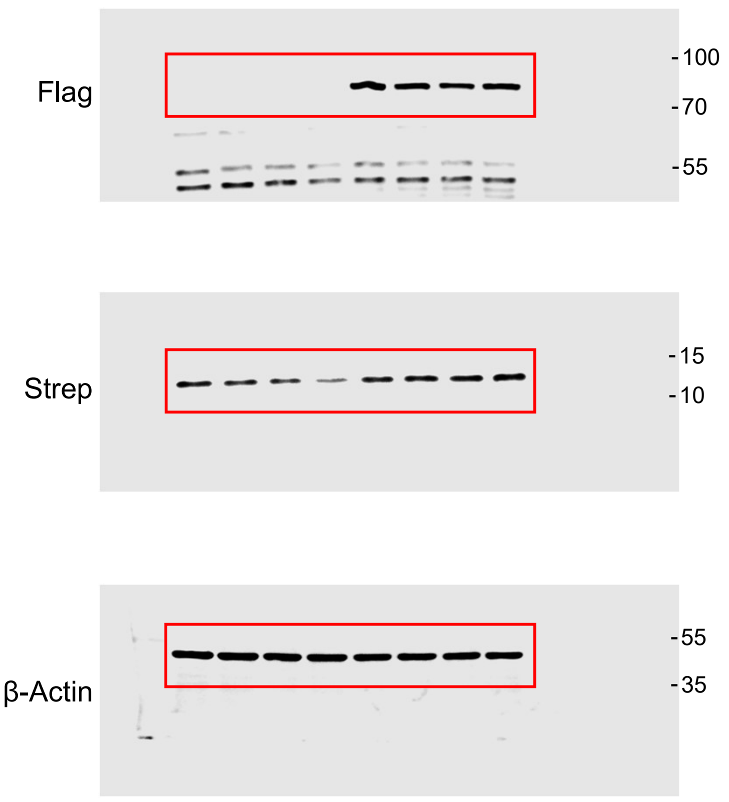

Fig. S7h

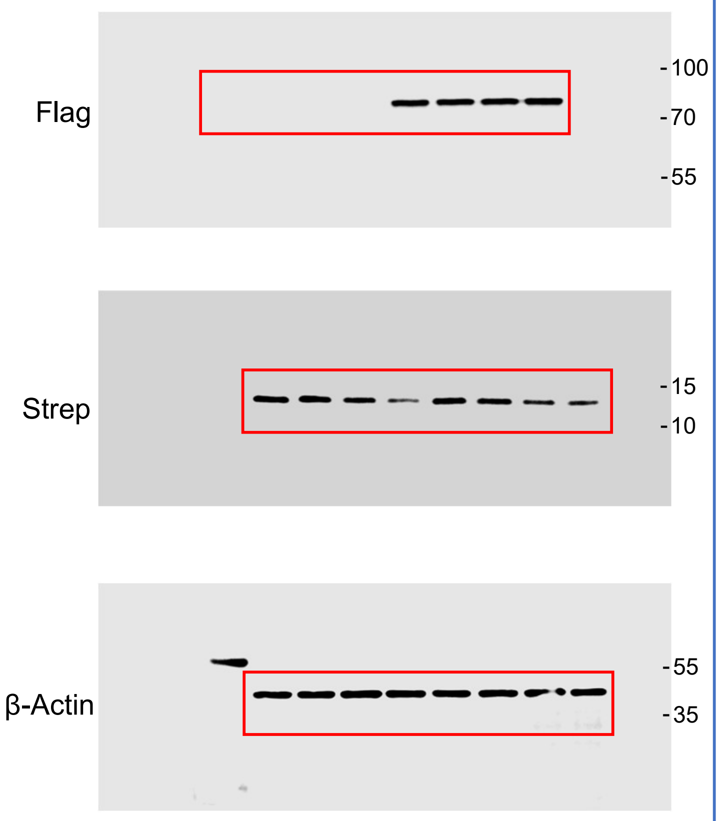

Fig. S8a

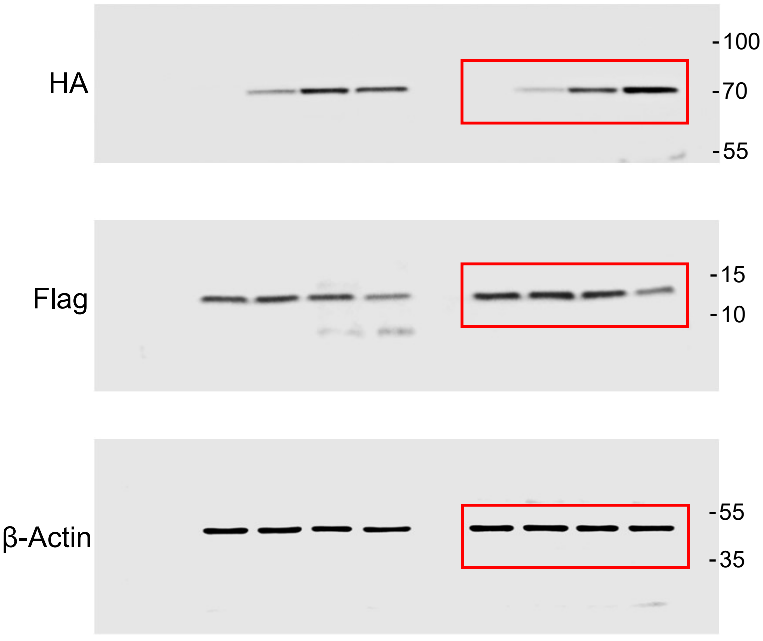

Fig. S8b

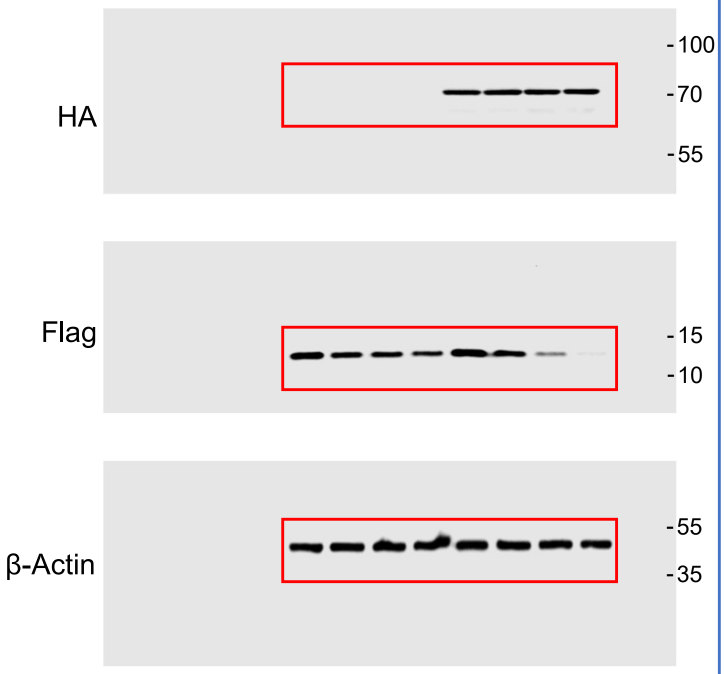

Fig. S8c—left 1

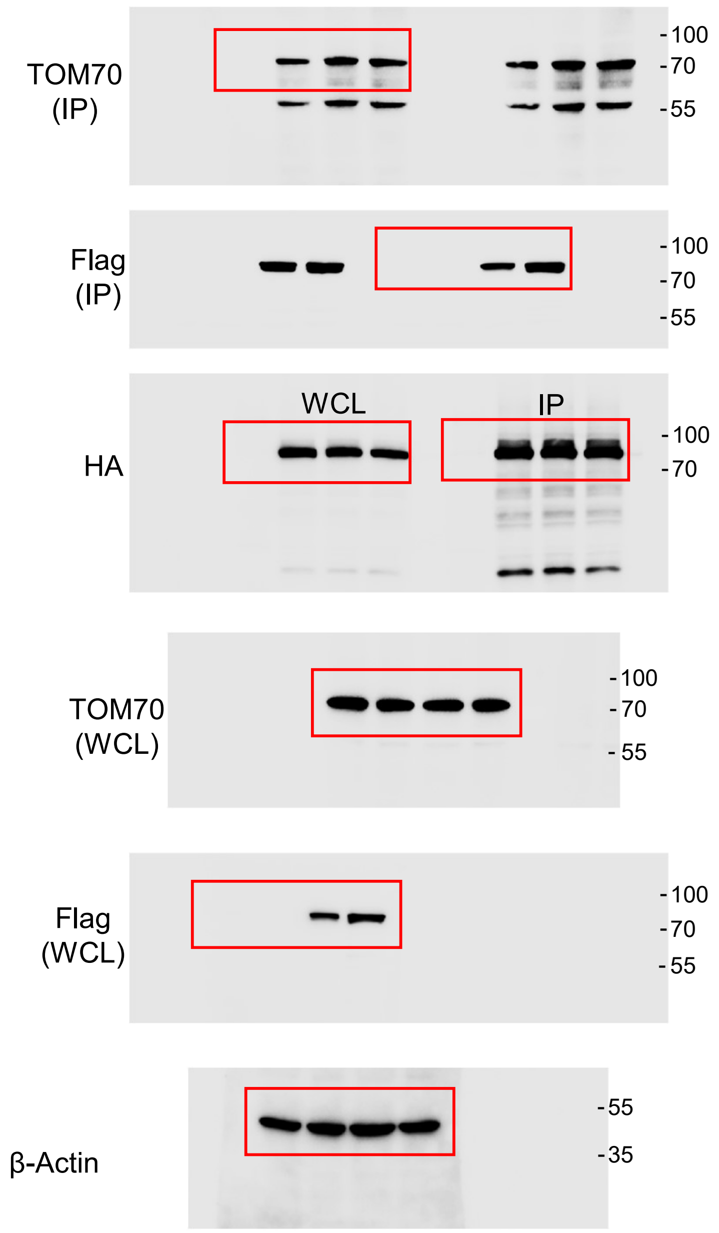

Fig. S8c-left 2

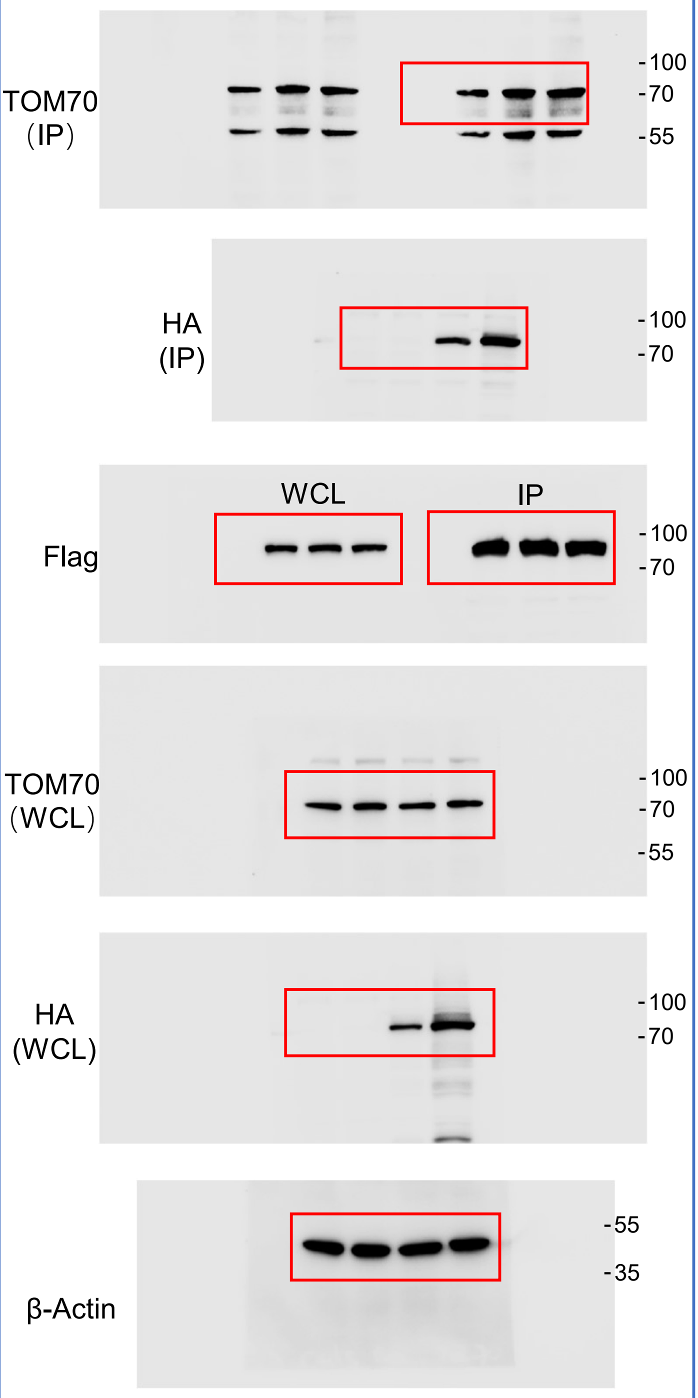

Fig. S8c-left 3

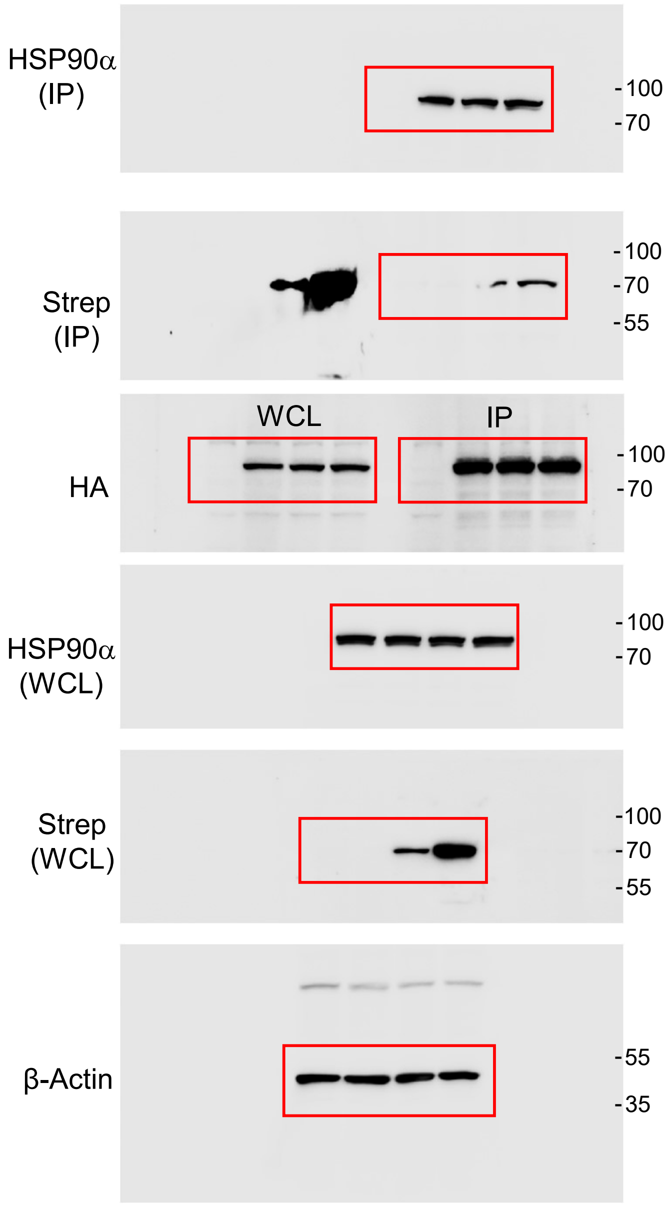

Fig. S8c-right 1

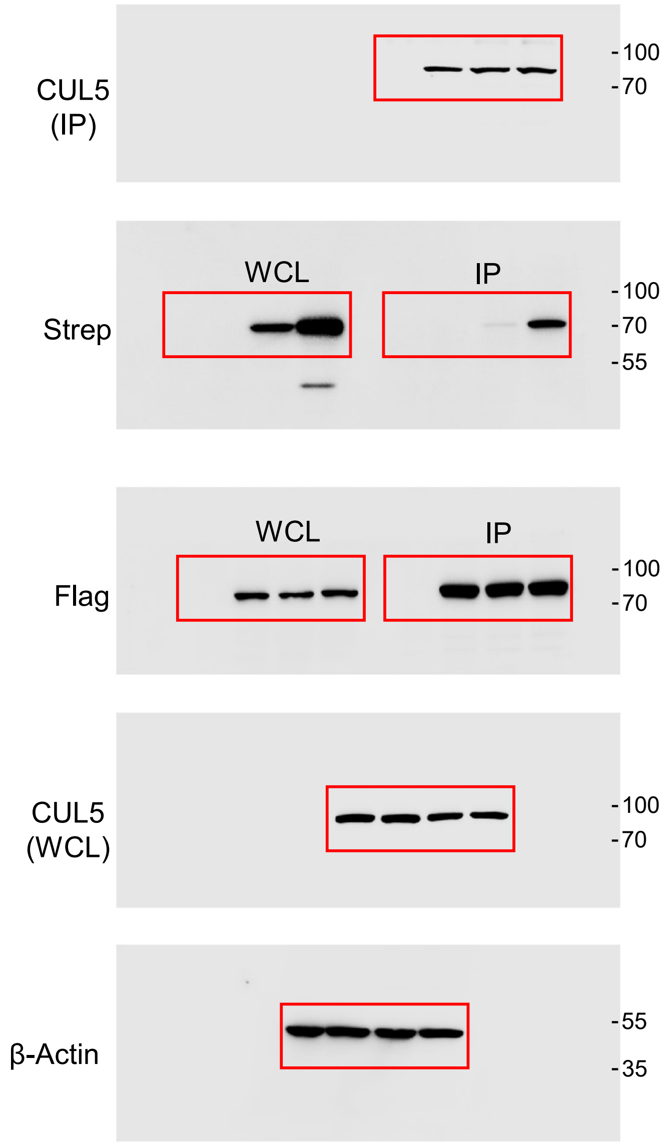

Fig. S8d

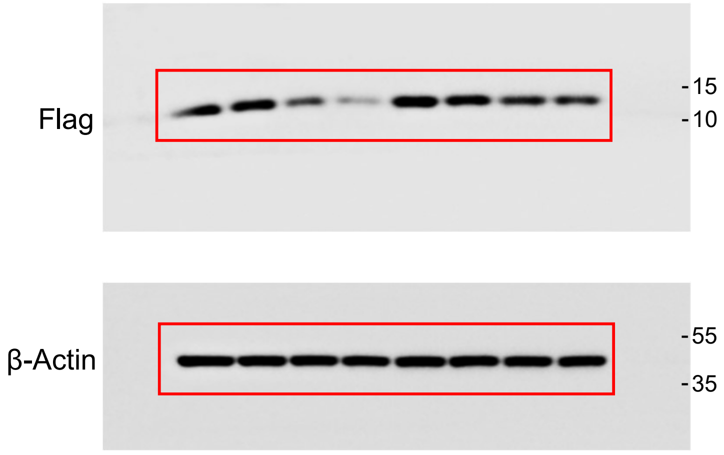

Fig. S8e

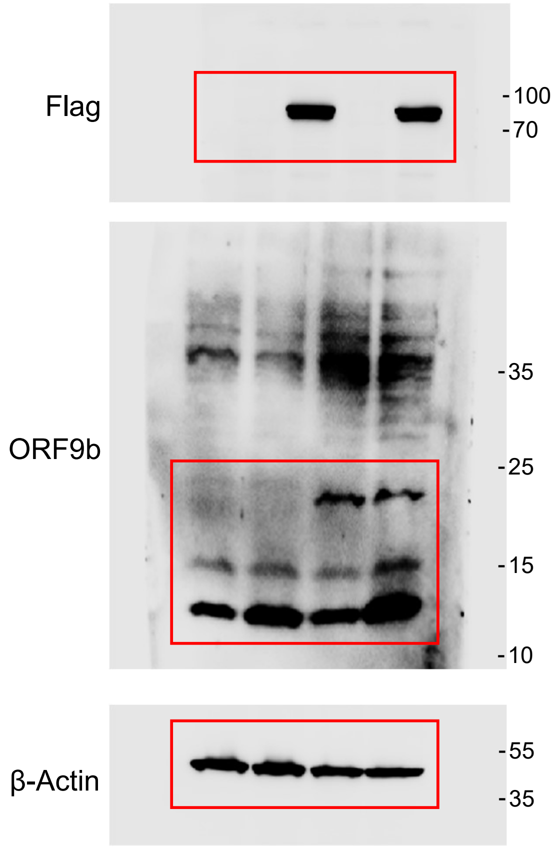

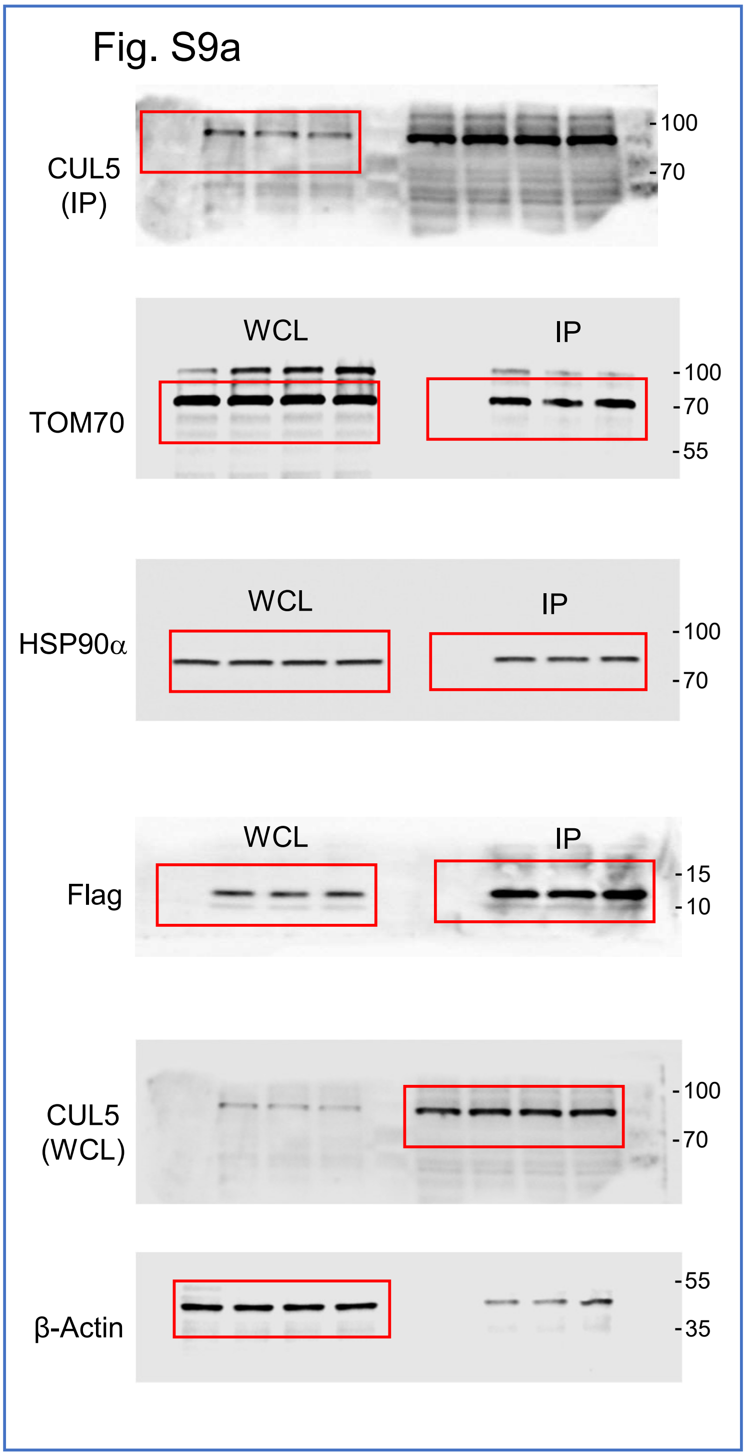
Fig. S9a
CUL5
(IP)
100
70
WCL
IP
TOM70
100
70
55
WCL
IP
HSP90α
100
70
WCL
IP
Flag
15
10
CUL5
(WCL)
100
70
β-Actin
55
35

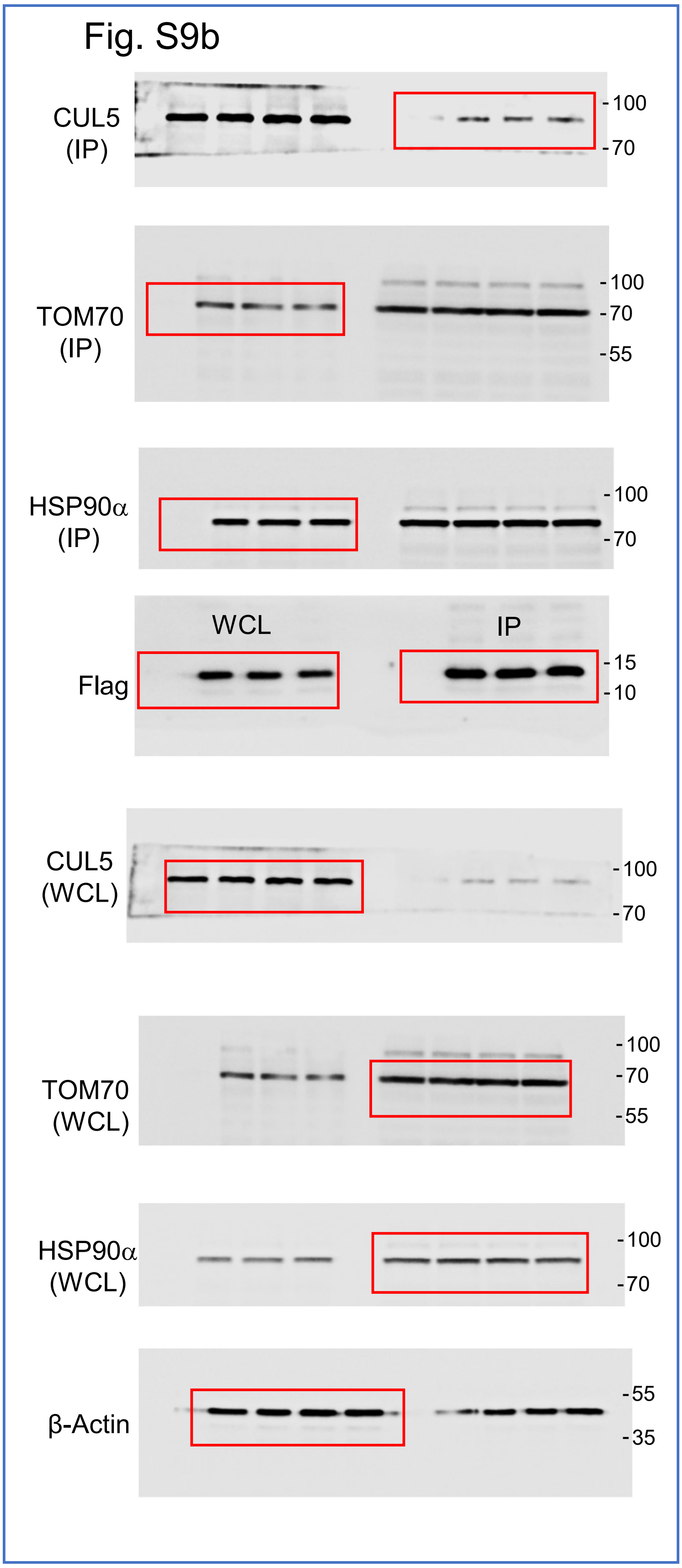
Fig. S9b
CUL5
(IP)
100
70
TOM70
(IP)
100
70
55
HSP90α
(IP)
100
70
WCL
IP
Flag
15
10
CUL5
(WCL)
100
70
TOM70
(WCL)
100
70
55
HSP90α
(WCL)
100
70
β-Actin
55
35

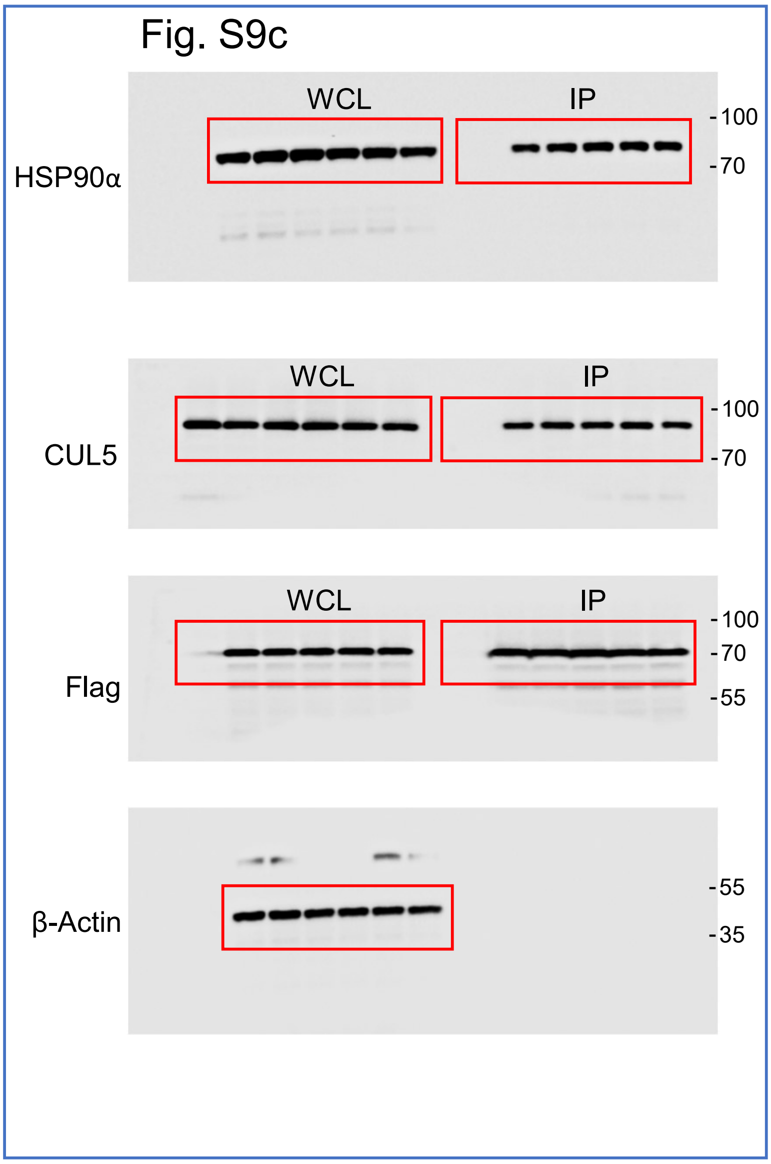
Fig. S9c
WCL
IP
HSP90α
100
70
CUL5
Flag
55
β-Actin
35

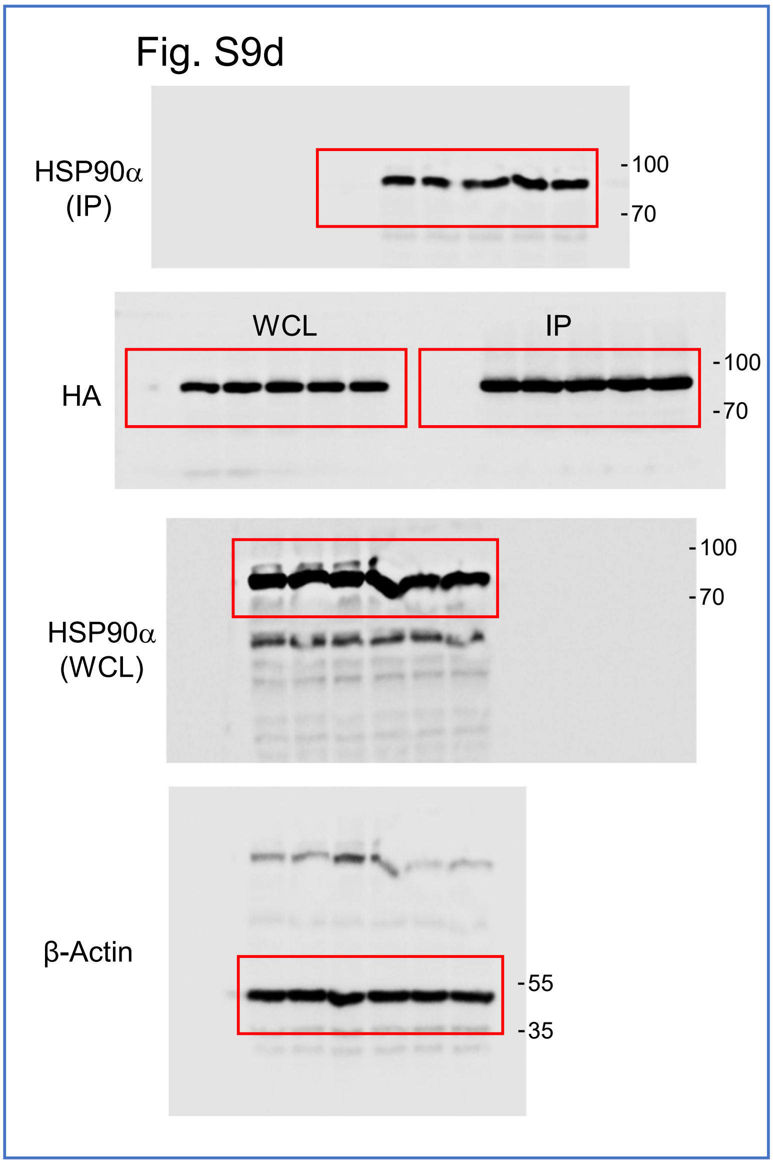
Fig. S9d
HSP90α (IP)
100
70
HA
WCL
IP
HSP90α (WCL)
β-Actin
55
35

Fig. S10a

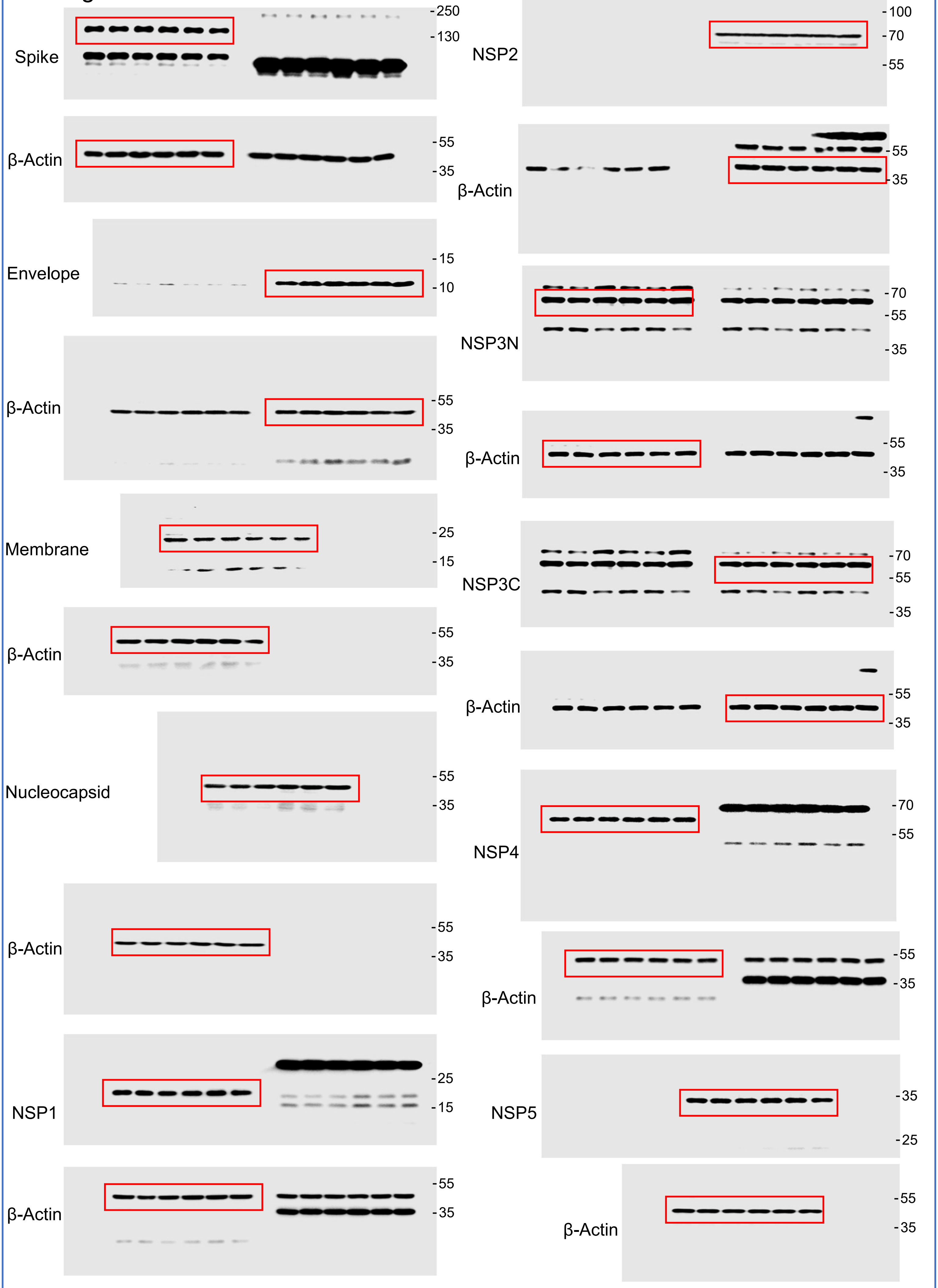

Spike
250
130
β-Actin
55
35
Envelope
15
10
β-Actin
55
35
Membrane
25
15
β-Actin
55
35
Nucleocapsid
55
35
β-Actin
55
35
NSP1
25
15
β-Actin
55
35
NSP2
100
70
55
β-Actin
55
35
NSP3N
70
55
35
β-Actin
55
35
NSP3C
70
55
35
β-Actin
55
35
NSP4
70
55
β-Actin
55
35
NSP5
35
25
β-Actin
55
35

Fig. S10a

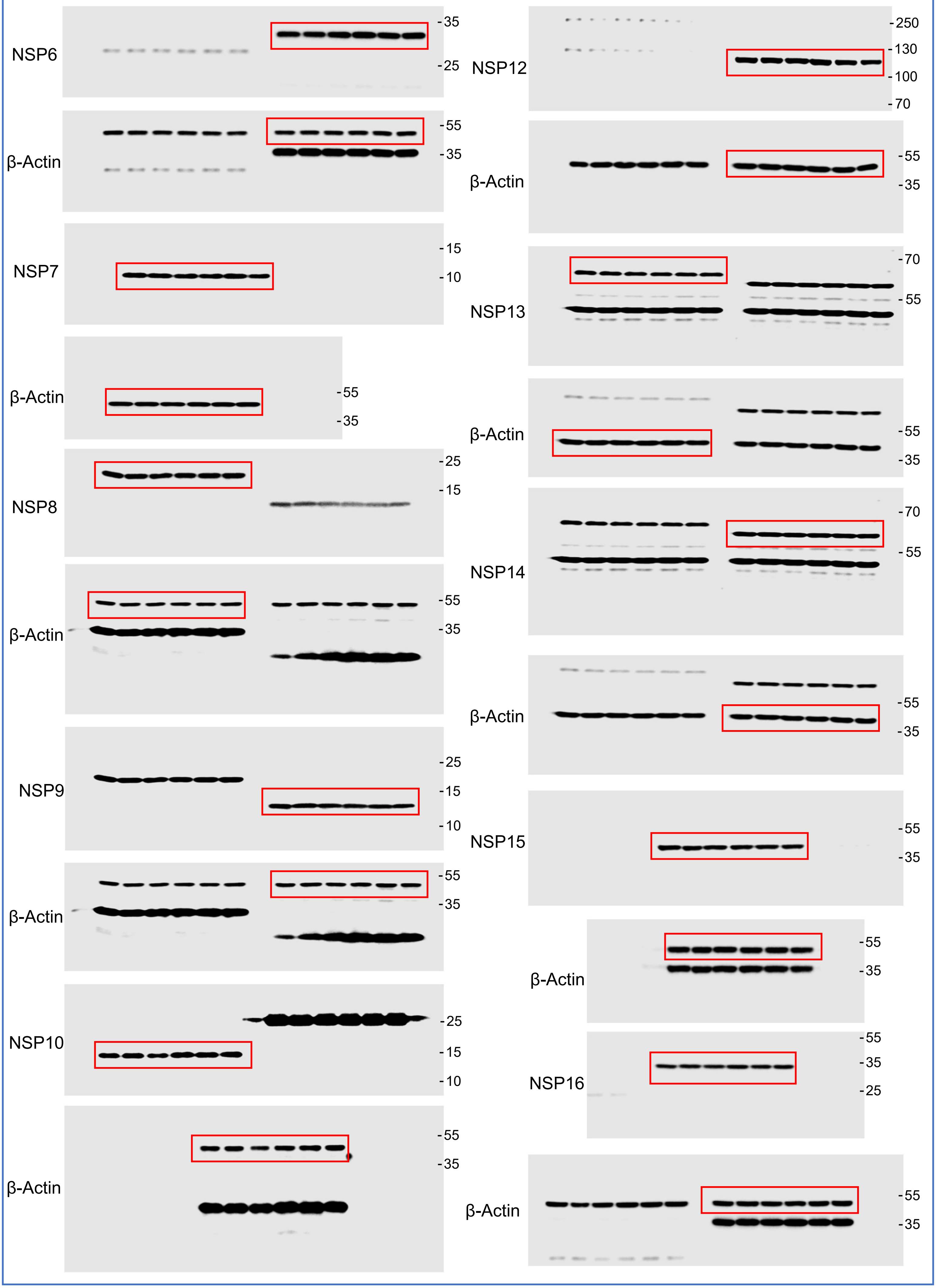
NSP6
β-Actin
NSP7
β-Actin
NSP8
β-Actin
NSP9
β-Actin
NSP10
β-Actin
NSP12
β-Actin
NSP13
β-Actin
NSP14
β-Actin
NSP15
β-Actin
NSP16
β-Actin
250
130
100
70
55
35
25
15
10

Fig. S10a

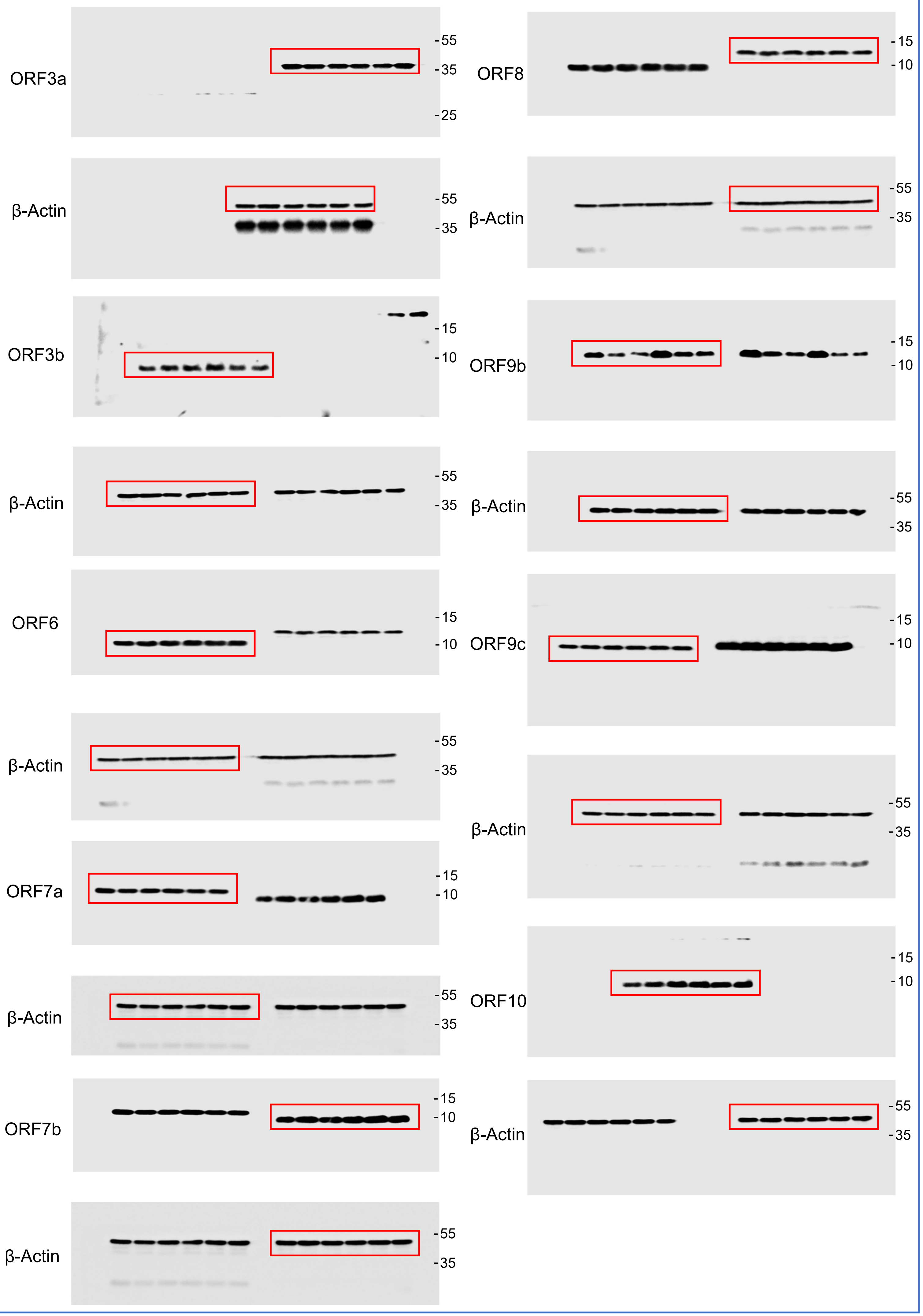
ORF3a
55
35
25
β-Actin
55
35
ORF3b
15
10
β-Actin
55
35
ORF6
15
10
β-Actin
55
35
ORF7a
15
10
β-Actin
55
35
ORF7b
15
10
β-Actin
55
35
ORF8
15
10
β-Actin
55
35
ORF9b
15
10
β-Actin
55
35
ORF9c
15
10
β-Actin
55
35
ORF10
15
10
β-Actin
55
35

Fig. S10e

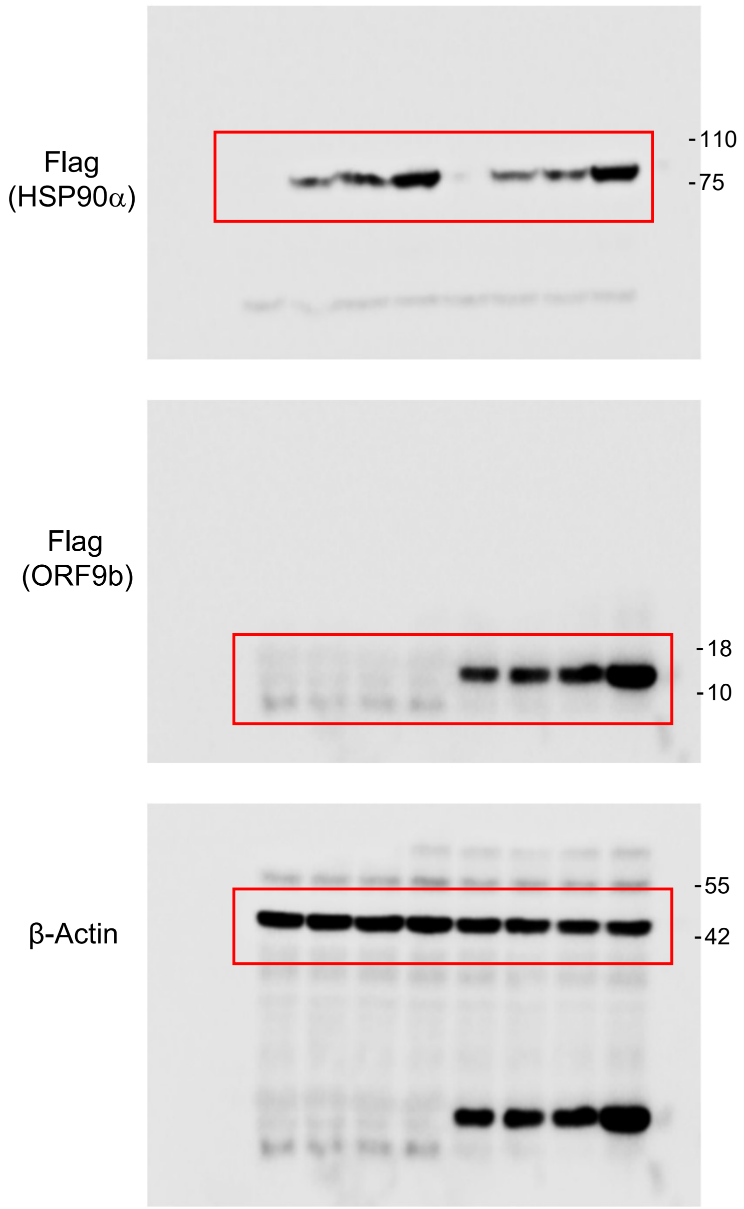

Supplement: Supplementary file 2 — Uncropped Western Blots [file 41392_2024_1874_MOESM2_ESM.pdf]
